# Interspecific complementation-restoration of phenotype in *Arabidopsis cuc2cuc3* mutant by sugarcane *CUC2* gene

**DOI:** 10.1186/s12870-022-03440-z

**Published:** 2022-01-22

**Authors:** Mohammad Aslam, Zeyuan She, Bello Hassan Jakada, Beenish Fakher, Joseph G. Greaves, Maokai Yan, Yingzhi Chen, Ping Zheng, Yan Cheng, Yuan Qin

**Affiliations:** 1 Guangxi Key Lab of Sugarcane Biology, State Key Laboratory for Conservation and Utilization of Subtropical Agro-Bioresources, College of Agriculture, Guangxi University, 530004 Nanning, Guangxi China; 2 Center for Genomics and Biotechnology, Fujian Provincial Key Laboratory of Haixia Applied Plant Systems Biology, 350002 Fuzhou, Fujian China

**Keywords:** *Saccharum spontaneum*, *cuc2cuc3*, organ boundary, *CUP-SHAPED COTYLEDON (CUC)*, Complementation-restoration

## Abstract

**Background:**

In plants, a critical balance between differentiation and proliferation of stem cells at the shoot apical meristem zone is essential for proper growth. The spatiotemporal regulation of some crucial genes dictates the formation of a boundary within and around budding organs. The boundary plays a pivotal role in distinguishing one tissue type from another and provides a defined shape to the organs at their developed stage. NAM/CUC subfamily of the NAC transcription factors control the boundary formation during meristematic development.

**Results:**

Here, we have identified the *CUP-SHAPED COTYLEDON (CUC)* genes in sugarcane and named *SsCUC2* (for the orthologous gene of *CUC1* and *CUC2*) and *SsCUC3*. The phylogenetic reconstruction showed that SsCUCs occupy the CUC2 and CUC3 clade together with monocots, whereas eudicot CUC2 and CUC3 settled separately in the different clade. The structural analysis of *CUC* genes showed that most of the *CUC3* genes were accompanied by an intron gain during eudicot divergence. Besides, the study of *SsCUC*s expression in the RNA-seq obtained during different stages of ovule development revealed that *SsCUCs* express in developing young tissues, and the expression of *SsCUC2* is regulated by miR164. We also demonstrate that SsCUC2 (a monocot) could complement the *cuc2cuc3* mutant phenotype of *Arabidopsis* (eudicot).

**Conclusions:**

This study further supports that *CUC2* has diverged in *CUC1* and *CUC2* during the evolution of monocots and eudicots from ancestral plants. The functional analysis of *CUC* expression patterns during sugarcane ovule development and ectopic expression of *SsCUC2* in *Arabidopsis* showed that SsCUC2 has a conserved role in boundary formation. Overall, these findings improve our understanding of the functions of sugarcane *CUC* genes. Our results reveal the crucial functional role of *CUC* genes in sugarcane.

**Supplementary Information:**

The online version contains supplementary material available at 10.1186/s12870-022-03440-z.

## Background

In plants, different tissues and organs are produced in a specific pattern during embryonic and post-embryonic developments [1]. Shoot apical meristem (SAM) mainly constitutes the vegetative and floral meristem. Unlimited (or indeterminate) growth in vegetative tissues produces leaves and axillary branches at the nodes, whereas the limited (or determinate) growth is marked by the floral meristem where the SAM partitions its cells to divide in a highly coordinated manner to give rise to different floral parts [2]. The spatiotemporal differentiation of shoot apical cells forms specific patterns of meristems, initiated under tight regulation of boundary cells [3–9]. The regulatory network constitutes combinatory action of several elements, including transcription factors (TFs) [10]. The NAC transcription factors belong to one of the most prominent families of plant-specific TFs, constituting more than 100 members in *Arabidopsis* [11]. NACs derived their name from *NAM* (*NO APICAL MERISTEM*), *ATAF 1/2 *(Arabidopsis transcription activation factor) and *CUC *(*CUP-SHAPED COTYLEDON*) [12–14]. The members of NAC transcription factors, including *CUC* in *Arabidopsis, NAM* in *Petunia hybrida* and *CUPULIFORMIS (CUP)* in *Antirrhinum majus*, are responsible for boundary cell formation [1, 15, 16]. In *Arabidopsis*, three *CUC* genes, *CUC1, CUC2* and *CUC3*, are reported. *CUC1* and *CUC2* have diverged from each other through duplication event from a common ancestor. *CUC2* defines the shoot apical meristem zone, whereas *CUC3* marks the boundary layer around developing organs. In general, primary shoot meristem development depends on CUCs activity, and axillary meristem requires mainly CUC2 and CUC3. Mutants of *CUC* genes revealed various exclusive and partially overlapping phenotypes in different plant development processes such as SAM establishment, lateral organ separation, leaf serration, and ovule formation [1, 17–19]. These mutant phenotypes suggest that CUCs are necessary for normal ovule development and *CUCs* have higher expression in sporogenous tissue of ovule [20–31]. The low expression of *CUC*s decreases the number of leaflets causing fewer serrated leaves [10, 18, 32–34]. The expression pattern of *CUC*s is often synchronous and redundant in many cases; therefore, it becomes difficult to recognize a single knockout mutation. For example, defects in shoot apical meristem and cotyledon shapes can only be detectable under double and triple mutation of CUCs in *Arabidopsis* [17].

Previous findings indicate that *Arabidopsis CUC1*, *CUC2* and rice *CUC1* are post-transcriptionally targeted by miR164 [18, 35, 36]. This regulatory mechanism controls the balance between tissue separation/fusion and plays essential roles during leaf serration, phyllotaxy and growth [18, 37]. Consistently, the plants display pronounced serrations, extra petals, and enlarged boundary domains when they express a miR164-resistant version of *CUC1* or *CUC2* [35]. Intriguingly, the role of the *miR164-CUC* module in the regulation of leaf and floral organ morphology seems conserved across species [19, 37]. Besides miRNA, *CUC* gene expression is also reported to be regulated by chromatin remodeling [38]. Two SWI/SNF ATPases, AtBRM and SYD, are necessary for cotyledon separation in *Arabidopsis* by modulating the expression of *CUC* genes [38].

*Saccharum* spp. (sugarcane), a tropical grass belonging to the family *Poaceae* is cultivated worldwide as a crop for sugar and biofuel feedstock. Sugarcane provides approximately 80% of sugar and 40% of bioethanol [39]. In terms of tonnage, it is the most cultivated crop than other staple crops (rice and wheat) (FAO, UN). Generally, the commercial cultivars are planted using stem cuttings with approximately 3-4 axillary buds that form primary shoots and roots after planting [40]. Due to the increasing demand for biofuel, researchers are putting continuous effort into improving sugarcane varieties for more sugar content while maintaining their hardiness. Recently, the sugarcane genome, one of the most complex genomes among all the crops, has been published, allowing researchers to study and improve the commercial varieties [41]. Here, we performed a genome-wide identification and expression analysis of *CUC* genes from sugarcane to classify and better understand their functions. We found two *CUC*, *SsCUC2* and *SsCUC3* genes, in the sugarcane genome with unique expression patterns. We also found that the expression level of *SsCUC2* is regulated by miR164, which could be essential for sugarcane ovule development. Besides, we discovered that *SsCUC2* could complement the cotyledon fusion and axillary meristem defects of the *Arabidopsis cuc2cuc3* mutant. Despite the divergence of monocots and eudicots during evolution, the complementation of CUC2 from sugarcane (monocot) in *Arabidopsis* (eudicot) suggests a conserved role of *Ss*CUC2. Our results indicate that SsCUCs play a central role in sugarcane development, and *miR164-SsCUC2* module could be essential for sugarcane ovule development.

## Results

### Identification of the CUC genes in *S. spontaneum*

A total of five sugarcane *CUC* gene sequences (three *CUC2* alleles and two *CUC3* alleles) were identified in the *S. spontaneum* genome (Table 1). The distribution of these *SsCUC* genes was on chromosomes 6 and 7. The SsCUC proteins were 311-401 amino acid (aa) residues in length, where *CUC2* alleles had 397, 400 and 401 aa residues, and *CUC3* alleles were with 311 and 331 aa residues, respectively (Table 1). The molecular weight (MW) for the SsCUC proteins ranged from 34.198 kDa to 42.251 kDa, and their isoelectric points (pI) varied from 7.22 to 9.03. Additional parameters for sugarcane SsCUC proteins such as subcellular locations, N-glycosylation sites, and phosphorylation sites have also been predicted in this study. Based on the previous reports, the SsCUC proteins were expected for their localization in the nucleus, suggesting that they participate in gene expression regulation [42]. Interestingly, N-glycosylation sites were not present in SsCUC2, whereas both the SsCUC3 had one N-glycosylation site at their C terminal end. Although both SsCUC2 and SsCUC3 had phosphorylation sites in the NAM domain, SsCUC2 possessed extra phosphorylation sites in the NAM domain. Besides, SsCUC3 did not own any phosphorylation sites outside the NAM domain whereas, SsCUC2 had 2 to 3 extra phosphorylation sites outside the NAM domain in the C-terminal region (Table -1, Additional File S1).

**Table 1:** The physicochemical properties of CUC sequences in *S. spontaneum and Arabidopsis thaliana*

	Name	Gene ID	A	B	C	Amino acid length (aa)	MW (kDa)	Isoelectric point	Chromosome position
1	SsCUC2	Sspon.07G0020380-1A	0	4	2	401	42.251	8.76	Chr7A:75915862-75918375
2	SsCUC2	Sspon.07G0020380-2C	0	4	2	400	42.107	8.9	Chr7C:70928956-70931222
3	SsCUC2	Sspon.07G0020380-3D	0	4	3	397	41.452	9.03	Chr7D:67919120-67921641
4	SsCUC3	Sspon.06g0001780-1A	1	3	0	331	36.215	7.22	Chr6A:5729607-5731660
5	SsCUC3	Sspon.06g0001780-2B	1	3	0	311	34.198	8.52	Chr6B:3987180-3989885
6	AtCUC1	AT3G15170	3	4	1	310	34.232	8.58	Chr3:5109782-5111608
7	AtCUC2	AT5G53950	3	5	2	375	41.434	8.52	Chr5:21901704-21903854
8	AtCUC3	AT1G76420	3	3	3	334	38.017	6.46	Chr1:28671806-28674045

note:

‘A’ represents the number of N-Glycosylation site

‘B’ represents the number of Protein kinase C phosphorylation site in the NAM domain of the protein

‘C’ represents the number of Protein kinase C phosphorylation site outside the NAM domain of the protein

Alleles are labeled with –nX, where n represents alleles and X indicates haplotype ID, ranging from A to D

### Gene structure and motif composition of CUC genes

**Fig. 1:**
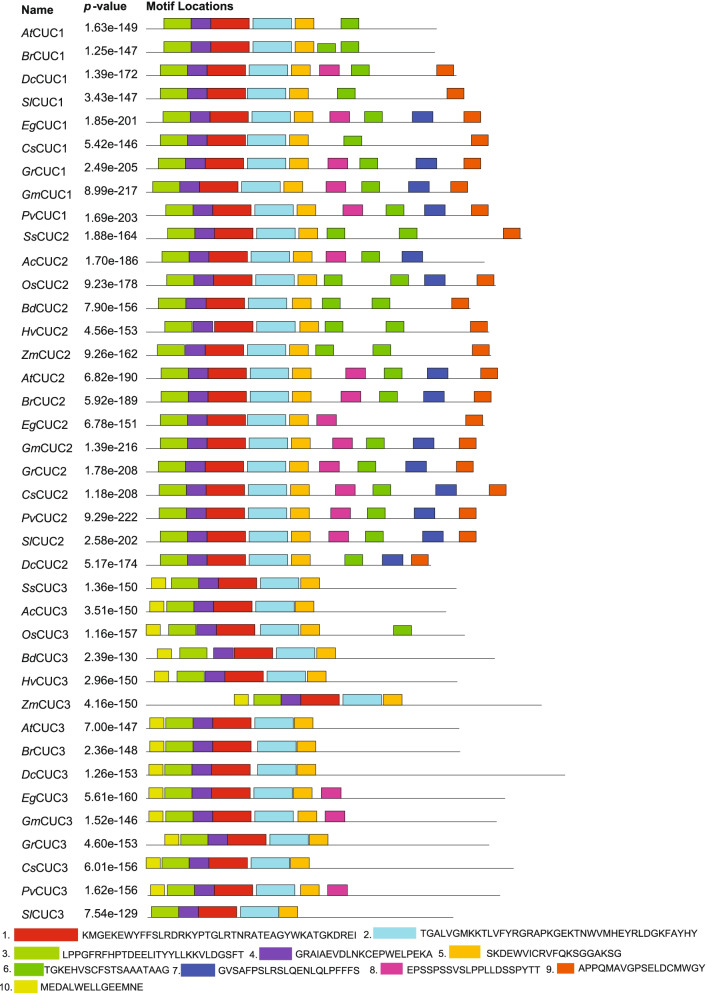
Schematic diagram representing the architecture and motif composition CUC genes in 6 monocots (Ss-*Saccharum spontaneum, Ac-Ananas comosus, Os-Oryza sativa, Bd-Brachypodium distachyon, Hv-Hordeum vulgare* and *Zm-Zea mays)* and 9 eudicots *(At-Arabidopsis thaliana, Br-Brassica rapa, Dc-Daucus carota, Sl-Solanum lycopersicum, Eg-Eucalyptus grandis, Cs-Citrus sinensis, Gr-Gossypium raimondi, Gm-Glycine max* and *Pv-Phaseolus vulgaris*). The motifs, numbers 1–10, are displayed in different colored boxes and detailed below

The intron/exon organization and conserved motifs of the *CUC* genes from selected monocots and eudicots, including *S. spontaneum*, were studied to explore the structural features of *CUC* genes and proteins encoded by the *CUC* genes. We identified ten different motifs in CUC proteins (Fig. 1). Among the 10 identified motifs, SsCUC2 had 8 motifs and SsCUC3 only had 6 motifs. Motif no. 8 and 10 were not present in the SsCUC2 whereas, motifs 6, 7, 8 and 9 were absent from SsCUC3 (Fig. 1). All the CUC3 proteins except SlCUC3 had motif 10, indicating that it could be CUC3 proteins specific motif. Motifs 7 and 9 were specific to CUC1 and CUC2 (Fig. 1). Motifs 1 to 5 were present in all the selected CUC proteins and represented the NAM domain.

**Fig. 2:**
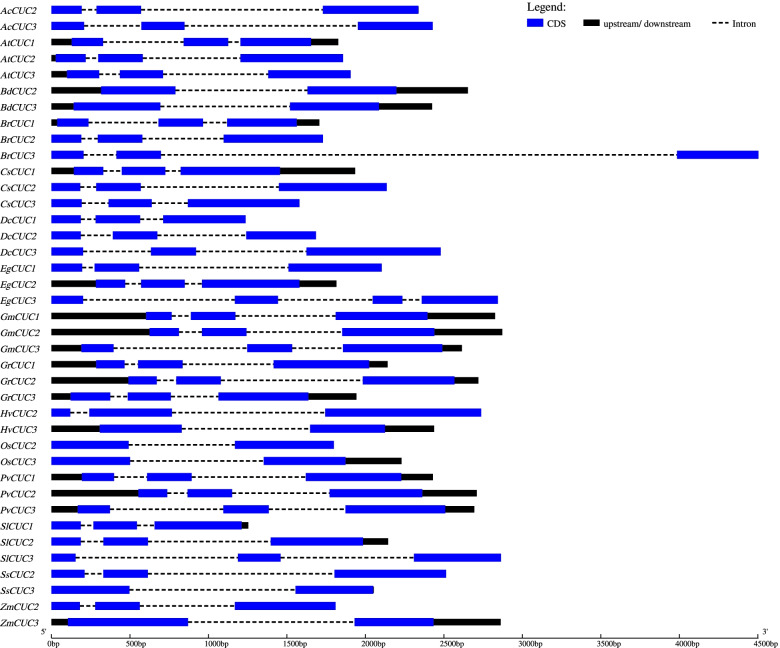
Exon-intron structure of sugarcane *SsCUC2*, *SsCUC3* and their selected homologous genes. Exons are represented as blue boxes, intron as dotted lines, and UTR are represented with black boxes. The different species are indicated as: Ss-*Saccharum spontaneum, Ac-Ananas comosus, Os-Oryza sativa, Bd-Brachypodium distachyon, Hv-Hordeum vulgare, Zm-Zea mays, At-Arabidopsis thaliana, Br-Brassica rapa, Dc-Daucus carota, Sl-Solanum lycopersicum, Eg-Eucalyptus grandis, Cs-Citrus sinensis, Gr-Gossypium raimondi, Gm-Glycine max* and *Pv-Phaseolus vulgaris*

The exon-intron organization of all of these *CUC* genes was scanned to gain more insights into the *CUC* gene evolution. In general, the *CUC* genes were intron-poor, and the number of introns varied from 1 to 3 for them. All the selected *CUC1* genes contained two introns, and most of the *CUC2* genes also had two introns except for *BdCUC2* and *OsCUC2*, those possessed only one intron (Fig. 2). All the selected monocot *CUC3* had only one intron except for the pineapple *CUC3 (AcCUC3)*, which had two introns indicating an intron gain during evolution. At the same time, all the eudicot *CUC3* had two introns except for the *Eucalyptus grandis CUC3 (EgCUC3)*, which had 3 introns (Fig. 2). Additionally, two genes (*AtCUC2* and *AtCUC3*) only had 5’ untranslated region (UTR), and three genes (*SlCUC1*/2 and *OsCUC3*) had only 3’ UTR whereas, 17 genes did not have any UTR. Altogether, these results suggest structural diversity among CUC genes (Fig. 2).

### Phylogenetic analysis of CUC proteins

**Fig. 3:**
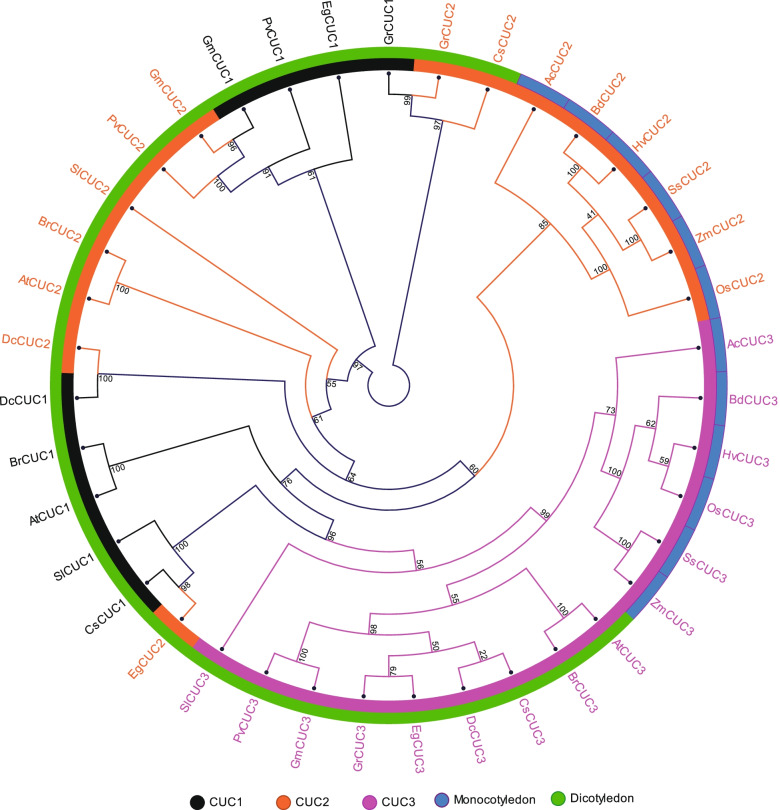
The neighbor-joining phylogenetic tree analysis of sugarcane SsCUC2, SsCUC3 and their homologous from 5 monocots (*Ac-Ananas comosus, Os-Oryza sativa, Bd-Brachypodium distachyon, Hv-Hordeum vulgare* and *Zm-Zea mays)* and 9 eudicots *(At-Arabidopsis thaliana, Br-Brassica rapa, Dc-Daucus carota, Sl-Solanum lycopersicum, Eg-Eucalyptus grandis, Cs-Citrus sinensis, Gr-Gossypium raimondi, Gm-Glycine max* and *Pv-Phaseolus vulgaris*). The tree was generated with a bootstrap value of 1000 generated in CLC sequence genomics workbench v12. CUC1 proteins are represented with black color, CUC2 proteins are represented with orange color and CUC3 proteins are represented with pink color. The sequence information of all the proteins used in the analysis is provided in Additional file S2

The evolution of CUC orthologs in different plant species was investigated by constructing a phylogenetic tree consisting of 39 CUC proteins from six monocots, including *S. spontaneum* and nine eudicots using the Neighbor-Joining (NJ) method (Fig. 3; Additional File S2). All the monocot CUC proteins were divided into two major groups, where one group represented monocot specific CUC2 and the other group represented monocot specific CUC3 proteins (Fig. 3). The eudicot CUC proteins were separated from monocots and grouped along with eudicot-specific CUCs. For example, eudicot CUC3 made a separate group with dicot-specific CUC3 proteins; however, it was still separated from eudicot CUC1 and CUC2 (Fig. 3).

### SsCUC2 has transcriptional activation ability

**Fig. 4:**
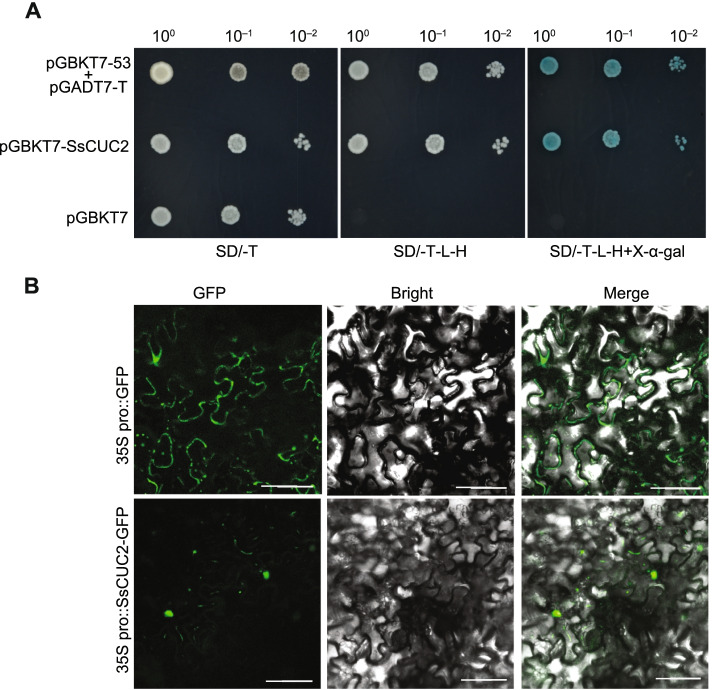
**A** Transcription activity assay of SsCUC2. The ORF of SsCUC2 was introduced into the yeast expression vector pGBKT7. Yeast cells cotransformed with pGBKT7-53 + pGADT7-T were used as the positive control, and yeast cells with empty vector pGBKT7 were used as a negative control. The yeast cultures harboring respective vectors were grown on the synthetic medium supplied with dextrose (SD) in the absence of Trp (SD/ -T, left panel), on SD medium in the absence of Leu, Trp, and His (SD/ -L -T -H, middle panel) and on SD medium with α-galactosidase and in the absence of Leu, Trp, and His (SD/ -L -T –H + X-α-gal, right panel). Yeast cells were incubated until OD_600_ reached 1 and then diluted 10- and 100-fold for assays. **B** Subcellular localization of SsCUC2 in tobacco epidermal cells (lower panel)

The NAC family genes are highly conserved in plants, and it is involved in transcriptional regulation of many genes [11]. We used a GAL4-responsive reporter system to conduct a transient expression assay in yeast cells to verify the transcription activation feature of SsCUC2. Transformed yeast cells with the positive control (pGBKT7-53 + pGADT7-T) and (pGBKT7-SsCUC2) grew well on synthetic dropout medium without tryptophan, histidine and leucine [SD (-Trp/-His/-Leu)] and ensured α-galactosidase (α-gal) activity. Yeast cells with empty pGBKT7 (negative control) exhibited no growth and α-gal activity, indicating that SsCUC2 performs as transcription factors (Fig. 4 A). In addition, to determine the subcellular location of SsCUC2, we used 35 S:SsCUC2-GFP fusion construct and transiently expressed the construct in *N. benthamiana* leaves. The results showed that SsCUC2 gets localized to the nucleus (Fig. 4B).

### The expression of *SsCUC2* and *SsCUC3* is differentially regulated during sugarcane female gametophyte development

**Fig. 5:**
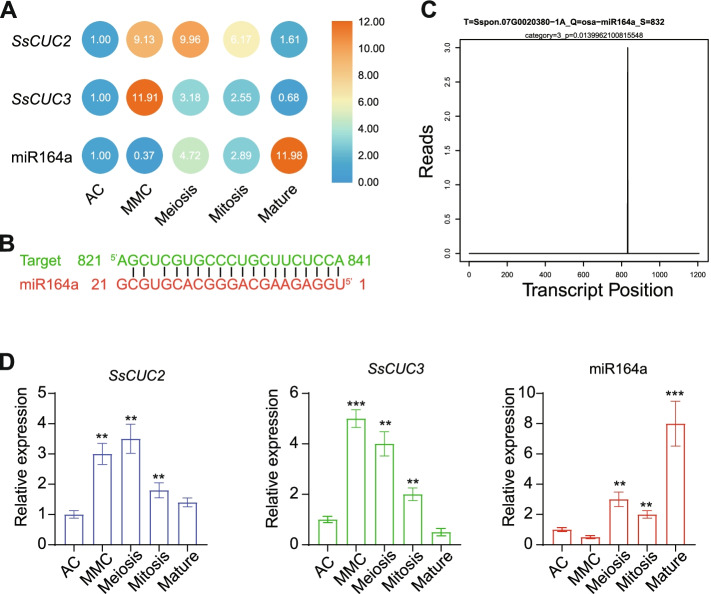
Expression and regulation of sugarcane *CUC* genes. **A** The expression patterns of *SsCUC2*, *SsCUC3* and miR164a in different developmental stages of ovules. The expression level for the AC stage was set to 1.0, and the data presented in MMC, Meiosis, Mitosis and Mature is relative to AC. **B** Predicted target site in *SsCUC2* by psRNATarget database. **C** Target plot (T-plot) indicating the cleavage event mediated by miR164 on the PARE-seq. **D** RT-qPCR of *SsCUC2*, *SsCUC3* and miR164a in different developmental stages of ovules. Sugarcane *β-actin* was used as an internal control. The expression level was set to 1.0 (for AC), and the data presented relative transcript abundance to AC. The threshold cycle (CT) values were used to calculate relative expression using the 2^−ΔΔCT^ method. The data represent the means ± SE of three replicates, and asterisks denote statistically significant values (**p < 0.01 and *** p < 0.001)

Previous research reports have implicated CUC1 and CUC2 in ovule formation and development, besides the mutant of CUC1 and CUC2 results in the reduction of ovule number [43, 44]. During ovule development, the expression of *CUC* genes has been observed in medial, placental tissues and between the region of outgrowing ovules in *Arabidopsis* [44]. Therefore we analyzed the RNA-seq from different stages of sugarcane ovule development to study the expression patterns of *SsCUC2* and *SsCUC3*. Consistent with previous reports, we found the differential expression of *SsCUC2* and *SsCUC3* during the sugarcane ovule development (Fig. 5 A). The expression of both the *SsCUC2* and *SsCUC3* increased tremendously at the megaspore mother cell (MMC) stage, which gradually decreased in the meiosis and mitosis stages. The expression of *SsCUC2* and *SsCUC3* return to approximately basal level at the mature stage of ovule development (Fig. 5 A). The differential expression patterns of *SsCUC2* and *SsCUC3* indicate that the CUC genes might also regulate the sugarcane ovule development.

### miR164a negatively regulates *SsCUC2*

Previously, it has been demonstrated that miR164 post-transcriptionally regulates the *CUC1* and *CUC2* [18, 36, 45]. Therefore, we first checked whether *SsCUC2* ( the orthologous gene of *CUC1* and *CUC2*) expression is regulated by microRNA. Consequently, we investigated the corresponding miRNA using *SsCUC2* as a target in the psRNATarget database (http://plantgrn.noble.org/psRNATarget/home) [46]. The results indicated that the microRNA miR164a might regulate *SsCUC2* (Fig. 5B), which could also be regulating *SsCUC2* during ovule development. Hence we checked the expression of miR164a in sRNA-seq from different stages of sugarcane ovule development. The sRNA-seq result suggested that the sugarcane miR164a was differentially accumulated in the developing sugarcane ovule (Fig. 5 A). The expression pattern of miR164a was initially decreased in the MMC stage and gradually increased in meiosis and mitosis stages, but the expression of miR164 exponentially increased at the mature stage (Fig. 5 A). The expression pattern of miR164a in developing ovules endorses that it could be regulating the *SsCUC2* expression during ovule development. We further confirmed that miRNA164a cleaves the *SsCUC2* using the PARE-seq generated (Fig. 5 C). We then checked the consistency of deep sequencing and validated the results using RT-qPCR. RT-qPCR results showed a similar trend to that of the sequencing data (Fig. 5D).

### SsCUC2 has a conserved function in meristem/organ boundary specification

**Fig. 6:**
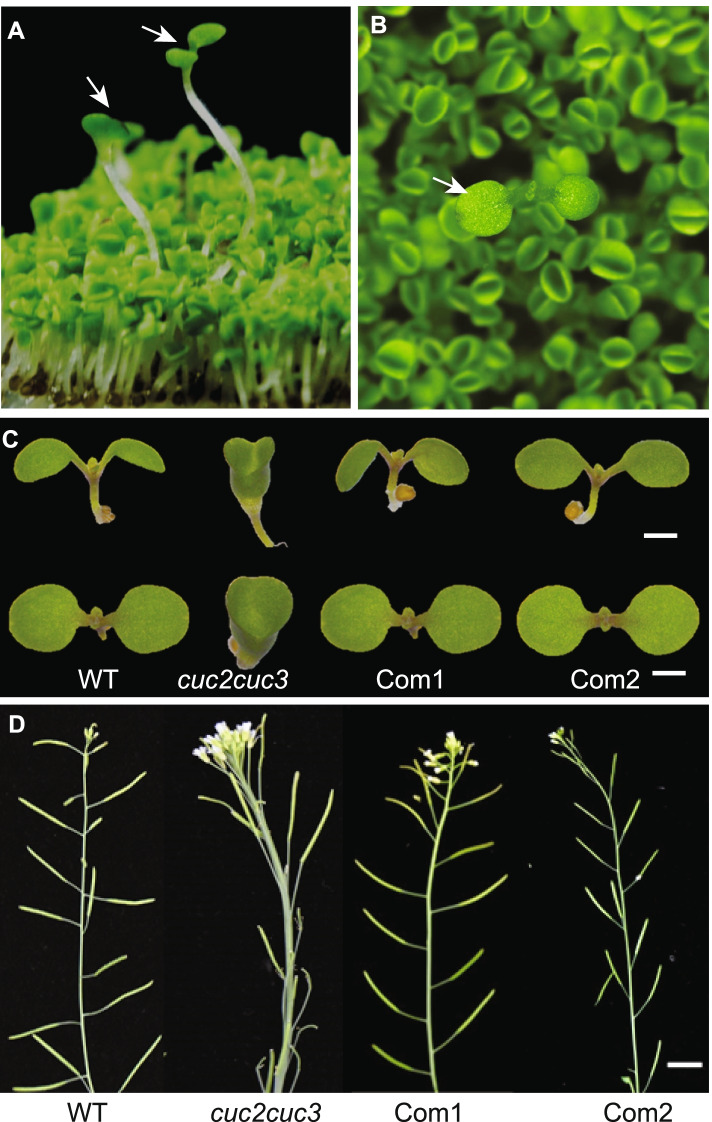
Functional characterization of sugarcane *CUC2* gene. A&B) Hygromycin-based screening of T1 transgenic plants. The plants carrying the *SsCUC2* gene show normal two separate cotyledons (marked with white arrows), whereas the non-transformed with the cup-shaped fused cotyledon. Two complemented T2 lines (Com1 and Com2) showing the rescue of mutant phenotype C) at the early developmental stage with two separate cotyledons D) at the reproductive stage with normal inflorescences

In arabidopsis, the double mutants of the CUC gene show defects in SAM formation and form cup-shaped cotyledon due to the fusion of cotyledons. However, the single mutants of any CUC genes have no significant boundary formation defects [1] (Additional File S3). To investigate the conserved function of CUC genes and check whether the SsCUC2 can rescue the phenotypic defects of *cuc2cuc3* mutant, we ectopically expressed the *SsCUC2* in the *Arabidopsis cuc2cuc3* mutant. During the transgenic screening on the hygromycin-based selection, we found that the plants carrying the sugarcane *CUC2* gene grew bigger with two separate cotyledons and complemented the cup phenotype of *cuc2cuc3* mutant (Fig. 6 A and 6B). The complemented lines (com1 and com2) showed normal plant growth with no SAM defects and two separate cotyledons in the next generation. These complemented plants showed a complete rescue of mutant phenotype during early vegetative and reproductive growth stages (Fig. 6 C and D). Taken together, the results presented here suggest *Ss*CUC2 has a conserved role in boundary formation and early SAM formation.

## Discussion

Plant-specific CUC TFs are required for several developmental progressions, such as establishing boundaries, the emergence of leaf primordia from apical meristem, floral organ separation, etc. [1, 10, 15, 47]. However, the *CUC* genes have not been functionally characterized in monocots except in rice [48].

In this study, the *CUC* genes from sugarcane were studied in detail. The identified *SsCUC* genes were distributed on Chromosomes 7 A, 7 C, 7D (for *SsCUC2*), 6 A and 6B (for *SsCUC3*). There is enough evidence to suggest that exon-intron structure variation is critical for the evolution of gene families. The gain and loss of exon-introns are caused by the reorganization and combination of different chromosome fragments [49]. The exon-intron organization study of the sugarcane *CUC* genes and *CUC* genes for 5 monocots and 6 eudicots indicated that *CUC* genes are intron poor. Generally, the gene belonging to the same group shared similar intron-exon patterns with few exceptions (Fig. 2).

The similarities and differences among gene family proteins could be reflected in the composition of motifs [50]. The study of motifs present in CUC2 and CUC3 protein from different monocot and eudicot species revealed that most CUC2 proteins possess additional motifs compared to CUC3 (Fig. 1). Also, proper protein folding, activity and secretion of many proteins require N-glycosylation, a common post-translational modification of proteins in eukaryotes. N-linked glycans are processed in the ER by α glucosidases I and II and modified in the Golgi apparatus into complex structures [51–53]. Interestingly, among the five identified CUC sequences of sugarcane, only SsCUC3 sequences possessed an N-glycosylation site at the C-terminal region. Also, there were extra protein phosphorylation sites in SsCUC2 compared to SsCUC3 in the NAM domain and in the N-terminal region (Table 1, Additional file 1). Both the N-linked glycosylation and protein phosphorylation play a crucial role in the activity of the protein. These differences between SsCUC2 and SsCUC3 could be the origin of the difference between their function and regulation.

Previous investigations of the evolutionary origin of CUC proteins suggest that the diversification of CUC3 from the CUC1/ CUC2 occurred more than 150 million years ago [54, 55]. Hasson et al. (2011) suggested that CUC2 may have preserved much of the inherited function after duplication of an ancestral gene, whereas CUC1 may have diverged, with changes affecting both the regulatory and coding regions of the gene [10]. We reconstructed the CUC phylogeny to investigate the evolutionary differences between the sugarcane CUC proteins and selected monocots and eudicot CUC proteins. Consistent with previous reports, the eudicot CUC formed two different clades, one with CUC1/CUC2 and another with CUC3. The monocot and eudicot sequences were also settled separately in the phylogeny (Fig. 3).

The ovule in seed plants forms the female gametophyte, which is responsible for fertilization and seed development. The primordia of the ovule comprise three separate regions. In *Arabidopsis*, CUCs have been linked to ovule initiation, ovule number, and ovule individualization [43, 44, 56, 57]. Previously, *CUC* transcripts were detected at the boundaries between the chalazal region, nucellus, and ovules [20]. In the RNA-seq of sugarcane, we also noticed the differential expression of *CUCs* during different developmental stages of the ovule. During the initial stages of ovule development, the expression of *SsCUC* genes increased several folds and gradually decreased, returning to the basal levels at ovule maturation (Fig. 5 A and D). These observations indicate that *SsCUC* genes participate in ovule development, which is in agreement with the previously reported functions of the *CUC* gene [20, 43, 56, 57].

In general, miRNAs function as negative regulators of the genes that act post-transcriptionally [58, 59]. Several NAC genes are also targeted by miRNAs that regulate their expression, including NAM proteins of many gymnosperms that possess characteristic miR164 binding sites [19]. MIR164 regulates floral organ number and boundary development by creating and controlling the boundary domain by post-transcriptionally regulating *CUC1* and *CUC2* [35, 45]. In Arabidopsis, miR164-CUC2 module activity in the meristem tightly regulates plant development [36, 60, 61]. Mutations in the OsNAM, rice orthologous gene of CUC1 and CUC2, display a small phenotype with fused leaf structure, small panicles, and defective floral organs [48]. Also, overexpression of OsmiR164b displays a phenotype similar to OsNAM, and the OsmiR164-resistant version of *OsNAM* shows altered expression in the meristem, indicating that the *OsNAM* expression is regulated by OsmiR164 [36]. We also identified the miR164 binding site in sugarcane SsCUC2 using bioinformatics and PARE-seq data. Our results show that *SsCUC2* possesses a miR164a binding site suggesting that the expression of sugarcane *CUC2* is also regulated by miR164a (Fig. 5B C).

Since CUCs have redundant functions in *Arabidopsis*, a single mutation does not induce an extreme phenotype, but the double mutation prevents the development of embryonic shoot meristems (Additional File S3) [1, 19]. We verified the conserved function of the *SsCUC2* gene by complementing the *Arabidopsis* *cuc2cuc3* mutant phenotype using *SsCUC2*. The complemented plants rescued the cup phenotype of *cuc2cuc3* mutant and distinctive SAM growth and floral architecture (Fig. 6 C & D), suggesting that SsCUC2 has similar biological functions to AtCUC2.

## Conclusions

Here we studied *CUC* genes in the sugarcane and explored their regulation during sugarcane ovule development. We also identified the conserved function of SsCUC2 in boundary formation and SAM development by ectopically expressing SsCUC2 in the *Arabidopsis cuc2cuc3* mutant. Taken together, this study provides essential information about the conserved functions of the SsCUC2, especially in boundary formation and in ovule development, which is a critical trait in crop breeding.

## Methods

### Plant materials and growth condition and treatments

Sugarcane (*Saccharum officinarum* L.) cultivar Yuetang 91-976 samples were collected from State Key Laboratory for Conservation and Utilization of Subtropical Agro-Bioresources, Guangxi, Nanning, China. DIC observation of the different stages of developing ovule [Archesporial cell (AC), MMC, meiosis, mitosis, and mature] was carried out to establish the developing stages in the sugarcane inflorescence, followed by sample collection for each stage using micro-dissection needles. All samples were snap-frozen in liquid nitrogen and stored in a deep freezer at -80 °C for subsequent RNA extraction.

The *Arabidopsis thaliana* (Col-0; CS60000) was used as the wild-type, and all plants used were in Columbia background throughout this study. T-DNA mutants of *cuc2-3, cuc3-105* and *cuc2-3cuc3-105* were kindly provided by Dr. Nicolas Arnaud (INRA-AgroParisTech, France). Surface sterilized *Arabidopsis* seeds were placed in round, 90 mm Petri-plates on the modified Hoagland’s medium containing 1% (w/v) sucrose and 1% (w/v) agar as described previously [59]. The plates were kept at 4 °C in the dark for 2 days for seed stratification. After stratification, the plates were transferred to the growth room at 22 °C with a 16 h light/8 h dark photoperiod under an intensity of 100 µmol m^−2^ s^−1,^ and seedlings were grown vertically [62]. For *Arabidopsis* transformation, *Agrobacterium*-mediated floral dip method was performed [63], and transgenic plants were selected on media plates containing 50 mg l^−1^ hygromycin.

### Identification of CUC genes

The sugarcane *(S. spontaneum)* AP85–441 genome sequence data was downloaded from http://www.life.illinois.edu/ming/downloads/Spontaneum_genome [41]. We searched CUC genes from the *Saccharum* genome using BLAST-P with the e-value set 0.01 with *Arabidopsis CUC* genes as the query. The identified sequences were further verified, and redundant sequences were removed. Besides, the *CUC* genes from 5 monocots (*Ananas comosus*, *Oryza sativa*, *Brachypodium distachyon*, *Hordeum vulgare* and *Zea mays*) and nine eudicots (*Arabidopsis thaliana*, *Brassica rapa*, *Daucus carota*, *Solanum lycopersicum*, *Eucalyptus grandis*, *Citrus sinensis*, *Gossypium Raimondi*, *Glycine max*, and *Phaseolus vulgaris*) were obtained from Phytozome V12.1 (https://phytozome.jgi.doe.gov) and NCBI (Additional file S2).

### Phylogenetic analysis

The phylogenetic relationship of CUCs was studied using peptide sequences retrieved from monocots and eudicot plants listed above. The multiple sequence alignments were performed using MUSCLE, and the phylogenetic tree was constructed by CLC Genomics Workbench v12.0 (CLC Bio, Aarhus, Denmark) using the Neighbor-Joining method with default parameters and the bootstrap test of1000 replicates.

### Gene structure analysis and conserved Motif Identification

The schematic *CUC*s structures were drawn by the Gene Structure Display Server 2.0 (http://gsds.gao-lab.org) [64]. The conserved motifs in the CUCs were identified by MEME (Multiple Em for Motif Elicitation) server 5.3.3 (https://meme-suite.org/meme) using default parameters with the maximum number of motifs set at 10, and the optimal width of each motif was set between 6 and 100 residues.

### RNA-Seq, small RNA, and PARE-seq analysis

RNA was extracted from the samples collected at different developmental stages of the ovule (AC, MMC, meiosis, mitosis, and mature) of the sugarcane. Ten mRNA-seq libraries, 10 small RNA-seq libraries (two replicate for each stage of developing ovule) and 5 degradome libraries were constructed (one replicate for each stage of ovule). Library construction and high-throughput RNA-seq, sRNA-seq, and PARE-seq were performed by LC Sciences (Zhejiang, China). After sequencing, the raw reads were filtered, and adapter sequences were removed along with contamination and low-quality reads from raw reads. The remaining unique sequences (clean reads) were then processed for further analysis. The transcript abundance of sugarcane *CUC* genes and miR164 was calculated as per million reads (RPM) method using CLC Genomics Workbench v12.0 (CLC Bio, Aarhus, Denmark). The heatmap was generated using TBtools after converting the expression values in fold change (additional file S4).

### RT-qPCR and expression analysis

Total RNA was extracted using the RNeasy kit (Qiagen, MD, USA), followed by DNase I (Thermo Fisher Scientific, CA, USA) treatment. First-strand cDNA synthesis was carried out using 1ug of total RNA using ThermoScript RT-PCR kit (Thermo Fisher Scientific, CA, USA). In a CFX96 qPCR system (Bio-Rad, Singapore), quantitative PCR was performed with FastStart DNA Master SYBR Green I master mix (Takara, Shiga, Japan). Using 2^−ΔΔCT^ method fold change in the expression was determined using the *β-actin* gene as the internal control. For miRNA, stem-loop qPCR was performed the reaction cycles were the same as with mRNA analysis. Three biological replicates and at least three separate technical replicates were used for each stage. Additional file S5 contains a list of the primers used in this study.

### Vector constructs

The *Ss*CUC2-GFP was generated by amplifying the coding sequence of *Ss*CUC2 (Sspon.07G0020380-1 A) without the stop codon from sugarcane leaf cDNA using the primers listed in additional file S5. The amplified PCR fragment was then cloned into the pENTR/D-TOPO vector (Invitrogen). pENTR/D-TOPO clones were then recombined into the destination vector pGWB505 using LR Clonase II (Invitrogen), and the construct was confirmed by sequencing.

### Transcriptional activation analysis in yeast cells

The *Ss*CUC2 ORF was cloned into pGBKT7 to generate pGBKT7-*Ss*CUC2 using the primers listed in additional file S5. The yeast strain AH109 was then transformed with pGBKT7, pGBKT7-53 + pGADT7-T, and pGBKT7-*Ss*CUC2. The transformed yeast cells were grown on SD (-Trp), SD (-Trp/-His/-Ade), and SD (-Trp/-His/-Ade/α-gal). The growth status and α-gal activity discovered the transactivation activity of *Ss*CUC.

## Supplementary Information

**Additional file 1. **Schematic diagram representing the NAM domain, N-glycosylation sites and phosphorylation sites in SsCUC proteins. Red arrows represent the NAM domain, yellow arrowheads represent N-glycosylation sites and pink arrowheads represent phosphorylation sites.

**Additional file 2. **List of sequences used in the present study.

**Additional file 3. **Phenotype of CUC single mutants (*cuc2, cuc3*) and double mutant (*cuc2cuc3*).

**Additional file 4. **SsCUC expression and regulation. (A) A- Expression of CUC genes in RNA-seq, sRNA-seq and PARE-seq data of sugarcane during different stages of ovule development. (B) Normalized expression of miR164a in sRNA-seq data of sugarcane during different stages of ovule development. (C) Target identification of sugarcane CUC2 gene using degradome sequencing.

**Additional file 5. **List of primers used in the present study.

## Data Availability

The sequencing data that support the findings of this study have been deposited in the NCBI SRA database with BioProject accession no. PRJNA723681, which will be available publicly upon acceptance of the article. All the protein and DNA sequences analyzed during this study are included in this article as Additional file S2. The expression values used to generate Fig. 5 A are provided in Additional file S4.

## Abbreviations

NAM: NO APICAL MERISTEM
ATAF: Arabidopsis transcription activation factor
CUC: CUP-SHAPED COTYLEDON
CUP: CUPULIFORMIS
