# Supplementary figures and images for: Interspecific complementation-restoration of phenotype in Arabidopsis cuc2cuc3 mutant by sugarcane CUC2 gene
Source: BMC Plant Biol. 2022 Jan 22;22:47. doi: 10.1186/s12870-022-03440-z (PMC8783490; doi:10.1186/s12870-022-03440-z)

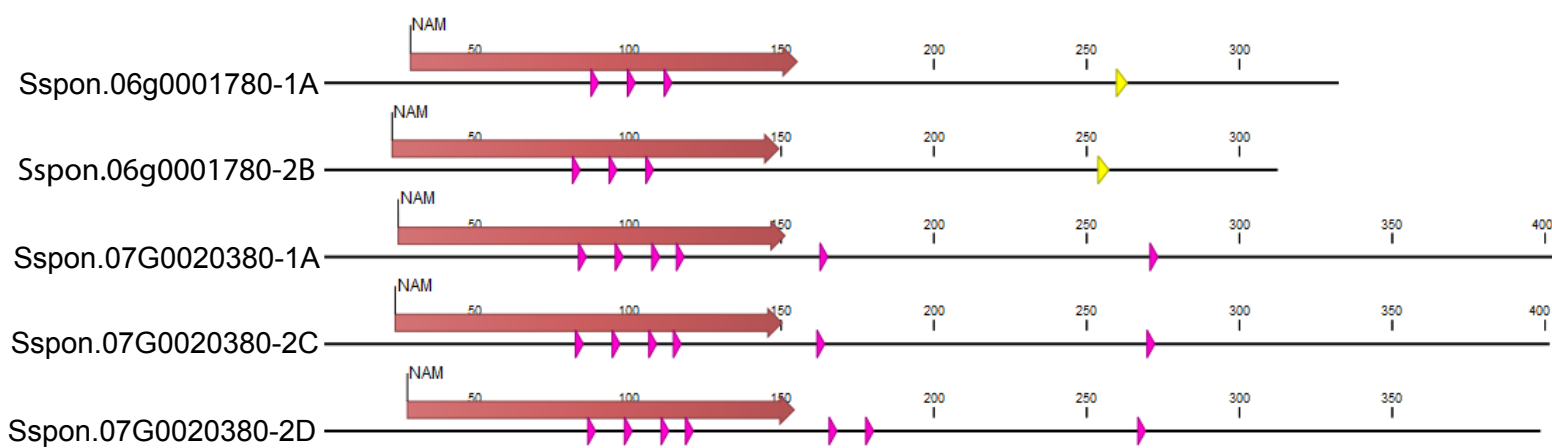

► N-glycosylation sites    ► Phosphorylation sites

Supplement: Supplementary file 1 — Additional file 1. Schematic diagram representing the NAM domain, N-glycosylation sites and phosphorylation sites in SsCUC proteins. Red arrows represent the NAM domain, yellow arrowheads represent N-glycosylation sites and pink arrowheads represent phosphorylation sites. [file 12870_2022_3440_MOESM1_ESM.pdf]

Col-0

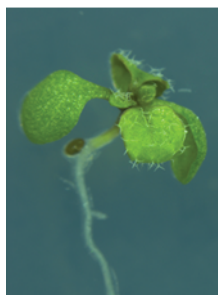

*cuc2-3*

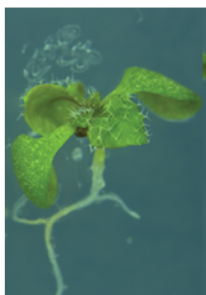

*cuc3-105*

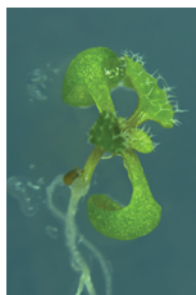

*cuc2-3cuc3-105*

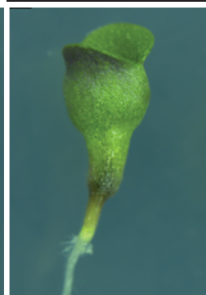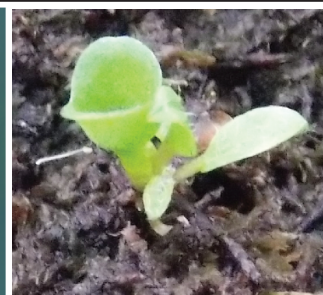

Supplement: Supplementary file 3 — Additional file 3. Phenotype of CUC single mutants (cuc2, cuc3) and double mutant (cuc2cuc3). [file 12870_2022_3440_MOESM3_ESM.pdf]
