# Supplementary material for: Interspecific complementation-restoration of phenotype in Arabidopsis cuc2cuc3 mutant by sugarcane CUC2 gene
Source: BMC Plant Biol. 2022 Jan 22;22:47. doi: 10.1186/s12870-022-03440-z (PMC8783490; doi:10.1186/s12870-022-03440-z)
Supplement: Supplementary file 2 — Additional file 2. List of sequences used in the present study. [file 12870_2022_3440_MOESM2_ESM.docx]

Additional File S2: List of sequences used in the present study.

**A) Genomic sequences**

>*At*CUC1 |AT3G15170|*Arabidopsis thaliana*

AACAGGAGAAACACAGATCATTAAAATAAAGGACCTTTTTTATTTTCCTCTTCTTTTCTCTTCCTTTATCTTTCAACATTTGGGAACTCTAGGGTTTTGTTATTTTTAGAGCTTTCTTCTTGTGCCGACAATGGATGTTGATGTGTTTAACGGTTGGGGGAGGCCAAGATTTGAAGATGAATCCCTTATGCCACCTGGGTTTAGGTTTCATCCAACTGATGAAGAGCTGATCACTTACTATCTCCTCAAGAAGGTTCTTGACTCTAATTTCTCTTGTGCCGCCATTTCTCAAGTTGATCTCAACAAGTCTGAGCCTTGGGAGCTTCCTGGTAAGCCCACTTAACTCAAGTCAACCCACCTTCTGTTTCACAGGATGCACTCAAAAACTTTTACAAAACTTGATAATAAGTCCCATCTCTAGATTTTTGCTTAAATCGTCATAAACCCTAGTTTCATCAAGGCCAACTTATTAGACTTTCCTCTGTTCTTATTGCCATATCCAAATTCTAGGACTTTATTTCTGTGATAATGTGCTAGATGAGTAGATGTTAATGTAGCATGATATAGGCTTAGTGGAGACACTGTTAGAACTCTTTAATTAAACTTTCATTAGTTTTGAAACTAAACCAGTCCTTGTTCGGTTCTGAAACAGAGTCTCTACGTAGTTATAAATCTTGCTAACGACTTTGTTATAAATTATTAATATTTGATCAAGTTTTAAAGAAATCAAACTCTTGTTCAACTTTATATCAACCCTTCACATGAGTGACCAGTCCTTGTTCGGTTCTAAAACAGAGTCCTCCTTACTCTGTTTGTAACTTTCACTTATACCATTTGAGCAGAGAAAGCGAAAATGGGGGAGAAGGAGTGGTACTTCTTCACACTAAGAGACCGTAAATACCCAACGGGACTGAGAACGAACAGAGCAACAGAAGCTGGTTACTGGAAAGCCACTGGTAAAGACAGAGAGATCAAAAGCTCAAAGACAAAATCACTTCTCGGGATGAAGAAAACTCTTGTCTTTTACAAAGGCAGAGCTCCTAAAGGAGAGAAGAGTTGTTGGGTCATGCATGAGTATCGCCTTGACGGCAAATTCTCTTACCATTACATTTCCTCCTCCGCTAAGGTAAAGTATTAAATCTCTATATATCAATCCACTTTTCTCATACATATTATAATCAAAATATGTTTTCTTTTACACTAGGATGAATGGGTTCTCTGTAAAGTTTGTCTGAAAAGCGGCGTAGTTAGTAGAGAGACGAACTTGATCTCTTCTTCTTCTTCTTCTGCCGTCACCGGAGAGTTCTCCTCTGCCGGTTCTGCAATTGCTCCGATCATCAATACCTTTGCGACGGAGCACGTGTCCTGTTTCTCCAATAACTCTGCTGCTCATACCGATGCGAGCTTTCATACATTCCTTCCCGCTCCACCGCCGTCACTGCCCCCACGTCAGCCACGTCACGTCGGTGATGGCGTGGCGTTTGGTCAGTTTCTGGATTTGGGATCATCGGGACAGATTGATTTCGATGCAGCAGCAGCAGCGTTCTTTCCGAATCTACCTTCTCTGCCTCCCACGGTTCTTCCTCCTCCTCCGTCATTTGCAATGTACGGTGGAGGCTCCCCCGCCGTGAGTGTGTGGCCGTTTACTCTCTGATCGCCATGCGGTCATTAAACTATCTTTACTATCTTTTAATCTAAGAGAAAAACTACCTTGGGTTGCTTAATATTAGTGGCTTTGTATTAGTCTCGAATCATTTCCTCGTAGCATTTTGCTGTTGTACGTGTTTGTAGTAACGTCTTTGTGCATGTTTTTAAACATGAACCTGC

>*Br*CUC1 | Brara.C03560.1|*Brassica rapa*

CGTCCTCTTTCGTGTCTTTTGTTCCTTCCCCACACAAATGGATATGGATGTGTTTAATGGTTGGGAGAGATCGAGATATGAAGATGAAACCGTAATGCCACCTGGTTTTAGGTTTCATCCTACCGATGAAGAGCTCATCACTTACTACCTCCTCAAGAAAGTCCTTGACTCCAGTTTCTCATGTGCCGCCATTTCTCAAGTTAATCTCAACAAGTCTGAGCCTTGGGAGCTTCCTGGTAAGCCCACTCAACACTGTTAAAACTCTAGTTTAATGTCATAAACCATCGGTATAGGTTCAAAGTTTGAGACTTTACTATGTTCTTGCATACCTTTAGACTTAAGACATTGTTATAACTCTACCAAATGTTATCAGAGTTAAACCCATCTGTAGAATTATGTCTAAAACTCTAGTAATAGGTTCAAAGTTTCAAACTTTCCTCTTTTTTTAGATACTTAAAGAGCCATTGGTCTAACCTCCCAAGCTTAGACTTTTCTCCTATCAATTTAAATGTCATATATGTATACAAGGAAGAGGGTCTAGGTCAATCATTATAACTCTTAACATTATGAAGTCCAACTTTTAATTAAAAACAAACACTACATGATCATCACCAGTCTAAGTTTCTCTGTTTTAAAACAGAGTATTTACTTACTTTGAGACTTTTCTTTCACATATGAGCAGAGAAAGCGAAGATGGGGGAGAAAGAGTGGTACTTTTTCACACTGAGAGACCGTAAGTACCCAACGGGCCTAAGAACTAACAGAGCAACAGAAGCTGGCTACTGGAAAGCAACTGGTAAAGACAGAGAGATCAAAAGCTCAAAGACAAACTCGCTTCTCGGGATGAAGAAGACTCTTGTCTTTTACAAAGGCAGAGCTCCTAAAGGTGAGAAAAGCTGTTGGGTCATGCATGAGTATCGCCTCGACGGAAAGTTCTCTTACCATTACATCACTTCCTCCGCTAAGGTAAATCTCGTAATCTTTCTATATGATTCATTCACTTACCCAAAAAAAAAAAAAGTAAACTACCAAAATGTGTTTTCGAAGTTAAACAATTAAAATGTGTTTTTATTATTTTTTAAAAAATTGTGGTTTTTATGTTTTGTTCTTATTACACAGGATGAATGGGTTCTCTCTAAAGTCTGTCTGAAAAGCAGTGTTGTCAGTAGAGAGACCAAACTGATCTCTTCTTCCGGCGGTGTCAACTGCTCCTCCTCCTCCTCCGCCGCTGGTTCGTTAATTGCTCCGATGATCGACGCCTATGCGACGGAGCACGTGTCCTGTTTCTCCAATACCTCTGCAGCTCATGCTGACGCGAGCTTTCCTCCTGCTTACCTTCCCGCTCCTCCTCCACCGTCTCTGCCACGTCAGCCTCGCTGCTTCGGTGATGACGTGGCGTTTGGTCAGTTTATGGATGTGGGAGCATCTGGACAGTTCAGCATCGACGCAGCGTTTTTACCGAATCTACCTTCTCTGCCTCCGACGGTGTTTACAACTCCTTCTCAGCCGTTCGGAATGTACGGTGGAGGCTCCGCCGTGAGTTCGTGGCCGTTTGCTCTCTGATGATCGTTATGCGGTCACAAGCTATCTCTAGCTATCTACCGTTTATGAATTCGTTTTAAGGAAAACTAGACTTTCGTTTTAATTAAATGGTTTGTATTAGTTTCGAATCATGTAGCTGTGTGTTGCATTTGGTTTATGTACGTG

>*Dc*CUC1 |DCAR_020996|*Daucus carota*

ATGGACATCTTTTACCACAATCTGCAGAGCAATGCAGATGCGCAACTGCCTCCTGGCTTCAGGTTTCACCCAACAGATGAAGAGCTCATCACTTATTACCTTCTCAACAAAGTTCTTGATCACAACTTCACTTGCAGAGCCATTGCTCAAGTTGACCTCAACAAATGCGAACCATGGCACCTCCCTGGTAACTAATCAACACTTCATTTTCTGCCAAGTTTCTCTTTTTTAGTTTTTTAATTCTCACAGCTTTGGCTTGATTTTGTGATGCCTTAACGCAGAGAGAGCGAAGATGGGGGAGAAAGACTGGTACTTTTATAGCTTGAGGGACCGGAAGTACCCGACAGGGTTGAGGACGAATCGAGCAACCGAAGCTGGATACTGGAAGGCCACGGGGAAAGACAGGGAGATTTACAACTCAAAGACATCGTCTCTGGTGGGCATGAAGAAGACTCTTGTCTTCTACAGAGGCCGTGCTCCCAAAGGAGAGAAGACCAATTGGGTGATGCATGAATATCGCCTTGATGGCAAGCTGGCTTACCACTATCTTTCCACTAATTCTAAGGTACATTCAATTCAAAACATGTCAATGTAATAAATCATCTAATTAGAATTAGTCATCTGTCTGTCGCGTTATTTGGGAAAAAAAAATTGATACAGATTTTGGATTGCTCATGTTTATATTGATGAATTATAATTGGCTAATTTTCAGGACGAGTGGGTGATATCCAGGCTGATGAAGAAAAGTGGTACTGCTGGCCCTGGTGACAAAACACCAACTTATGGCATGTACTCAGAAATGAGCTCTTCATCCTCCGCTTCTCTGCCACCTCTTCTAGACTCCACTCCCTTCACAGCAGCAACAACTGATCATCATCGCGTGATCGACTACTCCTACCTCGAGAAGGAGCACGTGTCCTGTTTCTCCAGATCCACAGCTGCACCTGGCGGCTTCAATTACCATACGACTCTATTTGACTGTGGCCTTCCTCCCCCGCTGATGGTCGACCACACCTCCTCCTCAACGTCATCCTCGTCTCAATTCCACGAAAACAATACAAATGGAGGGGAGAAGCTTCACTTGCCTAGCTTCTTTTTCCCCTCCATCACCCCGTCTCCGATTCATGGAGGCGTAGGAAGCATTTATGCATCTGACACGGGGAATTATTCAGTGCTGGAGGCTCAAAAGCCTGGCCTCACTGAGCTTGATTGCATATGGAGGGGCTCTTTTAATTGA

>*Sl*CUC1 |Solyc06g069710.2.1|*Solanum lycopersicum*

ATGGAGAATTATTCAGGAGTTGTTAAGGATGATGATCAGATGGAGTTACCACCTGGATTTCGATTTCATCCAACTGATGAAGAATTGATCACTCATTATTTGTCTAACAAAGTTGTGGATACTAATTTCGTTGCTATTGCTATTGGTGATGTTGATTTGAACAAAGTTGAACCTTGGGACCTTCCATGTAAGGTTTTTTTTTTTTAACGAAGCGAGATTCATATAGCCGACCTCAGCTTGATTTTTTGTTGTTGTGTTTGAATGTAGGGAAGGCGAAAATGGGGGAAAAAGAATGGTATTTTTTCTGTGTGAGAGACAAGAAGTATCCAACAGGGCTGAGAACAAACAGGGCAACTGCTGCAGGGTATTGGAAAGCTACTGGAAAAGACAGAGAGATTTTCAGGGGAAAATCATTGGTTGGTATGAAGAAAACTCTGGTTTTCTACAAAGGGAGAGCTCCAAAAGGTGAAAAGACAAATTGGGTTATTCATGAATTTAGATTAGAAGGAAAATTGTCTCTTCAAAATCTGCCAAAGACAGCAAAGGTACACATAATCTAACATTCAAGCAGATAAGGTCTGTGTACATTCTGTTCTCTATATGTTTATCGTTGTGTTTTTAATCAAGATTCCTTTTTTTTTTCTTCTTTTTTTGTAGAATGAATGGGTGATTTGCAGAGTGTTTCAAAAGAGCAGTGGTGGAAAGAAAATCCACATTTCAGGGCTTTTGAAACTGAATTCTAATGAAAATGAAATGGGGAATTCATTTCTGCCACCATTGACAGATTCTGCTACTGCTACTGCTTCGAAATCCAGCCACGTGCACTGCTTCTCCAATTTTCTCACTGCTCAAAACAACTGTTTCCCTCTTCTGTCAAATCCAATGGATAGTTACCCTACAACTTCTCTTGTTCCAAATACATTTTCTTGTAACCAAATAGCTCCATTCACTACTACTAATAATCCAGCTTCATTTGGGGTTCAAGATCCTTCAATTCTTCTAAGGACTTCACTTGACAGCTATGGTCTGAATTTCAAGAAAGAGGACATTTTTAATGTACCCCAAGAAACAGGGGTAATTAGCACTGACATGAATACTGATATCACCTCAGTCGTATCAAATCTTGAAATGAAAAGAAGGTTTCTTGAAGATCAGGTGCCATCAGCAGGTATGGTTGGATTACAGGGTCTTGATTGTCTCTGGAGTTGCTGAATTATCTTGAAACAAGAATGCCAAATAAGTCAAAAAACCCAA

>*Eg*CUC1 |Eucgr.F01170|*Eucalyptus grandis*

ATGGAGAACTACCACCAGTACAGCAACCACCACCTCGTCAATGGCGACGGGCATTTGCCTCCGGGGTTCCGGTTCCACCCCACCGACGAGGAGCTCATCACGTATTACCTGCTTAAGAAGGTCCTGGACAGCAGCTTCACCGGGCGAGCCATCGCCGAGGTCGACCTCAACAAGTGCGAGCCCTGGGAGCTTCCCGGTAAGACAATGCTCCGCAGGACGCCAACCAGCGGTGGCGCTTCTTTTCGAACGAGTGTTCCTGACACTGTCTCTTGCAGAGAAGGCGAAGATGGGGGAGAAGGAGTGGTACTTCTTCAGCCTGCGGGACCGTAAGTACCCGACGGGGCTCCGCACGAACCGGGCGACGGAGGCGGGGTACTGGAAGGCGACGGGGAAGGACCGTGAGATCTACAGCGGCAAGACGGGGTCGCTGGTGGGTATGAAGAAGACCCTCGTCTTCTACCGGGGACGAGCCCCGAAGGGCGAGAAGAGCAACTGGGTCATGCACGAGTTCCGCCTCGACGGCAAGTTCGCCTACCCCTTCCTCTCCCGCTCCTCCAAGGTATTATACTAGTTTTCACTTCCAACGTCGTCTCATCGCATCGTTTTGTCGGGGGCATTTTGGTCTCTCTCTCTCCCTCCCCTTTCTCTGTCTCCGAGGTGTGTCTTGATTATGCTCTTCGAGTGGTTTTTCTCTGCGGGTTTAATGCGTCAGTGATAATGTTACTCGCATTCTTCGCTGCGCTTGGAAGCTTTCTCGAGCCCTGTTACCTTTGCCTTTTAACTGCTTTCATGAAGACGTCGCAGATGCTCAGTGTATGGAACAGCCAGTTCGTATGCATGGGAGAGTTCAGTCGAGTTAAGTCTCGGGCCGTTAAAAAGGGTTTTAAGTGACTTTGTTGTTTTGTTGTCGCCGGAAGTTGACCTCTTGACCCGCTCATTTCATTTCCGTTTTGAAGTTTAACGTGTCGAAGTGTCATTCAGATTTTCCTGCATTTATTTGGAGTTACGTATCCTCTCGGGGTTTCAGGATTTTTTCCTCTTTTTCTGACAGTGAGTTTCTCATTCTTAAAATTATAAACTTAGTGATTATATTCACTTGTGAAGACGCCGAGTAGTCATTTCCACGACAGTTTCTATCGATTTGGTACTTCTAAACTGCATTGATAAGTTAGTGTAAGTTGGGGGTCATGTCAGGGAACAGCTTTGTAGGACGACTTAATTGATTAATTTTGGTCACCGCAAGTTGTTTTGAGTTACTGGTTTCTAATTGTTAGAGGTGTTAAAGATGTTTCATATACGGGTTAGTGCTAGGACTATACGCCGAGCACACTTCCGTTGGATTTCCTCGAAACCCTAGGTTGATCAGAGTGTTATTACTACTAGTGGCTAGTTCCGAAGAGTGACTATTGCATTTATTGTACGTGTGCCACGGCACGAAATGGTATCCGATGCATTTCTGAAAAGCATTGTCTTTTTTCTCTATGTAAACGGTTTTGGCTTTGCTTTGCAGGATGAGTGGGTGATCTCCCGCGTTTTCCAGAAGTCCAGCAACAGCTGCGGCGCAGCCGCCCCTTGTGGTGGCAAGAAGACCCGCATGCCCCCCCACATGAACCTGTACCCGGAAATCGGTTCACCCTCGGTGTCTCTCCCACCACTGCTCGACTCCTCTCCTTACACCTCAACCACCGCGGGGTTCATCGACCGCGTCCCCATCTCTTACGACAGCTCAATCCCTAAGGAGCACGTGTCCTGTTTCTCCACGGCCGCTGCCGCCTCGTTGGCAACCCACAATTTCGGCAGCAACCCTAGTTTCCAACTGGCTCCTCCCACGGCCCCCCTGATCAATGCAACTGACCCGATGACCCGATTCTCCAGGAGCATCGGCGTCTCGGCTTTTCCGAGCCTGAGGTCCTTGCAGGAGAATCTCCAGCTGCCTTACTTCTTCTCCGGTAACCAGCATCTGAGTGGTGGAATTAATGATTTGGTCAGCTCGACTTCGAGCTCTCTGGGGAACTGGACGGCTCCGGATGATCAGAAGGCGGTGGACCTCGGTGGTCGAATGGGAATGGGCTCGTCGGAGCTTGACTGCATGTGGAACTTCTGA

>*Cs*CUC1 |orange1.1g017827m|*Citrus sinensis*

ATCCTACTCATCTTTCTATCTTATCTCTCTTAGCTTTATGTATTAAGCCGCCTCTCTCTTTGTTCTCTCAATCATCTATATTTTTCATTTAGAGTTTCAGACAAGTAAACAAACAGGGTACTAGCTAATTGAATTGAAGGGTTATGGAAAACGTTTCTGCAGTTGGAAAGGAAGATGACCAGATGGATTTGCCGCCTGGTTTCAGATTCCATCCAACTGACGAAGAGCTTATCACTCACTATTTGTACAAGAAAGTTCTTGATGTTTGCTTCTCTTGTAGAGCTATTGGAGATGTTGATCTGAACAAAAATGAACCTTGGGAATTGCCTTGTAGGTTCTCTAATTTTTTTTTTTATGAAATTTGCATTTGAGTTTTTTTATTTCAGTCTTGAAGATCTAATCTTACCTTGTTTGTTTGTCTTCTTTTCTTGCGGTTGTTTGGATGTAGGGAAAGCAAAGATGGGAGAGAAAGAATGGTACTTTTTCTGCATGAGGGATAGGAAATATCCAACTGGTTTGAGGACTAACAGGGCGACTGTATCTGGTTATTGGAAAGCCACGGGGAAAGACAAGGAGATTTACAGAGGAAAATCTCTAGTTGGAATGAAAAAGACTCTTGTTTTTTACAGGGGAAGAGCCCCAAAAGGGGAGAAATCAAGCTGGGTCATGCACGAATACAGATTGGACGGCAAATTCTCTGTTCATAGTCTCCCCAAAACTGCCAAAGTTTGTGCCTTTTCCGCTGTTTTTCCCCTGTTTTTTTCCTCTGTTTTTGTCAACTCTGTTTTTGTTAACTCTGTTTTTGTGTTTTCTTGTTATGCAGAATGAGTGGGTGCTTTGCCGGGTGTTTCAGAAGGGTTCTGGTGGGAAAAGGACTCATATTTCAGGGCTAGCGGGATTAGGGTCTTTTGGAAATGAATTGGGTCCTCCTGGCTTGCCACCATTAATGGATTCATCTCCTGATAATGGCAGCAAGACCATCAAATCTGTTGCCGATTCGGCTTACGTGTCCTGCTTCTCCAATTCTATTGATCTTCAAAGAAATCAAAAAACCACCACCACCATTGAAAATTTTTTCAACAATCCTCCTCCCATCTCTGTGTCTTCAAACTGTCCTGATGTTTTTCCAAGAATCCCACTCTCTTCAAACTCATTCTATTCTCCTTTATCGGTCCCGGTTCCATCACACGCGCAATTCCCAGGCTCTGTTTTCATGCAAGACCACTCAATCTTAAGGGCCTTAATTGAAAACCAAGGATCAAACATGAGCCAGAGTTTCAAAACAGAAAGGGAAATGATCAGTGTTTCACAAGACACAGGCCTCACTGCTGACATGAACCCTGAAATCTCCTCAGTTGTGTCCAACCTTGAAATGGTGAAGAGGCCATTTAATGATCATGATGCTCCTTCAACTTCAGCTGGACCAGTGGATTTTGACTGTTTTTGGAATTACTGACAGTAAAATCAAATTAAAAAAAAAAAGAAAAAAAGAAGCCTATTATTATCTACTATCGTTTATCTTGATAAAGTGATAGTGTGGGTCTGTCTGTGTATAGATTTTCTGAATCTAAATTGAAGGATTGAAAGAAATTAAATGTAGTAAAAGAACATATATTGAGTGTTGTATGAAAGTATGATTATGAACTGGGTTTGTTTTCTTGTCAATGTTTATTTAGGGGTTTCTTCCAATATTGGCATCATTAATTCATTATTGTCAAATTGCTTGGCTAGGAAGTTTGTCATATATGAGATTCAGTTGGTGGCTAACTGATCTTATTTTGTATCTCTCTCAAAAAATTATTGCATTTTAGCCTGTGGCATGCTTACTGCTTACATCATTGAAAGCATGAGATTTATTCTTGTGAATTCTTAAATCTTCAGATTTGGATTTTGAATATCAGCTTGTGGGGACTCTGCTTAGTGATATAATTTCAG

>*Gr*CUC1 |Gorai.007G323900|*Gossypium Raimondi*

GTTCCCATTTCCCTCTCTTTGGAAAAAGAAGAGAAAGATTTCGGAAGAGGACGGTACAGAGCAAAAACACAGATTTTTTTTTTCAATATATATTCATTATTTTGCCTTTTTTCCTTGTCACCGGATTCATCAAATCCCTCCTTGACACCCTTTCTTCTCCATTCCTCATCAAATGAAAACCCTTTTCTCTCTTCACTTCATGATGTTGAACTTTGGTTTTTGAAGTCTAGGGTTTTTTGTCTGGTTTTCAAGCCAAGGGTTTTGTTTGATTTTTGGTTTTTGGGTATGGATAGTTACTATCATTTTGACAACGGTGATACTCATTTACCACCTGGCTTTCGTTTCCATCCAACTGATGAAGAGCTCATTACTTACTATCTCTTAAAGAAAGTTCTTGATAGTGGTTTTGCTGGTAGAGCTATAGCTGAAGTTGATCTTAACAAGTCTGAACCTTGGGAACTTCCTGGTAAGTGATCATTTGAACATATTTTACGACCTACCCTTTTCTTTTTGTAATTTGGGTTTGTGTGATTTGGTTATACAATGGTTCAGGGAAGGCAAAGATGGGAGAGAAAGAGTGGTATTTCTTCAGTTTGAGAGATAGGAAGTACCCAACTGGGTTAAGAACTAACCGAGCTACTGAAGCTGGTTATTGGAAAGCTACTGGTAAAGATAGAGAGATTTACAGTTCAAAGACTTGTGCACTTGTTGGCATGAAGAAAACCCTTGTTTTTTATAGAGGAAGAGCCCCTAAAGGTGTAAAAAGCAATTGGGTCATGCATGAATATCGTCTTGAAGGCAAATTTGCTTACCATTATCTCTCCAGAAGCTCGAAGGTATGAGGTCTAAACATGTTGATCATTTTGAAGATTTTCATGAAAATTAACACTGAACAATGTTTTCTTGTTCCCAAAGCTGAAGTATCTTTGAATTCTGGTCTGTAGTGTAAGTGCATAGTGTTCATGATTTGGCTTAAAAAATATACAGAAGTATTATTGTTTCCGTACGGTGTTTTCTTCATTTAAAGAGATGGAAAAGGTGTGTTGGGGAAATCTTAATAATATAGAAAAGCTTCTGTCTCTTTTTGGGATTTAAATACCCACAACTTCGTCATTATTATCCCCATAAAGCAATTATACTATCTTCTGGGAAATTATTAATTCCATGTCTCTTGCACTTTTTCCAATGGTTATGCAATTCCCATTTCTAATTTAGTGCTGAAACTCCTTGTGGGATTGATAATTGTATTTGGGTTTCATTAATATTCATTTGATGTTGGATTTTCAAGACCAATAATATGATTTCTTAAATACTACTTTCTCATTCTCCTTTGTTTCATTGTTATATTGACCACCAGTTGCTTGGATGAAGAGTAATCATAATTTACAAAATGCTAAACGTTTCTATTATTCCAGGATGAATGGGTGATATCCAGGGTGTTTCAGAAGAGTGGCTCCGCCAATGGTGCCACCAGCAGCACAGGCCGAGGAGCCAAGAAGACCCGGATGAACGCCTCCATTGCCGTCTATCAAGAGCCAAGCTCTCCTTCATCGATTTCCCTTCCACCTCTCTTAGATCCCACCGCAACTGCCTTTGCCACCACTGATCACGACAGCTTCTCTTACGACAATTATGTTCAATCCGAGCACGTGTCCTGTTTCTCCACCGTTACTGCTGCTACAGCCGCCTCTGCCACTGCCACCACCACAACCGCCCCATCGGCGTTCCACCCTGGTTTCGACAAAGCATTTCCACCTCCACCCCAAATGATCAACACCACTTTTGATCCCCTCGCGAAGTACTCAAGAAATGTGGGTGCTTCCGTTTTTCCAACCCTGAGGTCACTTGAGGAGAATTTGCAGCTCCCTCTCTTTTTCTCCCAGCCAACAATTGAAGCACCAACGCTTCACGGTGGCTCATCTGTCAACTGGGGAGCTTTTTCCGAAGAAATTAACGATGGGTCTGTTGGTGGTAACAAGATATCAATTGGTCCAACTGAGCTTGATTGCATGTGGACTTACTAAACTACTAAACTTAAAGAGGAATTTCCACCATTATTGGTGCTTGGAATTTTCTCTTATCATTATCTCTAATTATGTTGTCACTAGGAGTAGGCATGGAAAAGGACTACTGAGATTTTCA

>*Gm*CUC1 |Glyma.12G226500|*Glycine max*

TTTCTTACAATTTCTTCACTGACAAAGGAATACCTGCACATTTCCTCACGCACGCACGCATACTCAATCGCCACTCCCTCGTACATCACCCTCTCTCTCTCTCTTCTCTCTCCTCGTTCTCTCTCTAGCTATGCGGATCATGATGAGAACCCTGTCAGAGAGTAGAGACAGCTATCAGAAGAGGATAGTGCAGAGAAAACACAGATTTTTCCAGCCATATCATACCTTATTATTTCACCTTTTTCTCTCTGCCACCCGATTAATCAAATCCCCATGAGACCTCACCACCCCTTATAAACCACACCCTCTTCTCTAATCCCAAACCAAACCAAACCCCACCAAAGTGAAATTAGAAAAAAATAGAGTGACAAACCCAAGTTATAAAACCTTCTTTCACCATTTTGCTCCTCTTGTTTGTTCCCCTTCCCCATGCCATGACTCTTATTTCCTCCTATATAAAAGCCATGAAGTAGTGTTATTAGTATATCCAATATTCTCCTTTTGCTTAAAAATTAAATTCTCCCTCACTTTTCATTTTCACCTCTCTTTTTCAAGTGTTCCTTTCCTCTTTTTCTCTCCAACGGACAACTCTTCTTACCACACATGGACCACACCGAAGCTCACTTGCCACCTGGTTTTCGGTTCCACCCAACTGATGAGGAGCTCATAACTTACTACCTTCTCAAGAAGGTTCTTGACAGCACCTTCACTGGTAGAGCCATAGCTGAAGTTGATCTCAACAAGAGTGAGCCATGGGAGCTCCCTGGTAAAAACACTTAACACCCTTTCTTTCTTTCTTTAGCTATTAATATTTTCATTCATGGCTTTCTATAGTGGCAGTAAAAAGAGTTTGGACTTTCCACCTCACCTAACCTTTGTTTTTGGCAGAGAAAGCTAAGATGGGAGAGAAAGAGTGGTACTTCTTCAGCTTACGTGACAGAAAGTACCCAACTGGGTTAAGAACCAATAGGGCTACTGAAGCTGGGTACTGGAAAGCCACAGGGAAAGATAGAGAGATTTACAGCTCCAAAACTTGTTCTTTGGTGGGGATGAAGAAAACCTTGGTTTTCTACCGTGGTAGAGCTCCAAAGGGAGAGAAAAGCAACTGGGTCATGCATGAGTATCGCCTTGAAGGCAAATTTGCTTACCACTACCTTTCTCGCAACTCCGAGGTTAGTATCATTTTCTTTTTGTTCCTTTATTCATTCGTCATTCTACATGTTCTATGTTGTGTTACTTTTTCTCTACTCATGTAGCATTGAACTATGTGTACTTTTTAACCCATGATGAGCCGTTTTTATTTTAACACCCCCCAAATTTGCAGTGAAATAACTCTAGTTGGGTAGGAATCTGCTTTCCCTTTTAGCTTTCCATAAGTAGGTTTCATTTACCAATGTTGAGATGATTTTTCCCTTTCGTTATCTTGTTTCCAAGTTTTTGTTTCTCTTTTAAGATTTTGTTCCCCTTCCATAAGTAACCTTTCCCTACTTTGTTTTAGCTCAATCCCCTACAAGTGTTTTTTGCTAACTTAGATCACCGGTCATTACCCAAAGGCTCATATAAGCGATTCTTAATTTGGTTTCTATGGATTTTTTAATACACGTTTGCATGATGAGGACCACAAACCCAAGTTAATTAGCCAACATTCCTTTTTTTGCATAGGGTTTACAAGATTCTAATACAGCGTTTCCTTTAAAAAGAACCCTTCATGAACTGTTATGACTTATGCCACATGCATTAATGCTTATTATTGTAGCCGTTGGTGTGGTACAATTTCTCACTAACATAGAAATGGTTGTGATTTAATGAAGGATGAGTGGGTCATATCACGTGTGTTTCGGAAGAGTAACACCACACCGATCACCAATGGAGGCTCCACCATGTCCGCTTCTACTAACTCCAAGAAGACAAGAATTAACAACACCACCTCTCTTATCCATGAACCAGGTTCACCCTCTTCAGTTTTCCTTCCACCTCTTCTAGACTCTTCTCCCTACACCAACACCACCACCAACACCTTCACCGACCATCACAATAGTTCTTATGATAGTGCCACCAAAAAGGAGCACGTGTCCTGTTTCTCCACAATAGCTGCAGCAACAGCTGTTGTCTCCCCCAACAACAACTTCAACAATGCAAGCTTCGACCTTCCACCTTCTCAGCCTCTTGCAACCGACCCTTTTGCAAGGTTTCAGAGAAACGTTGGTCTTTCTGCTTTTCCAAGTTTGAGGTCTCTACAAGACAACCTTCAGCTACCTTTCTTCTTCTCCACGGCGGCGGCGCCTCCCTTCTCCGGCGGAGGCTCCGGCGACTTCCTCAGCTGGCCAGTGCCGGAGGATGGTGTTTCCAACATGCCACTGGGCGTGTCGGAGCTTGATTGCATGTGGGGCTACTAATCCATCTTTTGTTGACTTTTTCAAGAGTTGACTACTAGGATTAGACTTTGATTATGATGTTACATTTAGAGTTATGTTTTCTTTTATGGCCTTGTTAGGTTTGTTCTTTACCTAATTGTGTGTTGCTTTTTATTGTGTTATGTTATTTTCCATAATGACCTTGTGAAGCTTGAACAATTTACTCAGGCTGGTTTTTGTTGTTTACGATTACTACCCTACTAGGGCTAGTTTGATGTAAGGGTAGTTTTGGCATTTTGTGATGTGATGTGTGTTTTGAGCTATGGTCCACTTAGGATATGTCTTTGGAGGTGGTTGCAATGTCTGACGTGTTGATCTTGAAATTAAATGCTCAATATGTAAGAGTATCAGATCCTGTGGGGCTGTTGTTTTTGAGCTATACTAGTATTTCATCTATGGTCCATATTATGTC

>*Pv*CUC1 |Phvul.011G160400|*Phaseolus vulgaris*

CAATTTCCTCCTTTGAGAAAGATTGTGCCCTTTTCTTTTCTCTCTTTCTCCTTGTCTCTTTGCCCTTATCTTCTTCTCCTGGTTTATAACTTCCTCGTTTTCTTCTTCTTCTTCTTCTTCTTCTTCTTCTTCTTCTTCTTCTTCTTCTTCTTCTTCTTCTTCTTCTTCTTCTCTTTCTTCTTCTGCATGCTCCTATGGATAGTGGCTACTACAATCAGCGCCACCACCCTCACCTCGACAACAACAATGAACAACATTTACCTCCTGGCTTCCGGTTCCACCCCACCGATGAGGAACTCATCACATACTACCTCCTCAAGAAGGTTCTAGACAGCTCCTTCACTGGCCGAGCCATAGTTGAAGTCGACCTCAACAAGTGCGAGCCATGGGAGCTTCCTGGTAACTAGTAAACAGAACTCTCATAATGTCTTGTTGTTTACATCTAACTTACGTATGTAGTAATAATAAGATGAACATGATATATACAGAACTAAGAAATAGTACTCGTTTTGAAAGTTGGAAAGTTAGTTTGCGGATATGTGTTTTGAAAACCTAAGATCATGTTGTTTTCTCACTTTGTTGTTGTTGTTGTTGGTGTTTTCGTGGCAGAGAAAGCAAAGATGGGGGAGAAGGAATGGTACTTCTATAGCCTTCGTGACCGTAAGTACCCAACAGGCTTACGCACCAACAGGGCCACAGAAGCTGGTTATTGGAAAGCCACTGGAAAAGACCGAGAGATCTATAGCTCCAAAACCTGTTCCCTCGTTGGAATGAAGAAAACCCTTGTTTTCTATAGAGGAAGAGCTCCAAAGGGTGAAAAGAGTAACTGGGTCATGCATGAGTATCGTTTAGAAGGCAAATTCGCTTACCACTACCTCTCTAGAAGCTCCAAGGTAACCAAATTCATCATCATTGTCATTCTCTAACACCACATTTCTTCTGAATCACTTTAATTGGACTTCATTTATTGCTTTCGTGGTTTTTCTCTTCCTTTTGATTTTGGAATTTCGGTTCACGATTGGACTTCATAGTACATAGTTTCTTAGTTTCACAAATACTCTAAAGCTGTGGTTCTACCACTTAGCCAACGGTTATTACCCTCTAGTGCAAAGGCACGTTCCTTAGCATGAACAAGCTTGCACTACATGGGGAATATAAACTATTATCAAAATGATGACAACAAGGTATAATCATCAAAAGGGCACAGCAACTTAGCCACTGTATCATTATCATTAGTGAACTTTTTACCCTCACCCTCAGTGCTGCTACCAAGCATAAGATCCTGAATCCTGCTTCTTGATCTCCTTTTTAAGACATTCTGCTGCACTCTATAAACAGTTACTTGTTTATTATCACACATTGTAAACCTTCTTATCCAATAAAACTTATGACAAATTGCGAGTATTATTACTTGTGTTTTTAGATAAACATAGTTCATTATTTTGATGAAATTTATTAAAAATGTTAAATTTACGACTATATTAAAAAGGTTATATAGGGTTTCTTAAGAAAAACGGTGTATATATAGACATTGTTTTATTATTCTTATACGCAACTGCACTTTCATTGATTGATTAATTAAAGGGCTAATTTTGTTGTTCATTTTTTGAAATTTGGAAGGATGAGTGGGTGATATCGCGTGTGTTTCAGAAGAACAACACCGGCGGTGCCTCCACTGTGTCAGCCGCCGTAGCCACCGGCGGTTCCAAGAAAACGAGAATAAGCACATCAAACACAAGCAGCAACATGAGTCTCTGCCCCGAACCGGGTTCACCCTCTTCCATTTACCTCCCACCGCTTCTAGAATCTTCTCCATACGCCGCCAGCAGCAGCGCCGTCGCAACATTCAACGACCGCGAAAACTACTCCTTCGAAAGCGCCGCCGCCGCAGCCGCCGCCAACCAAAGGGAGCACGTGTCCTGTTTCTCCACTTTATCCACCGACGCTTCCGCCTTCAACCAGCTCGCTCCGCAGCCGGAGCCGCCACTCGACCCTTTCTCCCGCTTTCACAGGAACAACGTTGGACTGTCCGCCTTCCCATGTCTGAGGTCCTTGCACGACAACCTTAACCTCCCCTTCTTTTTCCCTCCCATGGTCCATGGCGGCACTGATGTAGCAAATTTTAGCGCCGTCGCAAACTTTCCGGCGCCGGAGGATCCGAGGGTGGTTGACGGCAGCTCTGGCATGTCGATTGTACCGTCCGAATTGGATTGCATGTGGGGCTATTGATTGTTTTTTGCGTTGACCATGATGAATCTTACTTTATTAGGCTTTGAATATTTCCTTAGGCTAGTAATCTATGGTTTTATTATTTGGATCGAACTTTGTAAGGGTTGTTGATTGTGATTGTTAGAGTGGTTTTGGTATTGCGGTTGGGTTGCATTTAGGTTAAGAACTTACCAGGCTTCAATGATTTAATGTGACTGCGAG

>*At*CUC2 |AT5G53950|*Arabidopsis thaliana*

AAACAAAAGCTTTAGATCTTTCTTCTTAATGGACATTCCGTATTACCACTACGACCATGGCGGAGACAGCCAATATCTTCCACCGGGTTTCAGGTTTCATCCCACGGACGAAGAGCTCATCACTCATTACCTTCTCCGCAAAGTCCTCGACGGTTGCTTCTCAAGCCGTGCCATCGCAGAAGTTGATCTCAACAAGTGTGAGCCTTGGCAACTTCCCGGTAAGAAAATACAAAACTAACTTACTTGTAGTTCTTTTGTAATTAAGGTTTCTGAAATCTTTACTCTCAATGTTACTTAGGGAGAGCTAAGATGGGAGAGAAAGAATGGTACTTCTTTAGCCTCCGTGACCGGAAGTATCCGACGGGACTGAGAACTAACAGAGCAACTGAGGCTGGTTACTGGAAAGCTACCGGAAAAGACAGAGAGATCTTTAGTTCAAAGACTTGTGCACTTGTTGGGATGAAGAAGACTCTTGTCTTTTACAAAGGAAGAGCTCCGAAAGGAGAGAAGAGTAATTGGGTTATGCATGAATATCGTCTTGAAGGCAAATTCTCTTACCATTTCATCTCAAGAAGCTCCAAGGTACTTCGATTTAACCTTTCATTAGTTATTTTGTTGATAAAGTAAAGCCTTAATTTTCTTTGATTAATAAATTTACAATCTATTATTTGGAAAAAAAATTCGAGAGTGTGACATAGTCGGTGACGTTGATTTTTTTCTCTTTTGCTTTATTGCATTTAATTTTGATGGCTGAAAAGGCATGGTTCTTTACTGTGGTTATCTGAGAGCATTATGGAACATGAAATAGAAAAGATCTGCTGTTTTTATGTATTGGGATTTCAATCATAATGGTGTTTTCCTAAAGTAAGAAAACATTCAATAGCTCTGATAAAATAAAATATTAATACATCCTACTTTTGGAAAGCTTAATGCAAAAAACCAGAACCAATGCAAATCTTCAGGATTTTTTTCTGACCTAGATATATTAAAAAGAAATTTAATTAAATTAATTTTACTTAATTAGTGTTCCATATCCATTGTAGACTTGTAGTGTCCAATGTACATTCCCTCTGTGCACGCTTTTACTCTATGGTTTTTCTTAATCTTCACTCTCTTTTATTTCCAAGATCTAACATTGACTTTCTCATCAAACATATATTATATAAAATATTTGACTTTTCAAAACTCTAATTTATTCCTTCAGGATGAATGGGTGATCTCTAGGGTTTTCCAGAAAACCACTTTAGCTAGCACCGGAGCCGTCTCCGAAGGAGGAGGAGGAGGAGGAGCAACTGTGAGCGTAAGCAGCGGTACTGGTCCATCTAAAAAGACGAAAGTACCCTCAACAATCTCAAGAAACTATCAAGAACAACCAAGCTCTCCTTCCTCCGTCTCACTCCCACCTCTCCTGGATCCGACCACTACCCTCGGCTACACCGACAGCAGTTGCTCCTACGACAGCCGTAGCACCAACACAACCGTCACAGCCAGCGCAATAACCGAGCACGTGTCCTGTTTCTCCACTGTCCCTACTACTACTACGGCCTTGGGCTTAGACGTTAACTCATTCAGCCGTCTTCCACCGCCGCTAGGGTTTGACTTTGACCCTTTTCCTCGTTTCGTTTCTAGAAACGTCTCGACTCAATCTAACTTCAGATCGTTCCAAGAAAACTTCAATCAATTTCCTTACTTTGGATCGTCTTCTGCATCGACTATGACCTCCGCCGTTAATCTGCCTTCTTTCCAAGGCGGCGGAGGCGTCTCCGGGATGAATTACTGGCTACCGGCGACTGCCGAAGAGAATGAGTCAAAGGTCGGTGTGCTTCATGCTGGACTTGACTGTATTTGGAACTACTGA

>*Br*CUC2 |Brara.J00883|*Brassica rapa*

ATGGACATTCCACTTTACCACTATGACCACGGCGGAGACAGCCAATATCTTCCGCCAGGTTTCAGGTTTCATCCCACAGATGAAGAACTCATCACCCATTACCTCCTCCGCAAGGTTCTTGACGGTTGCTTCTCAAGCCGCGCCATTGCAGACGTTGATCTCAACAAGTGCGAGCCTTGGCAACTTCCCGGTAAGTGAAAACAAACCAACTTAACTTACTAACATATATTTCTTGTCATTTTTTTGAAGCAATTTAGTTTTCACTAATTGAACTACTCTCCAATGAACTTTTTAGGGAAAGCTAAGATGGGAGAGAAAGAATGGTACTTTTTCAGCCTCCGTGACCGGAAGTATCCGACGGGATTGAGAACGAACAGAGCAACGGAGGCTGGTTACTGGAAAGCTACCGGAAAAGACCGAGAGATCTATAGTTCAAAGACTTGTGCACTTGTTGGGATGAAGAAGACTCTTGTCTTTTATAAAGGAAGAGCTCCTAAAGGAGAGAAAACTAATTGGGTTATGCATGAATATCGTCTTGAAGGCAAATTCTCTTATCATTTCATCTCTAGAAGCTCAAAGGTATTTTAATTAAATTTGTAACCTTTTTATTATTTTCTTACTAGAAACCTTTTTTTATCTCTGATTAATAAATTAGTATATAATCTTTTGCTTCTTTATTTTGTTTTCTAAGAGTATGACATAATCGGTGACGTTGAGTTCTTGTTTTGCTTTTTTCTGCATTTAATATCGATAGCTGAAAAAGCATGGCTATTTATTAGGGTTGAAGTGAGATCATCATGGATCATAAAATAGAAAAGATCTGCTGGTTTATTTCTTGGGATTTCAATCATAATGGTGTTTTCTTAAAGCATGATTTTTTTTAACTTTTTGACAAAATAAAAAATAAATACATCCTACTTTTGGAAAGCTCAATGCGAAACCCAAATCGTAAACCCATATTCAGGATTTTCTGACATGTCTTCAGGATTTTCTGATATATATTAAATATTAAAATTTCAGTAAGTCTATTTTAGGACCCTTCTCTTTTAATTAATTTTACTTAACGTTATTCTCCCAAATTCTTTTTACTCCCTCAGGACGAATGGGTGATCTCTAGGGTTTTCAAGAAAACCGGTTTAGCCAATACCGGGGCCTCCGGGGGAGAAGCAAGTGCTAGCGTAAGCAGCTGTACCGGTGGGTCTAAAAAGACGAAAGTACCCTCAACCATCTCCACAAACTACCGTGAGCAACCAAGCTCTCCTTCCTCCGTCTCACTCCCTCCTCTCTTTGACCCCACCACAACACTCGGCTATACCGACAGCTGCTACTCCTACAACAGCCGTAGCAGCAATACAACCCTCACAGCCACTGCGATAACCGAGCACGTGTCCTGTTTCTCCACTGCCACGACTACTACTGCCTCAGGCTTAGATGTTAACGTTGACTCATTCAACCATCTTCTACCGCCTGCTCCGCCTGGGTTTGACCATTTTTCTCGTTTTGGCTCTAGAAACGTTTCAACTCTATCTAACATAAGGTCGTTCCAAGAGAACTTCAATCATTTCCCTTACTTTGGTTCGTCTTCTGCATCGACCATGACCCCCTCCGTTAATTTGCCTTCTTCCCACGGTGGCACCGGAATGAACTACTGGCTACAGACAACCGCGGAAGAGAACGAGACAAAGGCTGGTCTACTTAATGGTGGACTAGATTGCGTATGGAATTACTAA

>*Dc*CUC2 |DCAR_019571|*Daucus carota*

ATGGACCATTTCTACCAAAGTATGGAGAACAATGGGGATGCTCAGCTGCCTCCAGGCTTCAGATTTCACCCAACAGATGAGGAACTCATCACTTACTACCTCCTCAAGAAAGTCCTTGATCACAACTTCAGTAGCAGAGCCATTGCCCAAGTTGACCTCAACAAATGTGAACCATGGCACCTTCCTGGTAACTTACTAACTAACATAGTGAAGTTCACTTATTCAGTTTTTTTAATTGGTTAGACACGCACATCTGAGAGTCGAACTCCTGACCTCCCGTAAGGGGTACGAGAGCTCAACCACCGCACCAACACGTTGTCAGCATCTTTTGTTCAGTTTTTCTCTTTATTGTTGAATTATTTACAGTTGAGTTTGTTTTTTGTGAATGTAGAGAAAGCAAAGATGGGGGAGAAAGAGTGGTACTTTTACAGCTTGAGGGACAGGAAGTACCCAACAGGGTTGAGGACAAACAGGGCGACAGAAGCGGGGTACTGGAAGGCCACAGGGAAAGACAGGGAGATTTACAGCTCGAAGACTTCGTCTCTGGTGGGGATGAAGAAAACCCTAGTGTTCTACAGAGGCCGTGCTCCTAAAGGAGAGAAGACCAACTGGGTCATGCATGAGTTTCGCCTTGATGGCAAGCTTGCTTACCACTACCTCTCTACTACCTCTAAGGTACACATCCATATATTATTACTCAAGCTATCTCTGTATAAAATGCCTCATCATATGTCACTAGCGCACACAGAGAGAAATGGGGGAGAGGGAGAGGGAGAGATGGGGGGAGAGGGAGAGGGAGATAGTGAGAGAGAGAGAGAGAGAGATGGGGGAGAGGGAGAAGGAGAGAGAGAGAGAGAGATGGGGGGAGGGAGAAGGAGAGAGAGAGAGAGATGGGGGAGAGGGAGAGAGATCTTCAGAGTTACTGCACAAGCAAGGGCTAGGGACAGTATTTGTTACAGTTCTTAGCTAGGAACTAGGGTAATGTTGTTTCAGGTTTTATGTTACTATTAACATATCTAAAATGTGCATGTAAGCTAGAATCTGACCTAAATCAAATCTAAAGAGAAAATATCATGTTTTTTTTTAGCACTCTACACACCTACACTACTTGTAAATTCATCTTTACATAGTCATGTCAACTCAACATATCTACACATCCACAAACCTACTTTTTCCTAGAGCCACTCACAAATCTTTTTAACAATCCTCGCCACTCAACTAACATTAATCACATTTTTCAGGACGAGTGGGTCATCTCCCGGCTCTTCAAAAAAACCGGCGGCGCCACCGCCGGAGAAAAAAGACCAAGCTCCAGCATGAGCAGCCACTTTCACTCAGAAATCAGCTCATCTTCCTCCATCCCTTTCACACCACCTCCACCACCAGCAACAACAACTGATCACGTGATCACCTACGAGCACGTGCCCTGTTTCTCCAGTTCCGCCGCCCCCGGCGGCTTCAGCACTTACCACACCCTCTTCGACGGCGGCCTCCCTCCGCCGCTAATGGACCCCACCCCAATGCCGCCCTCCTCCACCTTCCCCAGCCTCAGATCACTAGAAGAGAATCTCCACCAGCCCAGCTTCTTCTTCCCGCCGGTCAACTACGACAACTTCCCGGCGATGGAGACACCGAAGCCGGGACTCACTGAACTCGACTGCATTTGGAGACCCTCGTTTAATTAA

>*Eg*CUC2 |Eucgr.B00529|*Eucalyptus grandis*

CTCGCCCTCCCTCAACTTTCCACCATAGCTTTCGTTACTGGCTTCCCCCCAAATATCTCTCTCTGTATTCACTGAATCTCACATCCCCTGTTTTGTCTCTCTCTGCTTGTCTGATGTATAAGCAGCATCTTGTGAGTGGTTTTAGCTCCTACCCTTCAGAAGCTTTCGCGTCCTCGGCGATTTTTTGAGGTGCTCGGTTTCGGGCTCTTCGATTCTTGAAGAATTTTCGATCTCTCTTTTCTTTGTTCTTTTTTTATCTGTGGGCGATCAGCAGTTTCGAGAAATGGAGAACATGGCTAGGCTCGGGAAGGAAGACGATCAGATAGAGTTGCCGCCGGGGTTCAGGTTCCACCCGACGGACGAAGAGCTCATCACCCATTATCTGCAGAAGAAGGTGGGGGACACTGGCTTCTCCGCCAAAGCCATCGGAGAAGTGGATTTGAACAAGTCCGAGCCCTGGGATTTGCCTTGTAAGCAATCACGAAATCGCTCCTTTCCGCTTATTTCTGTTCGCCTCCGATTCATTCTGAAATTAGATTAACATGCGTGATTGCTTCCGTTTTGATTATAGGGAAGGCGAAAATGGGGGAGAAGGAATGGTATTTCTTCTGCCTGAGGGACAGAAAATACCCGACTGGTTTGAGGACCAACAGAGCCACCGAATCTGGTTACTGGAAGGCCACGGGGAAAGACAAGGAGATCTACAGGGGAAAATCTCTGGTTGGTATGAAGAAAACCTTGGTTTTCTACAGAGGGAGGGCTCCAAAGGGGGAGAAGACGAATTGGGTCATGCATGAATACAGATTGGAAGGAAAACTCTCTCTGAATTATCTCCCCAGGGCTTCGAAGGTGATAATGAATGGCCGGTGCTCTGTTTTTTTCCCATGTTTTTCTTTTGAACTTTTCGCGTGATGATTTAAAGCGCACGAATTTATAGTTTCTGTTCTTTCGCGTTGCAGAACGAGTGGGTCATTTGCAGGGTCTTCCAGAAGAGCTCTGGTGGGAAGAAAATCCACATCTCGAGCCTCGTGGCGGCGGGGTCTCTCGAGAACGAAATGAGCTCCGGCTTGCCGCCGTTAACGGATTCCTCTCCTCACGATTCGAAGACGGAATCCAACCCCGGATCGGCTTACGTGCCCTGCTTCTCCAGCCCAACAGAGTTCGAAAGGAACAAGGAAAACACGAACAATTACTTCAACAATCCCATGTTCCCCATCTCCTCGAACCCCACGAACACCACCCCCAAAATCTCGCTCTTGAGCCCAGTGTACCCTCACCAGGCCATCCCCGTCCCAGCCAATTGGCAACACCCGGGGGGCTCCGTCTTCATGCCCGAGCACTCGGTCCTCAGGGCTCTGCTCGAGGGCACCGGGCTGAACGCGAGGCAGAGCGCGAGGGCGGAGCGGGAGGCGATCAGCATCTCCCAAGAGACAGCGCTGACCAACGACTTGAACACCGAGATCTCCTCCGTCATGCAGGATTTCGAAATGGGGAGGAGGCAGTTCGAGGATCAGCAGCAAGTTCCATCGACCTTAGCTGGACCAATGGACGTGGACCTCCTCTGGAACTATTCAAGTTAGAAAAAAAAAAAATATCCGCGCACGTTCCTGAGCAAGATACCTATTGAGATTGGTCATTTGTTATGTTAAAATTTATGGGTGAGCGTGGACTTGTTGTATAGATTTTCTGAAATCCCAAGTAGTGATCGAGAGATAGTGAAGCATGGTTTCTGTAATGATTGTCTATATGCATTTGTAAGGAGATTCTAGTTGCAAGTTCCTCAATGAATATTTTAGAAGCGTGATGTTTCTGTAGC

>*Gm*CUC2 |Glyma.13G274300|*Glycine max*

TGCTAGTTGCAATGCTACTATTATCTCTCATGAACACACTTTCTGTTCCCTTTCTTACAATTTCTTCACTGACAAAGGAATACCTGCACATTTCCTCACGCACGCACGCATACTCAATCGCCACTCCCTCGTACATCACCCTCTCTCTCTTTCTCTTTCCTGCTTTCTCCCTCTAGCTATGTGGATCATGATGAGTAACCTGTCAGAGAGTAGAGAGAGCTATCAGAAGAGGATAGTGCAGAGAAAACACAGATTTTTCCAGCCATATCATACCTTATTATTTCACCTTTTTCTTTCTGCCACCCGATTCATCAAATCCCCATGAGAACACCCCTTATAAACCACACACCCTCATCCCCAATCCCTAACAAAATCTTTAACCCACCAAGGTGAAGAAAAAAAAATTGAAAGTGACAATCCCTAACCAAATCTTTAACCACTTAAAACCTTCTTTCACTATTTTGCTTCTATGAACCCCTTCCCCATGCCATGCCTCTCATTTCCTCCTATATAAAAGCCATGAAGTAGTGTTATTAGTATCCAATATTCTCCTTCTACTTAAATTAAATTCTCCCTCACTTTTCATCTCTCTCTTTTGTAAGTGTTCCTTATTTTTTTCTCTCCAATGGACAACTCTTCCTACCACCACTTGGACCACACTGAAGCTCACTTGCCACCTGGTTTTCGGTTCCACCCTACAGATGAGGAGCTCATAACTTACTACCTTCTCAAGAAGGTTCTTGACAGCACCTTCACTGGTAGAGCCATAGCTGAAGTTGACCTCAACAAGAGTGAGCCATGGGAGCTTCCTGGTAAAATTAAGCACTTTCAACGCCCTTTCTTTTTTTCTTTCTTATCTTTGGCTATTAATATTTCTTTCATTACTTGCTATAGTGGTAGTACAAAGAAGTTTGGATTTTCCACCTAACGTAACCTTTGTGGTTGTTTGTTTTTGGCAGAGAAAGCTAAGATGGGAGAGAAAGAGTGGTACTTCTTCAGCTTACGTGACAGGAAGTACCCAACTGGGTTAAGAACCAATAGGGCTACTGAAGCTGGTTACTGGAAAGCGACTGGGAAAGATAGAGAGATTTATAGCTCAAAAACTTGTTCTTTGGTGGGGATGAAGAAAACCTTAGTTTTCTACCGTGGTAGAGCTCCAAAGGGAGAGAAAAGCAACTGGGTCATGCATGAGTATCGCCTTGAAGGCAAATTTGCTTACCACTACCTTTCTCGCAACTCCAAAGTTAGTACTATCATTTTCATTTGGTTCCTTCATTAATTCATCTTGCTATCTTCTTTCTATGTTTCTTTACTTTTTTCTCTACTCTCACATAGCATTGAACCATGTGAACTTTTTAACCCATTAAGAGCCGTTTTTTTTACACCCTCCAAATTTGCAGTGAAATAAAGTTAGGTGGAATCTGCTTTCCCCTTTAGCCTTCTATAAGTAGGTTGCGTTTGCCAATGTCGAGATGATTTTCCCCTTTCAATAACCTGTTTCTCGATTAAGATTTTGTTCCCCTTCCATAAGTAATCTTCCCCTTGTTCGTTTTAGCTCAATCTCCTCTCAACAAGTGTCTTTTTGCTAACTTAGACCACCGGTCAATACCCAAAGGCTCATAAACGTTTCTTAATTTTTTGTGGTTTTTTTAATACATGTTTGCATGATGAGGGACCACAACCAAAAGTTAATCACAAAGTGATTATATAGGTTTACAAGATTCTAACACATCTTTCTTGTTAGGGAAAAAAATTGATTGGTACAAGCAGTTATGACTTATAATGCTTATTATTATAGTCGTTGATATGGTACACTTTCACTAACATAAAAAATGGTTGTGATTAAATGAAGGATGAGTGGGTCATATCACGTGTGTTTCAGAAGAGTAACACCGCCACCAACAATGGAGGCTCCGTCATGTCTGCTTCTAGTAACTCCAAGAAGACAAGAATGAACAGCACCACCTCTCTTATCCATGAACCAAGTTCACCCTCCTCAGTTTTCCTTCCACCTCTTCTAGACACTTCACCCTACACCAACACAGCTAACTTCACCGACCGTCACAATGGTTCCTATGACAGCATCACCAAAAAGGAGCACGTGTCCTGTTTCTCCACAATAGCTGCAGCAACAACTGCTGTTGTCTCCCCCAACAACTTCAACAATGCAGGCTTTGACCTTTCACCTTCTCAGCCTCTTGCAACCGACCCTTTTGCCAGGTTTCAGAGGAACGTTGATTTTTCTGCTTTTCCAAGTTTGAGGTCACTACAAGACAACCTTCAGTTCCCTTTCGTCTTTTCCACGGCTGCACCGCCCTTCTCCGGCGGCGGTTCCGGCGACTTTCTCAGTTGGCCGGTGCCGGAGGAGCAGAGGCTGATAGATGGTGTTTCCAACATGCCACTGGGAGTGTCGGAGCTTGATTGCATGTGGAGCTACTAATTCAGCTTTTGTTGATTTTGTCCAGAGTTGACTACTTGGATTAGACTTTTAGACTTTGATTATGTAACATTTTGTTATGTGTTATGTGTTATGTTTTCTTTATTGCCTAATGTGGCTTGTACTATAGTACTAGTACTTGGTTTGTGTGCTGCTGTTGATTATGTGTGATGTTTTCCTTAATGGCCTTGTATGAGGGTTGAACAATCTACTTTGGTTGTTTTTTGTTTTTTATTACTAGCCGTCTAGGGTTAGTTTGATGTAAGGTTAGTTTTGGTATTGTGTTTGAGCTATGGTACACTTAGAATTTATGTCTTTGGAAGTGTTTGCAATGTCTGACGTGTGGATCTTCAAAGTAAATGCTCAATATTTAAGAGTATCAGATCCTGTGGGGTTGTCGTGTTTGAGCTATGTATGTATGTATTTCATTTATGG

>*Gr*CUC2 |Gorai.013G171300|*Gossypium raimondi*

GAAACAGCTTTATCGATTCATGGTGAAAAATCTTTTCTTTCTTTTCCCTTTTCCTCCCTCCTCTTTAAAATCCCCCCTCCTAAAGACTTTACTCTCATAAAAGTTTCTTTCTGTTTCCTACCCTTTCATTTTCTCCCTTCACTGACAGTGAGAAACCTGCACTGCACTGCACTGCACTGCACCCTTTTCTCACGCACGCACGCATACTCAAAAACTCAACCCTTTTTCCCTTCACACACAAGGGAAAGGCAATTTGAGAGATTTCAGAACACTTCTGAGCCTCTGATAAAAACAAACACACATCTCATCACCTTCAGATCATGCCCTTCCTTACTATATAATTGTTTGCCCTTTTGGCCCCTCTCTCCATTCTTCAAATCCACCCCAATATAACACCCTCTCTTCATTCCTTTCCTCTTCAGTTTCAAACCCTTTTTTTCTTCACTTCTTGCTGATCAACTTTGTCTTTTGAAGTCCAGGGTTTTCGGCTATGGATAGTTACCATCATTTTGACAATGGTGAAACGCATTTACCTCCAGGTTTTCGTTTCCATCCAACTGATGAAGAGCTCATTACATACTATCTGGTGAAGAAAGTTCTTGATAGGAGCTTTACTGGTAGAGCCATAGCTGAAGTTGACCTCAACAAGTGTGAGCCTTGGGAACTTCCTGGTCAGTAATCATTAACATTTTCTGCAACTGCCCTTCTCTTTGTTGCACATAAACCGATGTAATGATGATGTTGTTATTTAAACTCTTTATGTTTTTTGTATTGTTCATGGGATTGTTTTTGCAGACAGGGCAAAGATGGGAGAGAAAGAGTGGTACTTTTTTAGCCTAAGAGATAGGAAGTACCCAACTGGGTTGAGAACTAACCGAGCTACTGAAGCTGGTTACTGGAAAGCTACTGGGAAAGATAGGGAGATTTATAGTTCAAAGACTTGTGCACTTGTTGGAATGAAGAAAACTCTGGTTTTCTATAGAGGAAGAGCTCCTAAAGGAGAGAAAAGCAACTGGGTCATGCATGAATATCGCCTTGAAGGCAAATTTGCTTACCATTATCTCTCTAGAAGCTCCAAGGTTTCTCTCTATCCTTCCCTTTTCCTCTCACTTGACTGCTTCTTTTAGTATGATAAACTTGTTGATCAGTCTAATGTTTTGTTGAAGTTTTTCATCTAACTGATCAATCTTTGGATAGTACTCTAACCTAAATGAGGAATGATAAGATGAGGTTAGCCTTATTATAAGGCAATTGTGTGTGGATTGTAAGTTAATTGTAGTCATGGTTTTGCTTACAAAGGCAAACTATTAATCTGGCATATGAAATTATAAACTTCAAGTTTTTTCTCACTCATTCCAATCGTGAAAACCTTTGCCATTTCCCATTTTGCTGCTGAAAATCTAGGTTTTTTCAGTAACATTGCCTGTTAAAGCAATGGTTCTTTGAACAATGAACTGAAACTCATTAATCTGTGTCATAGCCAACCCTTGTTACTGTTCTTTTCTTCTTTTTTTCCCATAAAAAGCTTTCTAGCCTTGCTTTTCAGATTAACACTGAAAATGTAAGATTAACAGTAATATGGTCTCTTAAAAAGGTGTTTTTAGTCTTTGGACTTCTAGCAGTGAACTGAATCTCTCTAATCTGTAAGTTAAAAAGCAAATGTTATCTTTCTGATTTTCACGTTTTTACAATTGAATGGCTTTCCCTGAATCCATGTTAAGATTACCATTTAACACGCAAGAACTCAGTACCATACAGTTTCCCATTTTCACTCTTCTTTTAACACGAAATCCAATGAAAAACACCCCCTCAAATCTCGTTTATGAAGTATTTGGCCATTGTACCATTATCTTCCATTTTCATGGTAATTTGGAACCTCTAGATGTTGTTTCACTGTTGCGTTAACTTCTAGTTGCTTGGATGAAAAGAGAACTATTAACTATTTGATGCAACACTAAGGTTTTTTTTTTAAAGTTATTCCAGGATGAGTGGGTAATATCCAGGGTCTTTCAGAAGAGCAGCGGAGGAGCCAAGAAGGCCCCCATGAGCGCCGCTTCCATGGTGCTCTACCAAGAACCAAGCTCACCTTCCTCGGTCTCTCTTCCACCGCTCCTGGATACCACCAATGCTACTGGCAGTGGTACCGCTACCGGTGCTTCCCTCACTGACCGTGACAGCTGCTCTTACGACAGCCATAACCAATCCGAGCACGTGTCCTGTTTCTCCACCATTGCTGCCACCTCGTCAGCCACCCTTCCTGGCTACCACAGCGGATTCGACCTTGCATTGCCAACCCCACCCCAGATGAATAACAGTTTTGATTCAATTGCAAGGTACACAAGAAATGTGGGTGTTCCAGTGTTTCCAAGCTTGAGGTCTCTCGAGGAGAATTTGCAGCTCCCTTTCTATTTCTCGGAGCCAACATTGGCGGGGGCGGCACCACCACTTGACGGTGGTTCATCAGCGAACTGGGGAGCTGTTTCTGAGGAAGGAAACAGTGGTTCTGTTGCTGATGGCAAGATGTCCAATATAGGTCCTACTGAGCTTGATTGCATGTGGACTTACTAAGTAGGAAGTTGAGGAAGGAATTTCCATTTATGTGTTTGAAAAAGACTGCTGTCTAATGCTAGCTATAATGTAGTTAGATTTCAATTTCATGACTCTTTACTTTTAATGAATCCTAGTATTTGCTGCCTAATGTTTTACATCTTTTTCCTTTA

>*Cs*CUC2 |orange1.1g047710m|*Citrus sinensis*

ATGGAGATCACCTACAATTACTTTGACAACAGTGATGCACATTTGCCTCCTGGCTTTAGGTTTCACCCAACTGATGAAGAACTCATCACTTACTACCTTCTCAAGAAAGTTCTTGACTGCAACTTCACTGGCAGAGCCATTGCCGAAGTTGACCTCAACAAGTGTGAGCCCTGGGAGCTTCCTGGTAAGCTGAGTTGACAAACCCCCCCTTTTTTTACCATTGTTCATACTTGATATGCGAATCTGTTGTGACATTTTTTGTTACTTTTGTTTTCGCTTCTGCAGCTAAGGCAAAGATGGGCGAGAAAGAGTGGTACTTCTTTAGCCTGAGAGACAGGAAGTACCCAACTGGGCTGAGAACTAACAGAGCTACGGAGGCTGGTTACTGGAAGGCCACTGGGAAGGACAGAGAGATTTACAGCTCCAAGACTTGTGCTCTTGTGGGCATGAAGAAGACTTTGGTTTTCTACAGAGGCCGAGCTCCTAAAGGAGAGAAAAGCAACTGGGTTATGCATGAGTATCGCCTGGAAGGCAAATTTGCTTATCAATATCTCTCCAGAAGCTCCAAGGTATTCTATATATATCACATACACGCACATTCATATTTCATTAGACATTTTAGTAGTGTTTAGGGTTTTGCTGTTAGGTTTATGGCCATGTTTGGTTCTTTGGGAGATAATACAAACTACAAACAATGAAGTAATTCATGTTCCTCTTTCCCGTTAAGCTATCTTTGTTTTTCCTCTGAAACAACTCATGCTTATGATCACAAGATGGATCATGGTAGTTATTATCACAGTGCAATTGAGAACTGATTGATAAGGCCAGCTCTAATGCTGTTTTTGTCATTTAAGTGGGCTGAAAACTCAAAATGGCATTTGCTTAAATTTAATATCATAAGTATTATTGAGATTTATTCTACAACATTTTAATGTTATTTCAGTGGGCAGGCACCTCACGTCTCTTCATTCATGTACACATCTTTACTTATCACAACTTGTTTAAACAATGTACACACATCTTACACTTAATTTGGTGTTAAGATACAACTTAAATAACTCTTAACCCTGTTTTATGTCAGGCTAAAAAGTTAAGCTTCTTGCATGGCATGAACATGCTTGCCCTAGCTTTACCTTTTCCTCAGCTTTTTTGAATCAAAATTTTTTTCTAACCGTCAATACTCGTAATGATCCCCTTTGGCTTATTCCTATGTTATTTAGCATAACGATAGCATTATCATTCCGTAAAGATTATCCAGTGCTTTTTCTTTTCTCCTGCTTTCCACTTTGCTTGTCTTGATAAGCAAAAGCTAGCCTCCTTTGCCTTTTATCATACATTATGACATTTTGCGTTGCAAATAGCACTTTTTTTTTTTTCAGTTGCTTGATGCATTTTATGTACATCACTTCACTTTTGAATTTTGATTATTAACCTTTTCTTCTTTGCCAGGATGAATGGGTGATTTCAAGAGTATTTCAAAAGAGCAGTGGAGCCATCGCCACCGCGGCTGCCGTCGCAAACGCCGTCAAGAAAAGTCGTTTGAGCTGCACCATTTCGTCATCTTCAACCTTCAATCACTCGTATCCGGAACCCAGCTCCCCTTCATCAGTTTCTCTTCCTCCTCTCCTTGATCACCCCACCATTGCTGCCGCTGCTAACGCCACCACTGCCCCCAATGACAGCTGCTCGTATGATGAAAGCCACGCTCCTTCTGATCAGCACGTGTCCTGTTTCTCCACCATTGCAGCCGCCGCAGCCGCAGCCGCAGCTTCGGCAGCCACTGCCACCACATTCAACACCAGCTCCTCAGCTTTTGACTTCACTACAGTACCAGCGCCTGTTATCAATGCTGATGCTGGTGCCGGCGCTGCTTGTGACCCGTTTGCTCGTTTTGGAAGAAACAATGTTGGCTTGAATGCTTTCCCTAACTTGAGGTCTCTGCAGGAGAATCTTCAGCTTCCTTTCTTCTTCGCACCACCTGCTTCTTCAGTTGCGCCTCCTCCCTTTCAGGGTGGCGGTGGTGGGTCAAACTGGTCAACGGTGATGCAGGACATCGGCGGTGGCGGTGGTGTTGTTGGTGGTGGCGGCAGGTTGAATGTGGGTCCCACTGAGCTTGATTGCATGTGGACTTACTGA

>*Pv*CUC2 |Phvul.005G074500|*Phaseolus vulgaris*

ACAATTTCTTCACTGACAAAGGAATACCTGCACATTTCCTCACGCACGCACGCATACTCAATCGCCACCACTCCCTCGTACATCACCCTCTCTCTCCCTTCCTCTTTCTATTTCTCTCTCTAGCTATGTGGATCATGATGAGGACCCTGTCAGAGAGTAGAGAGAGCTATCAGAAGAGGATAGTGCAGAGAAAACACAGATTTTTCCAGCCATATCATACCTTATTATTTCACCTTTTTCTCTCTGCCACCCGATTCATCAAATCCCCATGAGACCACCCCTTATAATCCACCCTCTCATCTCCAATCCCAAACCAAAAAACCTCAACCTCCAAAGTGAAAAATAAAGTAACAACCCCAAAGCCCAAGCCAAGTTATAAATCCCTCTTTCACTATTTTGCTTCTGTTGTTCCCCTTCCCCATGCCATCACTCTCATTTCCTATATATAATCAAAGCCATGAAGTGTTAGCATCCAAGATTCTCTTCTTCTGATTCCATTAATTTCTCTCTCACTTTTCACCTCTTTTGCAAGTGTTCCTTCTTCCTTTTCTCTAATGGACTCCTCCTACCACCACTTGGACCACACTGAAGCTCACCTGCCACCTGGCTTTAGGTTCCACCCCACTGACGAGGAGCTCATAACGTACTACCTTCTCAAAAAGGTTTTAGACAGCACCTTCACTGGTAGAGCCATAGCTGAAGTAGACCTGAACAAGAGTGAACCATGGGAGCTCCCAGGTAGACGCTTAACAACTGTCTTCTTTTTTCTTTCTCTTTCATTGAGTTATTACTTGTTACTCACTTAGCACTTTCCATACAAAGAGGTTGATGATTTTATCTAACTTTGTGGTTGCTTGTTGCTGGCAGAGAAAGCTAAAATGGGTGAGAAAGAGTGGTACTTCTTCAGCTTACGTGACAGGAAGTACCCAACTGGGTTACGAACCAATAGGGCTACTGAAGCTGGTTACTGGAAAGCCACTGGGAAAGATAGAGAGATTTACAGCTCCAAGACCTCTTCTTTGGTCGGGATGAAGAAAACCTTGGTTTTCTACCGTGGTCGAGCTCCCAAGGGGGAGAAAAGTAACTGGGTCATGCATGAGTATCGCCTTGAAGGCAAATTTGCTTACCACTACCTTTCTCGCAACTCCAAGGTCATTATTACTTTATTTAATTAATTCTTCTATCTACTATCTTCTATTTTGTGTTATTACACTCAAAGCATTGAAGAGTGTGTACTTTTTACCCATGATGAGCCGCATTTTCACTCTCCAAATTTGCAGTGAAATGACTTTAGTTAGGTGGAATCTGGGTTTCCTTTTAGCCTTCCACAAGTAGGTTCCATTTACCAATGTTGTGATGAGTTTTCCCTTTCAATTGCTTCCTTGTTTTGGTTCTCGATTAAGATTTTGTTCCTCTTACCTTTCTTATTCTCGCTCAATCTCCTCTCAACAAATGTTTTTTTCACTTTTGACCATCAGTTAGTACCCTAAGGTTCATTTAAATGTTTGTCAAATTGCCTTCTGTCAACACATGGTTGCATGAGCACCACAATCCAAAGCTAATTAATTAAAACGTGGTTATATCCGGGATCCGTATTCCATTTCTGCAAATGGTTTACAAGATTTCACAGGAACAGCTTTTTTTCCACACGGCTATTTTCCCAACGACAATTTAAGATTTTGTCATGATTTATTTGCAGTTATGCAACGTGCATTAATGCATATTGTTATTGTTGGTGTTGTACTAACACGAACCATTGTGATTTAATGAAGGATGAGTGGGTCATATCGCGCGTGTTCCAAAAGAGCAACACATCCAACGGCGGCTCCGCCATGTCTGCTTCAAGTGGCTCCAAGAAAACAAGAATGAATACCACCAACAGCTCTCTCTGCCCAGAACCAAGTTCACCCTCTTCAGTTTACCTTCCGCCTCTTCTAGACTCTTCACCGTACGCCAACACAACCACCGCGGTCAACTTCACTGGCCGTAACAACTGTTCCTATGACAGCACCACCAAAAAGGAGCACGTGTCCTGTTTCTCCACAATCGCTGCAGCCACCGCTGCTGTTGTCTCCCCAAACAACTTCACCAATGCAAGCTTCGACCTTCCACCATCTCAGTCTCTTGGAACGGATCCCTTCGCTAGGTTTCAGAGAAACGTTGGCGTATCTGCCTTCCCAAGTTTGAGGTCACTGCAAGACAACCTCCAGTTACCGTTCTTTTTTCCTCCAGCAGCACAGCCCTTCTCCGTCAGTGGCACCGGTGATCTCCTCTGGCCGATGCCGGAGGAGCAAAGGCTGGTTGATGCGGCATCCAACGTTCCACTGGGGGTGTCGGAGCTTGATTGTATGTGGGGATACTAGCTGATCTTTTGTTGACTTTCCAAAGCGTTGACAAGCAAGAAGAGACTTTGATAATCACACTTCGTTATGAGTTATGTTTTTTAACCATTTAAGTTATGTTTCATTATGAGCTTGTATGGCTTGAACTAGGACTATGACATTCTGTGTGTGTGTTTTGTTTTGTGTTTACTAGGGCTATTTGATGTAAGGGTAGTTTGGGTATTATGTGGTAGTGTTGTGTTGTGGTATGGTCCACTTTTGGATTTATGTTCTTGGAAGTGTTTGCAATATCTGACGTGTTGATCGTGAGAGTAAATGCATGTTATTAAAGGGTTTCAGATCGTGTGGGGTTTGATTTTGATCTTTGG

>*Sl*CUC2 |Solyc07g062840|*Solanum lycopersicum*

ATGGAGATTTATCATCAGATGCAGTTTGATTGCGGTGATCCGCATTTACCACCGGGGTTTCGGTTTCATCCAACTGACGAAGAACTTATTACTTACTACTTGTTGAAGAAGGTTCTGGACTGCAACTTCACTGCTAGAGCTATTGCTGAAGTTGATCTCAACAAATGTGAACCTTGGGAACTTCCTGGTATGAACTTTTTTTTTTTATTCTCATGTTATATTTGAGTGTGCAAATTTACCATGTTTTACAAGATTTGAACGCTAATTAAATATTTTAACATACTTTTGAGCTTTAACTAAAAAAAAACTAAAATACCCTTATTTTATTAAGGGAAAGCGAAAATGGGAGAAAAAGAATGGTATTTCTTCAGTCTACGTGATCGGAAGTATCCAACAGGGCTGAGGACTAACAGAGCTACTGAAGCGGGTTACTGGAAAGCTACTGGAAAAGATAGAGAAATTTTCAGTTCAAAAACATGTGCACTTGTTGGTATGAAGAAAACCCTAGTTTTTTATCGAGGAAGAGCACCAAAAGGAGAAAAAAGTAACTGGGTTATGCATGAATATCGCCTTGATGGCAAATTTGCCTATCATTATATCTCCAGGAGTTCGAAGGTACTAGAGTATTTTTGTGTTAATAATTAACTGATACACATATATTAATGAATTTTGATGAAGAAAACTGATCTACATGTTTTGTGTTACAACTCATACTTTTTTTTGTATTTGGATTAAAGAGATCAGATTTACTGGAGATATTCTGTGCACAAATATATCATTGATATTACTGCTAGAGCCAGAGATTCTGAAGCAGAAAGTTTTGTGAAACTTCGTTTTCAATGTCTCAATTGCTTATGATTAAGTTGGAAGTATACATAGTCTGAAAAATAACAGAAAAACAAATTAAAAGAGAAAAAAAAGAATTTGCTTTTATTATGATTTATGATCAAAGCAATTAAGACTTTACAGAATAATATACTGTCATATTGATAGCGATTTAATATAATCTCTTTCCAAACTTATTTTATTAGCCTTATCAATGTGTAAGCATGCTTACGTAAATTCATCAGTCTGTTAGATTTTGACTTTTAGCGTTTTGGCATTAATTTAAATGCTTCTTTTTTTAAAATTGCATCATAATGAGCTTCATATTTGTTAGATTACAAACATTTCTTTAATAACAAAATAAGATTCGTAGACGCTAAAAATTAGGTCTTGCCCCAAAAAATGTTGTATATTTGTTTTTCATGGTAAAGTTAAAAGCCTGATGCGGGTTTTATGAAATTTGTAGCTTTCACAAGTTCATAAAATATGTAAACATGCTTGTAGTTATAAATTTTAAATTTTGAATTCGTTTTTTCGTGTTTCCATTGTTCTCATATATTTTTTGGCTACAGGACGAGTGGGTTATCTCGCGGGTCTTTCAAAAAAGCACCGGTTCTAATGGTGCCGCTACTTCAACTGGTGGCGGCAAAAAAAGGCTAAGTTCAAGTATAAACATGTACCAGGAAGTGAGTTCACCGTCTTCCGTCTCTCACCTTCCGCCGCTCCTCGATTCCTCTCCGTATAGCACTACCGCCACTTCCGCCGCAGCTATCGTAATCGGCGACCGCGATCGTGATCATAGCTTCAAGAAGGAGCACGTGCCCTGTTTCTCCACAACTGCTACTGCTACAATAACTGCACAGAGTCTAACTTTCGATCCAACTTCTGTCTTCGACATTTCATCAAACACCTTGCATGCACTACAGCCAACTCCAAGTTTCGCTTCTATTTTGGACTCTTCTCCATCTAATTTCACTAATTACACAAGGAATTCAACTTTTCCAAGCTTAAGATCACTCCATGAGAATCTCCAGCTTCCGTTATTCTCCGGCGGAACCTCCGCCATGCACGGCGGATTTTCTAATCCGATGGTTAATTGGACCGTGCCGGAGACTCAGAAAGTTGAACAGTCTGAACTTGACTGTATGTGGAGCTACTGAATTAGCAAGTGCTTAATTTTAATTAAGACGTTAATTAATTAAGTAATCAGTGTGTTAATCTTTGCTTTTTGTTTAGTATGTACTGCGTGTGGCAGCTTTAGTGGTATTGTCACTTTGTTGTAAACGTTCAGCGTAATTTAATGCATGCTATTATTTCTCG

>*Ss*CUC2 |Sspon.07G0020380-1A|*Saccharum spontaneum*

ATGGAGCGGTTCGGCGTGCTGGGCACGCGGCTGGGCCTGGACGGCGTCGTCGGCGGCGGCGGAGGCGAGCTGCCGCCGGGGTTCCGGTTCCACCCGACGGACGAGGAGCTCATCACCTACTACCTCCTCCGCAAGGCCGTGGACGGCAGCTTCTGCGGCCGCGCCATCGCCGAGATCGACCTCAACAAGTGCGAGCCATGGGAGCTCCCGGGTGCGTCACGATCCAGTTGCAGTCCTGCTGCATCGACCATTGATAGATCGCTATTCTCATGCATGCTGATGCTGATCACGTACACCATTGCTGATTGCATTGCCCCCACGCACGCACAGACAAGGCGAAGATGGGGGAGAGGGAGTGGTACTTCTACAGCCTCCGCGACCGCAAGTACCCGACGGGCCTGCGCACCAACCGCGCCACGCTGGCCGGCTACTGGAAGGCCACCGGCAAGGACCGCGAGATCCGCAGCGCCCGCTCCGGCGCGCTGGTGGGCATGAAGAAGACGCTCGTCTTCTACCGCGGCCGCGCCCCGAAGGGACAGAAGACGCACTGGGTCATGCACGAGTACCGCCTCGAGGGCACCTACGCCTACCATTTTCTCCACAGCTCCACAAGGGTAACTTTAATTTGAGCGACCATTTTTATTCTAGCGTAAACTTTAATTTGCAAATTATTCTTTTCCTGGCTTTGTATTGTTCATTTTGGTCCAAAAAAATGCTACTAGTATATACTCTTATTTTTTATGTTCTCTGGAATTGAAGTTCTTTACGCATGCATGTAAAAATTTGAAAATAAAGTTTTTGTACACTACGCATTCCACAAAACTACAGATACTCCTACGTAGAAGTAGTACAGCACTACAGACACAAGGTAATGATGATGGGAGGGCAAAAGGGTGGTCATAGGCTCACTCGCATGGCTAGCTACGAGTAGGACAGGAGCAGCTGCTGCACAGTGTGCCACAGTGCACAGGGGCAGACCTGTACTGTACGTGCCCACGTTTTCCCGGCCTCGCATCACGGCATCAGAGGCATGCATCAGTTGAGTGCATGCGTGTAGGATACGTACTACTGCTGCTGCTGGCTTCTGCAGCTCGCTCTGCTGCTACATTACTGTACGTGCGTGGATTGGCAAGGCTGGTTCTAAAAATTTTTGCAAAAAAACAGTGGATTCTTCGTCACATCGAATCTTTAGATGCATATATGAAACATTAAATATAGATAAAAAATAAAACTAATTACACAGTTTAGACGAAAATGGACGAGACGAATATTTTAAGCCTAATTAAATCATGATTGGACACTAATTACCAAATAACAACGAAAAGTGCTATAGTTGCATTTTATCAAAAATTTTGCCAACTAAACGAGGCCCAAAAGCCAGCCCAGGAGGGGATTAATCGCAGCGCAGCGCAGGAAAAGATAGGGCACAGGGATTCGAATCTTTTCTCGGCCAGTCCCGACGATGCGAGCGTATCAGCAATGCGTAAATGCTCAGCTTGTTTGTTTTTTTACTAGTACTACTGCTGCTACGCTAGCACTTTCTGAACTTTATATTTTGTTCGTGGGTAAAATCTACTGTATTTTGTATATTCATGTTTCCCAATTTGTGTCAGCAAAGTGTTTTCCCATTTTGGCATAAAATAAAATCAATTGTATTGTACTATTCATGTCTCAAAGCCATTTTCTACTTACTCCGTATTAATTATGTTTGTACTAGTACTTTTTCTAATTATGAGCAGTAAAAGAAAGGCACCTGAACTGTAGCATTGTTTCTTTAAATAACACATGATGGACCTTTTTCAGGATGAGTGGGTGATCGCCAGGGTGTTCCAGAAGCCCGGCGAGGTCCCACCGGCCCGCAAGCACCACCGCCTCGGCGGCCTCAGCAGCGCCGGCGGCGGCGAGTCCTGCTTCTCGGACTCCACCTCGGCCTCCATCGGCGGAGGCGGCGGCGGCGCATCGGCGTCGTCCGCGCCTCGCCCGCTGCCGCTCACGGTCACGGACGCCTCCTCGCTGTCGCTGTTCGCGTCGGCCGCCGCGGCCAATGCCGCCGACGGCGACAGCAGCTCCTACTGCGGCGGAGCCGCGAACAACGCCAACAATGGCAACAACCTGGTCACCGGCCGTGAGCTCGTGCCCTGCTTCTCCACTAGCACCACCACCGGCGCCGGCGGCCTGGATGCCGCCGCGCTCGGCATCGGGCAGCCGTACAACGCAGCAGTCCCGCTGCCGCTGGCCTTCGAGCCGCCGCCGCCGACTCCGGCCTTCTTCCCGAACCTGCGTTCGTCCCTGCAGCTGCAGGTGCAGCAGGACAACAACCTCGAGCTGCCACTGTTCCTCTCGGCAGCCGGCGGCCTGTCCGCTGCGACGCTGGGAATGGGGTCGATGGGCGGCGGGGCTCTCCACCACTGGCCCCTCGCCGGCATGGAGGTCAAGGTCGAGGGCCGCTCCGCGCCGCCGCAGATGGCTGTCGGCCCCGGCCAGCTCGATGGCGCCTTCGGCTGGGGCTACTAG

>*Ac*CUC2 |Aco020094|*Ananas comosus*

ATGGAAAGCTACGCGGCGCAGCACCACCGTTTCGACAGCGGCGATGCGCAGCTCCCGCCGGGGTTCCGCTTCCACCCGACGGACGAAGAGCTGATCACTTACTACCTCCTGAAGAAGGTCCTCGACGGCGGCTTCACCGGTCGGGCCATCGCCGAGATCGACCTCAACAAATGCGAGCCCTGGGAGCTCCCTGGTCCGTAATAACACTAACCCTAACCTGCTCATCGCCGCCGACCGCATCTATCTCTCACTGCTGCATTACTTTTCAATTATTTGTTCTCCCGCAGAAAAGGCCAAGATGGGGGAGAAAGAGTGGTACTTCTTCAGCCTCCGCGACCGCAAATACCCGACGGGGCTGCGGACGAACCGGGCGACGGAGGCCGGGTACTGGAAGGCGACGGGGAAGGACAGGGAGATATTCAGCTCCCGCACGGGCTCGCTCGTCGGGATGAAGAAGACACTGGTGTTCTACCGAGGGAGGGCCCCCAAGGGGGAGAAGAGCAACTGGGTCATGCACGAGTATCGCCTCGATGGGAAGTTCGCCTACCACTTTCTCTCCAGATCCTCCAAGGTACATATATATATTATCATTACGCTTATTCAACCGTTACTTTGTATCTTATTATTTATCGGTTTAATTTTTTTGTTTTTGACACAACATGGTATCAGAAAAGTCGGATCCTGCATCAATATTAGTTTATTTAGTTATTACATGTATCTCATTATTTATCATGGTTTTAATTTTCTGATTTTAACATGATATGAAAAAAAAGCTGACTCTTATTAGTCATTCTTTTGTAATCCAAATGCGCTGAATCTTTTCGCGTCCTTAGTGTGTGTGGTATTTATCGGGAAATGGTTAGGGTGTCGTAACATATTGTGTACGTTGATGCGTCTGTCAACATTTGATTGAGCTTGAGGTGAATGGACGCACTAATAAATTAATCTTTTAAAAAATTCTATTTTAATTTTATATACCATTTTAAATGAAAACTTGAAAGAAATTAGATTGTTGGTATAGGCTATATATTAATACTCTTTTTTTAATGAGCATTAGTCCAACCAAGGAGGAACTATTTTTATGACACACTCAGGTTGCGTTTGTGAACCAATTGAAGCGCTAACACATTAACTACATATATAAAAAGCATAACATTTTGGACTAACTTAAGTAAACACTGTTTATTTCATCCTGGGAGAATGTATGAAGAGTAGAGAGGAATCCTTCGAATTATTGTTAGGGTGTTTCCTTGTAATTTCCTTTTTGGTGATTGGTTTTATTTCAAAGCTTCTGTGATGATCTGAGGTATAATGATGTTTCCCTTTCTTTAGTTGAGCGGTATCTTGGTTTGTTGTGTTGGAGGTTAAAGCGTCTTTATGATGCGATTTCCTTCGTAAAGGGTGAAAGATTTTCTTACTGAAAAGCTACTCATTTCGTTTGGATTAATTGGGAAAAAGCGAGACAAAATTGTACACGCATCAAAAGCCCTTGTGCTGCTATCGTAGCTTGTCCTCTCATTTGTCGCATCTCTATCGTGTGCACAGCTCTCTAATTATCAATCAACTAAAAATTATTATTATTCTTTGTCCTTATTAAGATCCTTTCATCTACGCCTTTTTAAATCCTAAGCTTTTGAAATCACTAAAGTTTAACGTTAGAACCATTTGAAATCGAACAAAATATGGGAGGGATTGGGTTAACGGAGATGTTTTGTTCCTGCATGCGCAGGACGAATGGGTGGTTTCTCGGGTGTTCCAGAAGATTGGCGGCGGCAAGAAGACGCGCCTCGGCCTGGCTGGCCCTTCCAATTCTGATGCCGCAGGCGGTGGCGTCGGCTCGCAGTCCTCCAGCTCGCTCCCGCTGCTGCTCGATTCCTCGCCCTTTGCCGGCGCCGCCTCCTCATTCGCCTCTGCAGATCGCGAGAGCTGCTCCTACGAGAGCACTGATAGGGAGCCCGTGCCCTGCTTCTCCACCACCGCATCCCACCTCCTCGGCAACGAGGCCACACCGCCACCGCTCTTCGGCCGGGTGGGCACCACTGCCGCTGCTACCGCTGCTACTAATGTTAACGTTGGGTTGGCGTTTCCGTGCCTCCGCTCGCTCCAGGAGAACCTCCAGCTCCCGTTCTTCCTCTCCGGCCTCGCGCCGCCGCTGCCGCCGCTGCCCGGTTCTGGCCGACCTCGAGCGGAAGGCGGAGCTGGGCAGCAGGGTCCCCCACCAGATGATGATGCCGGTGGGGTCCACCGAGCTGGATTGCCTCTGGACGTTCTAGCTTGTTTAACAACCGATCTCGTCCTTCCAACGCATGCTATATGTTTGACTTATGATTACGACTAA

>*Os*CUC2 |LOC_Os06g23650|*Oryza sativa*

ATGGAGCGGTGCAGCGTGCTGGGGCTGGGCGGTGGCGGGGGCGGGGGCGGGCGGCTGGACGGCGAGCTGCCGCCGGGGTTCCGGTTCCACCCGACGGACGAGGAGCTGATCACCTACTACCTGCTGCGGAAGGTGGTGGACGGGAGCTTCAACGGGCGCGCCATCGCGGAGATCGACCTGAACAAGTGCGAGCCGTGGGAGCTGCCGGAGAAGGCCAAGATGGGGGAGAAGGAGTGGTACTTCTACAGCCTCCGCGACCGCAAGTACCCCACGGGACTCCGCACCAACCGCGCCACGGGCGCCGGCTACTGGAAGGCCACCGGCAAGGACCGCGAGATCCGCAGCGCCCGCACCGGCGCCCTCGTCGGCATGAAGAAGACCCTCGTCTTCTACCGCGGCCGCGCCCCCAAGGGCCAGAAGACCCAGTGGGTCATGCACGAGTACCGCCTCGACGGCACCTACGCCTACCACTTCCTCTCCTCCTCCACCCGGGTCAGCACTTTCCCCATTTATTTTCTTCACCTTTTGCCCATTGTGTTGTTCACTTCATCCTTTGGAAACTTTCTGTTGTTGTTCTTGCTGCGGATGATGTGTGCAACATATGGTAGGAATAAGTCACGAGGGGTCTAGGGCACAATCGCTAGCTCTGAGCTTGCTTTTCTGGTGTGAAGTCATCAGCAGCGAGCTCCCTCCCTGCGCTGCTATGCTACTTCCTGTAGCAGCGCAGGGGTGCTGCGACGCAGATTGCTGCATACTGTACGTAGATATGGTAATCGGTCAGCTTAGCGCAGTGAAAGATCAGAGGCTGCAGCTTAGCAGGATACGAATCTTTTTTTGGCCTCATGGAAGCTTATATGCAATCCAACGCATGAATGCGTGGATACATGAGAGCACAAGGTGTAAATGCATACTGTACTCCTTACGTATTTGCAATTTGCATTTAGCAAAAATAGATGCCTTGGGCATACATCTGCGTTTGTCATTTGAGCGAGTAAACCAAAGAGGAACAAAGTTCGCCTTGTGCAACGTGCACGCGAAATCGTAGTACTGTACTGCTGCTACACAACCTTGTACTACCCTTTTTTTAGTTCATGATATATATACACAGTTAAATTCGTGTTGCTACACAACCTTGATTCTTAATTTCTCTGTGTTGATGTGTCGTGCAGGATGAGTGGGTGATAGCCAGGATCTTCACCAAGCCCGGCGTGTTCCCCGTCGTCCGCAAGGGCCGCCTCGGCATAAGTGGCGGCGGCGGCGACACCTCGTGCTTCTCGGACTCCACCTCCGCCTCCGTCGGCGGCGGGGGCGGCACCTCCGCCTCGTCGGCGCTGCGCGCGCCGCTGGCTGAGGCCTCGCTGTTCGCCGCCGCCGCGGCGCCAGCCGTTGACGGCGCCGACAGCAGCAACTACGGCGGGGGCGGCGGCGCCGGCAGCGCCACCGCCACCGCCAACTTGGTCACCGGCCTTGAGCTCGTGCCCTGCTTCTCCACCACAGCCCACATGGATGCCTCGTTCGGCACCGGGCAGTACAACCCGGCCCCGCTGGCCGTCGAGCCGCCGCCGCCGCCGCCGGCCTTCTTCCCGAGCCTCCGCTCGCTGCAGGAGAACCTGCAGCTGCCGCTGTTCCTCTCCGGCGGCATGCAGGCGGGCGTGTCGTCGCAGCCGCTCAGCGGCGGCGGGGCCTTCCACTGGCAGTCCGGCATGGACGTCAAGGTCGAGGGCGCCGTCGGCCGCGCGCCGCCGCAGATGGCCGTTGGCCCCGGCCAGCTCGACGGCGCCTTTGCATGGGGCTTCTAG

>*Bd*CUC2 |Bradi1g41712 |*Brachypodium distachyon*

AACCAGAACCAGACACCCAGCAGTATAAAGCAAGCGCTTGCCCAAGAGCAGCGACTAGCGAGGCAGCAGCCTACTCCACACTTGGTGCTCTTCCATCCTCTCAGTCGCCCCCACGAGGTAGAGTAATATTAATGAAACCCTCCCACTAGTACCAGCTACTGATCCTTCCATTCCTTTGGTTCTTGTGCCGTTCTGTGTGCGTGTGCGTCTTGATCGCTCAGTTCATCAGTTCGTTCCTTGTGTAGCTTCCAGCATTAACTAATGTACGTCGTTTTCTTTCTTGGCTCAGTGGTGTCGAGATCGTCGGTGGGGATCGATGGAGCGGTACGGGCTGCTGGGCACGCGTGAGGAGGAGCTGCCGCCGGGGTTCCGTTTCCACCCGACGGACGAGGAGCTCATCAGCTACTACCTCGCGCGCAAGGTGGCGGACGTGAACTTCTCGGGCGCCCGCGCCATCGCGGAGATCGACCTCAACAAGTGCGAGCCGTGGGAGCTCCCGGACAAGGCCAAGATGGGGGAGAAGGAGTGGTACTTCTACAGCCTCCGCGACCGCAAGTACCCGACGGGGCTGCGCACCAACCGCGCCACGGGGGCCGGATACTGGAAGGCCACGGGGAAAGACCGCGAGATCCGCAGCGCGCGCACCGGCGCACTCGTCGGCATGAAGAAGACCTTGGTGTTTTACCGTGGACGCGCCCCCAAGGGGGCCAAGACCCAGTGGGTCATGCACGAGTTCAGGCTCGACGGCAACTGCGCCTACCACTTCTTCTCCAACAACAACGCCACAAGGGTATTACTTCATCATTTTCTTTAGCTTCATCTTTCTTTGCTTCTCTTCCATGCCGTTCTCCTAGCTAGTACGTATATACTGTTGCATTTTTCTGATTCTAGTTTTAGGATTTTCCTTATCAATGCCGGGCTTTTAAAATCTCTTCTTTCCTTTGGGGAAGTACTCCAGTCCAGTAGTACTAGACTAGTCGCGATGATGAGCTTTACTCTTGTGTGAAGTCATGGCTACGGGCTATAGCCAGTGGCGAGTAAAATAGCAGAGAGCAGGAGCCTCTGTATAATCTCTGTACGTGTGTGCAGATTACATGCGTAGATATGGCAAACGAGAGGCTCAAGTGCAGAGAAAGATTGGCTGCAGCGATACGAATCTTTTCTTGCTCAGTGATCTCAATGCATGCATGCGTATGCATGCACAAGTTACTATATTCTCCCAGATGCGTAAATGCATGCCCATTTTGAATTACTAGAGTATCATTTTTTTACTTCGTCTTGTGGAACAAATCGGTCTCCTGGATCGGACAGTCTTTTTTCTTGTTTTTTTTTTGTGTTCACTTTGAGAAAGCGTAGCTAGCGATCGACTTGCTTCGCAGCAATGTTTTTCCGTGTTTCCGGGGTACATTGATTTCGTCATGTGAAAATGCTATTACTGCTCCTACCTAAACAAACATTTACTTAATTTCAAATAAAGGCGTTTCCTTTTCAGAATAGAAAAACTAAAGAACATACACCTGAAAGCAGCTCGCAGCAGTATCCGTCGGCTGTAGCAGCCATATTGATTAAACAACGTTTTTGCTTACACACGTAGTACCCATTTCGATGCTGACCAATGTGTATGTATCCAGGACGAGTGGGTGATAGCCAAGATCTTCGTGAAGCCCGGCGCGCTCCCCGCCGCCCGCAACAAGCTCGCCCGCTTCGGCCTGCAGGGCAGCACCGGCGGCGCCGACACGTCCTGCTTCTCCGACTCCACCACCTCCGTCTCCATCGGCTGCGGCGGCGGGGGCGGGGATACCACCACCAACACCAGCTCGTTATTCGCGGCCGCGGCCGACGGCGAGAGCAGCTCCTGCGGCGGCGGCAACAACAACAACTGCGGCCGTGAGCTCGTGCCCTGCTTCTCCACTGGCGCCCACATGGACGCCACCCTCCTCGGCATCGGCCAGTACGACCCGGCCCCGCTGGCCATGGAGCAGCCGCCGGCCTTGTACCAGCTGAGCGCGGCCCGCTCTGTGCAGGACAACCTCCTGTTCCTCTCCGGCGGCGGCCTGCAGTCCGGCCTAGTGTCCCCGCTTGGCGTCGGCGGAGGGGCTTTTCAGTACTGGCCGACGTCGTCCGGCTACGACATGAAGCCCCCGCAGATGGCCGTCGGCCCCGGCCAGCTTGACGGCTCCTTCGGCTGGGGCTTCTAGGCTCATTGCCTGGTTCTGTGCTAAGTATGTCTCCATCTGCTATCTTATCAGTATATGCATGTGTTAGCCAAGTACGTAGGTGGTGTTATCATGCCGTAGTTTAAGTTTGGGTAATGCTCTCTCTGTGGTCGATCGATCGTGGCTGCCGGATCGATGATCACGACTTCGTAATATGGATCACCAGCTCGCTATTTAAGTCGTTGCCTAGTTAATTTATGTATTATTAGTCCGTGCATGTGGTATGTACTAGTATCAGACTACTATCAGTACCGATCGACGTGCACCGCGCGCCGGTTTGTTACAGGACGTGTAGTACTCCAGTATTAGACAATATTAATTAATGTAGCTCAAATGAGTGTTACGTGTTTAAGTGTTTGCGTCTGTGTTTTCTTAAAAAAAAAATGTCACATGTGTACCCGTATTCCTTCCACCGCGGTGGCATGCTTGCCTTTTTT

>*Zm*CUC2 |GRMZM2G139700_P01|*Zea mays*

ATGGAGAGGTTGGGCGTCGGCGTCGGCGTCGGCGAGCTGCCGCCGGGGTTCCGCTTCCACCCGACGGACGAGGAGCTGATCACCTACTACCTCCTCTGCAAGGCCGTGGACGGCGGCTTCTGCGGCGGCCGCGCCATCGCGGAGATCGACCTGAACAAGTGCGAGCCATGGGAGCTCCCGGGTGAGTGAGTGAGTGGGTTACATATACGTACATACTCTCGCTGGTGATGACCGTACCGATCACCCGACTATGATGATGATGATGATGATGATGAGCAGACAAGGCGAAGATGGGGGAGAAGGAGTGGTACTTCTACTGCCTCCGCGACCGCAAGTACCCGACGGGCCTGCGCACCAACCGCGCCACGGCGGCCGGCTACTGGAAGGCCACCGGCAAGGACCGCGAGGTCCGCAGCGGCCGCAGCGGCGCGCTGGTGGGCATGAAGAAGACGCTCGTCTTCTACCGGGGCCGCGCCCCCAGGGGCCAGAAGACGCGCTGGGTCATGCACGAGTACCGCCTCGACGGCACCTACGCCTACCATTTCCTTCCCGGCTCTACGAGGGTTAGTTAGATCAGATCATCTTGTGCTCTACCATACCAGCATTTGTTTATGTTCTTTGCGCATGCGTGTTTTTGTTTTTTTTTTTAATTTTTGAAAATGAAGCTCACGTAGACCATGCACACACACTACGAATACTAGTAGGACAGGACAGCACTAGAGACAGAACGTAATGATGATGGGAGGGCAGCGCGTAGGACGACAGGAGTAGAGCTGCAGGGTGCTCTGCACTGCAGCTAGTAGCAGTCGAGTGCATGCGTGTGGGATACGACCATCTGCTTGCTCCTGCAACTCTGCTGCTACTGTGTCATTACGTGCAGTGCGTGGATATGGCAAAGCCCAAACCTGCCCCGGCTGGGAAGATAGAGCACAGGGATTCGAATCTTTTCTCGGCCAGTGCCGACGATGATGCTATCAGCGTGCTCGATCTCACCTTGTTTGTTTGTTTCTGCTGCCACCTGCCATGGTAGCACTTTGATCGATTTCATGGTTCGATCAGAGAAGTACTAGCATTTTCCGCGGAGTACATTTTTACTTTATTAACTATCATCAGCAGTAAAAAGAAAGACACCTGAACTGTACAGCATTCACTGTCCGCCTTCCTCCAGGACGAGTGGGTGATCGCGAGGGTGTTCCAGAAGCCAGGCGAGGTCCCATGCGGCCGCAAGCACCGCCTGGGCGGCCCCAGCGCCGCCGCCGGCGAGTCCTGCTTCTCGGACTCCACCACCTCGGCCTCCATCGGCGGCGGCGGCGGAGGAGGAGCGTCCGCGTCGTCTCGCCCGCTGCTCACCGTCACGGACACTTCCTCGCCGTCGCTGTTCGTGGCCAACGCGAACGCCGCCGCCAGCAACAACAACGGCAACCCGGTCACCGGGCGAGAGCTCGTGCCCTGCTTCTCCACTACCGCCAGTCCCCTGGAAGCCGCGGCGCTCGGCGTCGTCGGGCACCCGTACAACGCGGCCCCGCTGCGTCTGGGCTTGGACTTCGAGGCGCCGTCCCCGGGCTTCGTCGTCCCGAACCTGCGTTCCCTGCAAGTGCAGGACGACGGCGGCCTGCCGCTGTTCCTCTCGGCAGCAGCAGGCGGCGGCATGTCGTCCGCGACGCTGGGAATAATGGGGTCGCTGGGCGGGTCTCTCCACTGCCCGCCCCACGCCGGCATGGATGTCGTCAAGGTCGAGGGCCGCGCCGCGCCGCCGCAGATGGCTGTCGGCCCCGGCCTCCTCGATGGCGCCTTCGCCTGGGGCTTCTAG

>*Hv*CUC2 |MLOC_65286|*Hordeum vulgare*

CCTCCCCCAACCACGCACGCGGCCGGCCAGCGGCACTATAAAACAAGGGCGCGCCTCCACTACCAGCGAGACAGCCGCTCCCCACCACCACTCGCTCTTCGATCACTTCTCTCGCCACAGGTACTGATCCTTCCATTCCATCCCATTCCTTTGGCCCTTGCGCTGCTCGCTCGATCGTTCAGCCGGGTCCGTTCGTTCTGCAGCTTCCGGCATTAATGGTGCACGCACGCTTTGCTTGCAGGTCAGTGCGAGGGCGCGGCCGGGACCGAGATGGAGCGGTACGGTTCTCTGGGCATGCGGCTGGACGGCATCGGCGGCGGGGGCGGCGAGCTGCCGCCCGGGTTCCGCTTCCACCCGACGGACGAGGAGCTCATCACCTACTACCTCCTCCGCAAGGTGGTTGACTGCGGCTTCTCCGGCGCCCGCGCCATCGCCGAGATCGACCTCAACAAGTGCGAGCCGTGGGAGCTGCAGGACAAGGCCTGCAAGGCCACGGCGGAGAAGGAGTGGTACTTCTACAGCCTCCGCGACCGCAAGTACCCCACGGGCCTGCGCACCAACCGCGCCACCGGCGCCGGCTACTGGAAGGCCACCGGCAAGGACCGCGAGATCCGCAGCGCCCGCAACGGCGCGCTCGTCGGCATGAAGAAGACGCTCGTCTTCTACCGGGGCCGCGCCCCCAAGGGCCAGAAGACCCAGTGGGTCATGCACGAGTTCCGCCTCGAGGGCGTCTACGCCTACCACTTCCTGCCCAACAACACCACAAGGGTCAGCTACATTCCATCCTTATTCCCCTCACTCATCTTCTTTACTACACTACTAGCATTGATCTGCATTTTGCATCTCCTATGCCGCCTGATTTTTAGTCTTGTTCTCTGGGAAGTAGTGGTAGCTAGTACCGGCGGCGATGATGCAATGGTGGAAAGTCACGGGGGGTTTAGGGCAGATTGCTATACTTGTGATGTGAGCTTACTTTTCTCATGTGAAGTCATGGCTACGAGCTAGAAGGATAAGCTAAGCTAGTGACGAGTAGAGGAGAGCTGCTAGCTGGAGCAGCAGGCTCTGTACGTACGTGTTCCTGCTGCAATGCAGATTGCATTGCGTGGATATGGTAAGCGAGGGGCTTAAGTGCAGTGAAAGATTTGCAGCAGAGGGATACGAATCTTTTCCTCGCCCCTGCCCGCGATGCTGCGAGTGCATGAGCATGCATGCACAGATTACCATATTTCTACAGATCCGTAAATGCATGCCATGTTTGGGTGTTTTACTGCGTTCTCTTGAACCCATTCATCCCATGCATCAAATAGTTATCATTTTTACCTTGTCTTTTTTACGCAGAGATGAGGAAGTCTACCTTAAGTTGCTTCATAGCCGTTGTTTTAATGTTTTCCGCTCGTTGCGTCATGTAAAAATGCTAGGTGGTTGGATTTGATCTTAATGAAAACGGGGAATGCTTTTTCCCACTGCGTTAATATGTGGACAAATAGAGATTGGAAAATGTTAGCCTAAATAAGGAGTACTTCAATCCGTCCCTAAATAAATGTTTTAACTTTATATTAAATTTAAGACATTTATTTTAAAAATGAGGAAGCAGATAACTTTTGAATGATGTGACCTTTTCATAATAAAATATTGGTCTTGTATAGTGTATGTCTCATAGTGTATACGTCGTATAGCATTCAACTACGTCGTCAACGGTGTGATATTCAGCATTTTTTGCTGACACGTTGGTGTATATATCCAGGATGAGTGGGTGATCGCGAAGATCTTCGTGAAGCCCGGCGCGGCGCCCCCCTCCCGCAAGGCTCGCTACGGACTCAGCAGCGCCGGCGACACGTCGTGCTTCTCCGACTCCACGTCCGTCTCCATCGGCGGCGGGGGCGGCGCCTCCGCCTCGTCCGCGCCGCGCCAGCAGCTCCCGGATACCAGCTCGCTGTTGGCCGCGGCTCACGCTGCCGCTGACGGCGAGAGCAGCTCCTACGGTGCCACCGGCAACAACAACAACGCGGCCGGCAACTGCCGTGAGCTCGTGCCCTGCTTCTCCACCGCCCAGATGGATGCCACCCTCCTCGGCATCGGGCAGTACGAACCGGCTTCGCTCGCCGTCGAGCAGCCGTTGGCCTTCTTCCAGGGCCCCCGCCTGCACCAGGCGGCGGACAACCTCAGCCTGCCGATGTTCCTCCCCGGCGGGCTGCAGTCCGGCGTCTCCCCGCTCGGCATGGGCGGAGGGGCCTTCCAGCACTGGCCGTCCTCCGGATACGAGGTGAAGCTGGAGGGCAGCCGCGCGCCGCCGCAGATGGCCGTGGGACCTGGCCAGCTTGACGGCGCCTACGGCTGGGGCTTCTAGACCAGTATGCATGCATGCCCTTAATCGTGTGCTGATTATGCTCTCTCATTCCAGCTTGCTTATCAGTATGCTTAGACGCATGTGTCCTTTGCTAAGTAGCTAGCTATATCTCGATCATTATGCTGCAGTAAGTTTCGATTATTAATTCATGCTCTCTGATCGTCGCGTGTTACTACTGCAACAGCAGGGCACACGACTTCACGAGCAAAGTACTGGATCACTAGCTAAATTACCTAGCTTAGATTATTAGCTAGTCGTATGTTGTTCTATTAGTATGTATTGTTAGTCGTATGTTGTATCAGACTATCAGTAACTCGATCGAGATGTGTACCGATTCTACTGTATCAGACTACTAATTAGTTGAAATCAATGTATGTGTATCTAAATTGTC

>*At*CUC3 |AT1G76420|*Arabidopsis thaliana*

ACTTCCCATCACCTTTTGTATCATTGCTATCTTTGTTCTTCCTTCTGGTGTCTCTTCAGTGTAACAAAAAGTCTCTTTTTCAGTTATATTAAGTAAAAGATGATGCTTGCGGTGGAAGATGTGTTAAGCGAACTCGCCGGAGAAGAAAGGAACGAGAGAGGATTGCCACCTGGCTTCCGGTTTCACCCGACGGACGAAGAGCTCATTACCTTCTACTTAGCTTCCAAAATCTTCCATGGTGGTCTCTCCGGCATTCACATTTCCGAAGTTGATCTCAACCGCTGTGAACCTTGGGAGCTACCAGGTAATATATTATACATTTTGTCTCTTCTTTACCTCATAAGTTTTATTTTGGAATCTTGTTTTTAGTTGAATATTTTGATAAATCTTTTAACAAGAAAAAAGTGTAACGTTTTGAAATTGTTGTTATATGAAGAAATGGCGAAGATGGGAGAGAGAGAGTGGTACTTTTATAGTCTAAGGGACAGGAAATATCCGACAGGTTTGAGGACTAACAGAGCAACTACTGCTGGATACTGGAAAGCTACCGGCAAAGATAAGGAAGTCTTCTCCGGCGGAGGAGGACAGCTTGTTGGGATGAAGAAGACGTTGGTGTTCTACAAAGGTAGGGCTCCACGTGGCCTCAAGACTAAGTGGGTCATGCATGAGTATCGCCTCGAAAACGACCATTCACACCGCCACACGTGTAAGGTAAATTAACACTCTTTTACTCTCACTCGTGCGTCTCACTCTCACTCGTGCATCTCTTTCTTTAAAAAAATTTGTATTTTTTCTCTTGTCAATTTATGAGTTGACTTTTTGAAACCAAAAATTTTATACTACTAGTGTGTGAAACTAGTAATTTAAACCCTGTTACTTTTATTCCCACACGTCCATTTCTTATAACATTAAATTCTTTTTAACTTTGCGAATGTAATAGAAATTTGATGTTTTTGTTCTCCATTAATGTGTTAACTTTTTAAATCCACAAGTTGAAAAGAAAGGTTTAACGAAATATTTAACTTTTTTTTTCCCATTTCATGTGTTTTGTTTCAATAGTATTGTCACTATGTTGTTTGGCTTAGTATGGGGAGGACAGGGGAGAGAGCAGTTTAGATGTGATAGATTTGATATAAACCTGTACGTGTGGTGTGGATGTCAACACCAAACACACCTTGCCGTTTTCTGTTCACCGATACACTGTTGTGTCGTTTTCCATCACTATTGCTTTCTTTTGTATCCCACCTTTCTAATTACATTTATCATTATCAGAAATCAATTTGTCAGGCTCACATTTCAAACAAGATCTTAAGGTGTAGGCATTATATAATGTGTAATAACATGTTTTGGTTTTTCATTTCTTTTTGGTGGAAATTCACAGGAGGAATGGGTGATTTGCAGAGTGTTCAATAAAACAGGAGACAGAAAAAATGTTGGATTAATCCATAACCAAATCAGCTACCTTCATAACCATTCACTCTCAACAACACATCATCATCATCATGAAGCCTTACCTTTGCTTATAGAACCTTCCAACAAAACCCTAACCAACTTCCCATCACTACTCTACGATGATCCACACCAAAACTACAATAATAACAACTTCCTTCATGGATCATCAGGCCACAACATCGACGAGCTCAAAGCCTTAATCAACCCTGTCGTCTCTCAGCTCAACGGTATCATCTTTCCTTCAGGGAACAACAACAACGACGAAGACGACTTCGACTTTAACCTCGGCGTGAAAACAGAGCAGTCTTCGAACGGTAACGAAATTGACGTACGAGATTACTTGGAGAACCCTCTGTTTCAGGAAGCGAGTTATGGTCTGTTGGGTTTTTCGTCTTCTCCTGGACCTCTTCACATGCTACTAGATTCTCCATGTCCTTTAGGATTCCAGCTGTAG

>*Br*CUC3 |Brara.G03438|*Brassica rapa*

ATGATGCTTGCGGTGGAAGATGTGTTGAGTGAGCTCGCCGGAGAAGAAAGAAACGACAGAGGTTTACCACCTGGCTTCCGGTTTCACCCGACGGACGAAGAGCTCATAACTTTCTACTTAGCCTCTAAAGTCTTCCATGGAGGTCTATGTGGCATTCACATTGCCGAAGTTGACCTCAATCGCTGCGAACCCTGGGAACTCCCTGGTATGTAATATATATTTTTTTTGTAAGAATGCTAAGATTTCTATCATTTTCAAAAATATATCTTAATATTTTATCTTTTTGTATGAACGTCAATATTTATACCCTTTTCAAGAATACATCTTTATATATTCGTTTCTCTATAGTTTTGTTTCCCTTTTGTTTTCTGAATCTTTTTTTAACTTTCATGTGTTTTCTTTTGTGTTCTCAAGAAATGGCCAAGATGGGAGAGAGAGAGTGGTACTTTTACAGTCTAAGGGATAGAAAATATCCCACAGGGCTAAGGACTAACAGAGCCACTACCGCTGGATACTGGAAGGCTACCGGAAAAGATAAGGAGGTCTTTGCCGGCGGTGGCGGCGGTGGAGGAGCACTTGTCGGAATGAAGAAGACCCTTGTGTTCTACAAGGGTAGGGCTCCACGAGGCCTCAAGACTAAGTGGGTCATGCATGAGTATCGCCTCGAAACTGACCTTTCTCACCGCCACACGTGTAAGGTAAATGATAACTGTGTTACTTTAGCCCTAAACATGCGTCTCTTAATACTTTTAAACTCTACCAGATTAAACGAATTTAATTGCGACATTATTATTTTTATAAGTTGACTTTTTGAAACTAAAACTTAAATATAAAATATTAACTCATTTAATATTTCACCTTCGTTCTGTAACTTTACTCCCACCCGTGCGTCTCTTAAACTTTTTTAATAGTACTAGAGTTTTTTTCCGCGCTTCGCGCGGATTGTATCTTATAAATTTATTTTATTTGTaatattatttgtcggttttttcttttacattaacttcttgtttttttgatgttattttttcttaatttaaatttatatgtttataatttttcatttttcttgttaaaaatagagaattatattttttattgatggttttttgtatgtgacataaaatttttgaaattttaaaataatgttatatatagtacgattaacacattaaagaagagaaacatattcaggcacattttacacatgttttatatgcataattttaaacattatatatgtatatattataagtttgaaacatgtaaatgctttctaaagctaaatacttgttctgagtttacataacttatcgaaagttttatctctttttaaattcaaatcacagaaaaaatatatcgaaaagtcagtatagatgagttttttgggcttttaaatcaacactgaaaaattacatgaatcagataACAACAGTTTTATAAACAACTGGATAAAATTTGACCGAGCCAAAAAATTTCACACAATATGTTCTCTCTTCTTCAAATTGCAAAGAGCCTATAGGCACAAGAAAAAAAATCATAACNNNNNNNNNNNNNNNNNNNNNNNNNNNNNNNNNNNNNNNNNNNNNNNNNNNNNNNNNNNNNNNNNNNNNNNNNNNNNNNNNNNNNNNNNNNNNNNNNNNNNNNNNNNNNNNNNNNNNNNNNNNNNNNNNNNNNNNNNNNNNNNNNNNNNNNNNNNNNNNNNNNNNNNNNNNNNNNNNNNNNNNNNNNNNNNNNNNNNNNNNNNNNNNNNNNNNNNNNNNNNNNNNNNNNNNNNNNNNNNNNNNNNNNNNNNNNNNNNNNNNNNNNNNNNNNNNNNNNNNNNNNNNNNNNNNNNNNNNNNNNNNNNNNNNNNNNNNNNNNNNNNNNNNNNNNNNNNNNNNNNNNNNNNNNNNNNNNNNNNNNNNNNNNNNNNNNNNNNNNNNNNNNNNNNNNNNNNNNNNNNNNNNNNNNNNNNNNNNNNNNNNNNNNNNNNNNNNNNNNNNNNNNNNNNNNNNNNNNNNNNNNNNNNNNNNNNNNNNNNNNNNNNNNNNNNNNNNNNNNNNNNNNNNNNNNNNNNNNNNNNNNNNNNNNNNNNNNNNNNNNNNNNNNNNNNNNNNNNNNNNNNNNNNNNNNNNNNNNNNNNNNNNNNNNNNNNNNNNNNNNNNNNNNNNNNNNNNNNNNNNNNNNNNNNNNNNNNNNNNNNNNNNNNNNNNNNNNNNNNNNNNNNNNNNNNNNNNNNNNNNNNNNNNNNNNNNNNNNNNNNNNNNNNNNNNNNNNNNNNNNNNNNNNNNNNNNNNNNNNNNNNNNNNNNNNNNNNNNNNNNNNNNNNNNNNNNNNNNNNNNNNNNNNNNNNNNNNNNNNNNNNNNNNNNNNNNNNNNNNNNNNNNNNNNNNNNNNNNNNNNNNNNNNNNNNNNNNNNNNNNNNNNNNNNNNNNNNNNNNNNNNNNNNNNNNNNNNNNNNNNNNNNNNNNNNNNNNNNNNNNNNNNNNNNNNNNNNNNNNNNNNNNNNNNNNNNNNNNNNNNNNNNNNNNNNNNNNNNNNNNNNNNNNNNNNNNNNNNNNNNNNNNNNNNNNNNNNNNNNNNNNNNNNNNNNNNNNNNNNNNNNNNNNNNNNNNNNNNNNNNNNNNNNNNNNNNNNNNNNNNNNNNNNNNNNNNNNNNNNNNNNNNNNNNNNNNNNNNNNNNNNNNNNNNNNNNNNNNNNNNNNNNNNNNNNNNNNNNNNNNNNNNNNNNNNNNNNNNNNNNNNNNNNNNNNNNNNNNNNNNNNNNNNNNNNNNNNNNNNNNNNNNNNNNNNNNNNNNNNNNNNNNNNNNNNNNNNNNNNNNNNNNNNNNNNNNNNNNNNNNNNNNNNNNNNNNNNNNNNNNNNNNNNNNNNNNNNNNNNNNNNNNNNNNNNNNNNNNNNNNNNNNNNNNNNNNNNNNNNNNNNNNNNNNNNNNNNNNNNNNNNNNNNNNNNNNNNNNNNNNNNNNNNNNNNNNNNNNNNNNNNNNNNNNNNNNNNNNNNNNNNNNNNNNNNNNNNNNNNNNNNNNNNNNNNNNNNNNNNNNNNNNNNNNNNNNNNNNNNNNNNNNNNNNNNNNNNNNNNNNNNNNNNNNNNNNNNNNNNNNNNNNNNNNNNNNNNNNNNNNNNNNNNNNNNNNNNNNNNNNNNNNNNNNNNNNNNNNNNNNNNNNNNNNNNNNNNNNNNNNNNNNNNNNNNNNNNNNNNNNNNNNNNNNNNNNCTTGAAGTCTAACGATTAAAAATAGAACATAATTCAATAGATATATGATTATATTAATAAATTAGTTGTTACAAATTTGAAATTTCTAGAAATATCAAAAGTTTTATGTTAGTTAATtatcttttaaatgacatttatttctaattctttttggatgagaatattttggctgaggTGGATAGTCTCAAAAACCTTGAATTTAGTTCCTTTTATATAGTAGGATTATTTTTTGAGAAAAAAAAATATTACAAATTTTTAATATTACAGGTTTAACTTTATATTTCAGCATTACAATTTTTTTTATGAACTTCATCTAAAGAGTTGACTTTTAAAAACGATAAGCTGAAAAGAAAGGTTTAACTGAACTATTCACTTTTTCCCAAATTCATTTGTTACACTATTTCCTCAAAACTATTTTTTCAATAGTATTGTCATTATTTGGTTTAGCTAGTATGGGGTGGACAGGGGAGAGAGCAGTTTGAGATGTGATAGATTTGATATAAACCTGTACGTGTGGGTGTCAACACCAAACACCTTGCCGTTTTCTGTTCTTCCATGCTACACTGTTGTGTCGTTTTCATCACTATTGCTTTCTTCAATTAACTCCATGTATCGTCATAAATCAATTGGCCAGCTCGCATTTCAAACAAGATCTTTATCGATCTTGGTTACGTGTAATTAATACAATAATACAAATATGAAATATGTTTATTCTGAAGTAGCTATATACTTCGAAACATAATGTCTAATAGTAGCATTTTAATGTTTAATTTATGGTGGTGACACAGGAGGAATGGGTGATTTGCAGAGTGTTTAACAAAACAGGAGACAGAAAGAATGTTGGAatccataaccaAATCAGCTACCTCCATAACACTTCACTATCAACAACACATCAACAAC

ATAACCATTATCATCATCTTGAAATCTTGCCTCCTCTTCTTGAACCATCTAAAACCCTAACCAACTTTCCATCGCTACTCTACGATGATACCCACCAAAATTACAATAATAACCTACTCCATGGATCATCAGCCCACAACGTTGACGAGTTCAAAACCCTAATCAACCCAGCCGTGTCTCAGCTCAACGGAGTCATTTTCTCTCCAGAAAACAGCAACTACAACAACGAGGACGACAACAACTTTGGCGTTAAGACAGAGCAATATTCGAACGGTGGCAATAACGATCTTGATGTGCGAGACTACTTAGACAACCCTTTCTGCCAGGAAGCGGGTTACGGTCTGTTGGGTCTTTCATCTTCTCCTGGACCTCTTATGCTATTAGATTCTCCATATGTCCTTTAG

>*Dc*CUC3 |DCAR_013939|*Daucus carota*

ATGTTGGCAATAGAAGAGATATTGTGTGAGCTGAATGGGAGTGAGATGAATGAGCAAGGCATGCCTCCAGGGTTCAGGTTTCACCCAACTGATGAAGAGCTCATCACATTCTACCTTGCTTCCAAGGTCTACAATGGCTCTTTCTGTGGTGTGGACATTGCTGAGGTTGATCTCAACAGATGCGAGCCCTGGGAACTCCCTGGTATGTTTCTGACTCCATGCATCTCAGCATATACAGTGTGTTAGTATACTGTTTAGTGTTGTATTATCTGTGCTTCATTGAATTATGTGAGGAGCATTTCAGAAATTTAATGCATGGTATTCGTTAGAGTACTGCCATACATAAATAGTGATGGAGCATGAATAATATCTCAAAAGCTTCCCCCAGCCATTCACTTTTGCCAGAAAGCAAAAGTATCCTCACGTTTGCAGCTAAATCATTACTAACCAACTGACAGATATATGAGTAGTATGTTTGATCTAGATAACATAACACACACAAACTAAGTTACTTTTAAGTACCTTTTCCTCCATAGTAGTACATTTTAAGTCCAAATGTAGGTCACGGTTACGGAATCAGGATTTCGAGACAGTCGGGCTAAAATTAAGTATGGCTAATTTTTGGGTGATACAGACATAGCGAAAATGGGGGAGAGAGAATGGTACTTTTTCAGCTTGAGAGACAGGAAATACCCAACAGGGCTGAGAACAAACCGAGCAACAGGAGCTGGGTACTGGAAAGCTACTGGGAAAGACAGAGAAGTTTACAGCAGCAGCGCCAATGGTGGTGCTGCTACACTTCTTGGGATGAAGAAGACTCTTGTTTTCTACAAAGGCAGAGCTCCCAGAGGTGAAAAGACCAAATGGGTCATGCATGAATATCGCCTTGACGGTGACTTCTCCTGCCGTCACACGTGTAAGGTAATTCCATAATTCCATAACAACTCTCGCACGCTTCAGCTTATAAGCCCTCCTTAAACTGTCCTTTTTGGGTTGACTTTTATGTTGCATTACCACTGTTTTAGTTGTAGCCTTGGAGCTATGCATATGATATTTGGCAAATTTTGCAGATATTAGGTAAGTTTGATATCGGATTCTTTGGTCGAAGAATATATTGAAATCACAAAACACTGCAACTCAGCACTATATGCAAAAGTCTTGCTGTGTTTCTTTACCTGCTGCTAGCTTCATTTTTGCTGTGGACATTATTTTTGGGGGAATGGGAATTTGTGATTTATATTGTTGGGATGTGACTGGGTGAATAGTGGGAAGGATTTAATTTGTGTTGTTTCCTTTAAAATTCAGATATTATTGCAAAATCACAAACTTATGCAGAGTCTAGTCACAGTCATGTTAAAGTGATTGAGGATTATGTGTGTCGTATTGCTTACAAATCACCATGTCAGTACGGATTTGTGGGTAACTTTAATTACCAACTCATATGTGAACGAAATTGATCGGATAGTCCAAGTGGTAAGGGTCTATCATCAGTTCTCCGGATCTCATGTTCGACTTGTAGTTATAATAGTGTTTTAATGATGACAGAACACCCAATAATGTGGCATTGTTTTCTTAATTCGAGTAAATTTTGAGTTAGATGACAATTTTTTGAGTCGATATTGCAGGAGGAATGGGTGATTTGCAGAATATTTCAGAAAATCGGAGAGAAAAAGAACGGAGGGCTTCTCCAGGGACAAAGCAGCAGCAGTAGCTACATGCAAGAGGCTTCAAACTCATCCTTGTCCCGTTTGTTTGATCAAAGTCTTAAATCAGGATCAGCTCTATCTTTACAACCTTACCATACACTCCAGTCCCTCCAAAACCAAAACCAAAACCGAAGCCAAGTACTTATAAACAACATCCCGCATGAAGCAGACCTTAAATCCCTCATAACCAACTCATCATCCTCATCTCTGGCAGTGTCACAAGCCAGCCCCTTCCCCGTAAACAATGAACTCAATGGCCTGCAAACATCATGTTCACCGCCAAAAACCAAAATGAAACAAGACCACAATCTTCTCAAAACTCTCCTCCCACACCAGGATTACTATTGTCCCAAGGAGCAAGAAGAAGCTCCTTTTCCTAAAATCTGCAAGACCGAGTCCAACTTTTCGCATTTCCAGTCCCCTCATTTCTCTACTCATCCCCACATTCCTAATTTCCGATTCCCAATTTCTACAACCGCAGAATATGACATGAACCTGGCTCATCAAATCACAAACACCCCCAACTACAAACAAAGCCCATTGCTTTTCAGGAGTTTAGATAGTGATACCAAGAATGTAATGGGGAATAATTGTGGAGTTGGCTTGGGTAGCTGTGGGTTCCCAGCTTATGGTACTGGAGACACTGAAATGTCAACTTCATCATCATCGTGTTCACGAGGACTACCTTTCAGCAGGGCCGGCTTTAAGCAGATGCTGCTGCTGGATCCTCCCACCAAGATGAGCGCAGGAGAATCTTGGCCTTTTCACTTTTAA

>*Eg*CUC3 |Eucgr.F03588|*Eucalyptus grandis*

ATGCTGGGAGTGGAGGATTTGCTCTGCGAGCTGCGCAGAGAAGAAGGGAACGAGCAAGGGCTACCGCCAGGTTTCAGGTTCCACCCCACTGACGAAGAGCTCATCACCTTCTACTTGGCTTCCAAGGTCTTCAATGGCGCTTTCTCTGGCCTCGACATCGCCGAGGTTGACCTCAACCGATGCGAGCCCTGGGATCTCCCTGGTACTTACATACAGACATACATATATACATACATACGTTGCTTGCTTTTCCCTCTCAGCTCTTTGGGTTAGCATACCCAGGGAAAAAAAATCCAGCGTTTTCAGATGTAATCTGTACATGAAGTCTGTGCTAGGGATCGGTAATTTTATATCTGTCGTCAGTAGTCTAGTAGAGTGATCAATATAGCAGATCGTGGTATATACCTGGGATCGGAGAGAGCTCAAAAGGGAGCGCAGTTTGCGCCTAGAAAAACGAAAGGAGCCAATGTATGCACAAAACACCAAGCTATCGCATTAATTTCGTCAGGGTATTCACTAGAGATGTTCCTACAGCAGTAATTGCTGCCGCTCTTGATTCGCTATTAGGCTTCAGCATGCATCCTCCATGCAAATAAAGATTTGTAGATTTACTTGCAATAAACCCCTGGAAGATTAAGTCAGCATCTCCACATCCATGAGGTTATTGTTTTGCTTTATCGTTCAAACCACTAGCTTTTACTCAGTGAATTTGTGTACTCTTGAAGCCTAGAGATGAAAATTAAGTCTTGGCAAGTTATGCACGCCTTAGCTACTAACATTATCAGTTTATCATCACTGTGAGAGATAAGAGAGAATGCTGCACTAACGTTCCAGGGCTTCTAAGAAAGTCCACACGAACTCTGGATGACTATGTAGTAATCATGTTCAGGCAGTACTTATTGTTGGGCGACTTTTTTCCGCCAAATTTAGCGTTGCGCTATCAATTTATTTACATTACTAGAAATGCCAAGGATTTTGCTAATCTTTATTTTAGAGACGAAAGTAGAGTCATTCACTTACTATTCTGAAAATTCATGCTTCGAAAACTGATCAGCCATTATTATTTGTTCTATCACTCACGGCATCGATGTTGTGTGAACCTATTTGTTTTCCAATAACGTTGAATGTTCGGGCCTATTAATGTTTCGCGAACACTCTCACCTAACAGAGGTCGCAAAGATGGGGGAGAGAGAGTGGTACTTCTTCAGCCTTCGAGACCGCAAGTACCCGACGGGGCTCCGGACGAACCGGGCCACTGGCGCTGGCTACTGGAAAGCCACGGGAAAGGACAGGGAGGTCCACAGCGCCGCCACCGGGGCGCTCCTCGGCATGAAGAAGACGCTCGTCTTCTACAGAGGCCGAGCTCCCCGCGGCGAGAAGACCAAGTGGGTCATGCACGAGTACCGCCTTGACGGCCACTTCTCCTGCCGTCACACGTGCAAGGTACGACTGTCCCTCCCCTCCTCGCACGTGCGGCCGTGTGGATCAAGCTACGGTTTTATCGCTCTTTTTACGTTTGTCCTTTTCTGGGTTGACTTGTATGGATCGATGAAGAACGATGAATTGATGGGTTTCACAATCTTTCACCCCCTCGGGGGATTTTGGATTTTGATATGATGGGTCTTGGTTTTCACAAACCCATAGGGACAATCTTCTGATGTTTCTTTTACCCGAGGTTAAAGGAAGTTTTTCACTATGCAGTAGCCTAATGAGTTTTGACAGAATATAATGAATGAGACGAAATTTTGTTGATGCGGAAACCGAAAACACATTTGCATTTTTCTAAGGCCCAGACGGTCATAAGAAAGGGAGTAAAACCATCAGGGCGCGCCAACAAAAGCATGTTTCAATTAGAGCGAAGAACTCAGTTTTGCATGCATGTTCTAATAATGGTTAGTCTCGTGTTAACATTATATTGCTCATATTTGGAAAAGTTACCCTTTAAGGATGGTGCGGAAGCAAATGAAGTTCTTTATTCCCAATCTGTAATCATATCATACGAATTAATTTCTGAAGCGGGAGTTGGTTTTTTTCCTTTTTTGGCAGGATGAATGGGTCATATGCAGGATATTCCACAAGACAATGGAGAAGAAGAGCCTGCTCTTCCAAGGCCAAAATTACATGCTTGAAGTTTGCTCACCCCCTAGCACTGGTTCGTTGCCTCAGCTTCTTGAAACCCCAATTACATCAACGCCACCACCAAATCATCTACTAGAGCACCAATCTCAGGCCAATAGTTACCAAAACCAAACCCAACCTTTTCTGATGCACCAAAGAGACATTGATTTCAAAACCCTAATGAGCAACCCATTTGTCCCGCTAGTGCCTGATCTTCAAGCACCCTCATTTTCACCCACAGTCACCACCACCACGCACAAAGACTCGTACAACAACACAGACACCAAAAACATCATCGTCACCCCATCACTAGTCTCGCCATCGTCGATGCTCTTCAAGTCCCTTCTCTCACATCAAGACCTTTTCACAGCTACTAATTTGAAGGAACACCAGCACCACACTGTGCTCAACCAGTGCAAGACAGAGACCAACTTTTCCCATTTCCAGCTAAACGTCGACGATGATAATGATGCCGATGGCTTGCACAGTGTTGTGGACAAGATTCACATTGTCCCATTTCATCAAAATCACCAAAGCCCCGTGCTTTTCGAGATGGACACTCACCCTACAACTACATCTTCTGTTCCAGATAATAATAATGCACCTCATGGCCACACTGTTCATCATGAGATGTCCACGTCAACAATGGCTTTCAACAGGGTCGGGCCGTCCTTTCAGATGCTGGTGGATCCTGCCTTCAGAGAATCCTGGCCGTTGGATCCTTGA

>*Gm*CUC3 |Glyma.06G014900|*Glycine max*

GTATCTGTATGTCCACGTTGAGGTTCATACGTATACCTGTTCACCTGTTGTGTTCGATCGATCTATTCTGCTCCTCCCCTCTTTTTCAGAACTCCCTTTAAAACATTCATTATATATATATCAATCACTCCAACAACCCCTTTACCTCATCCAATATTGTTTTCCTCTTTGCACAAAAAAGAAAAAGGGTCATGTTGGCAATGGAAGAACTACTGTATGAACTTAGTGATCATGAAAGGAGAAACGAGCAAGGTCTGCCACCGGGTTTCAGGTTTCACCCCACTGATGAAGAACTTGTAACCTTTTACTTGGCTTCAAAGGTCTTTAATGGCACCTTCTCTAACGTCAAGTTTGCTGAGGTTGACCTCAATAGATGTGAGCCTTGGGAACTTCCAGGTATCATATATATACATATAATCTCCCGTCCGAACCCTCACACACGTTGATTTCCATCAACCAAAATAAAAGAGAAAAAAAAATACTTACTAAATGGTTAGTGGTTACATTATAGAATTTTTTAGAGTCAGATCAGATGTATGTTATGTTATGTTATTTAGCTAGAATGTTTTCTCGTTGTTAGTTTCAATTCCTGCTGGCTCTGGCCTTATATTTAGGTTAATTAGTGAACCTAAGACATATTTTGTGGGCTATCTACTAGCATGTCACTTCAAATTTCTAATGGCGTCATCAGCTTCCCCGCTTTGGGTCTTCTTATTGTTTTTCCTTGTCCCTAATTTTAGACTGTGCTGAATGATCGAGAGTGGACAGTAACTTAATTCTAAGAAAGTTTGAACAAAATTTGAGCTACCCAGGCCCGCTTGCAATTAATTTTTCACTTTTTTTTACTAATTTATGCCTCCCTAGATAATCGCCAACATGCATTAATTTTAAATGTCCTGAAAATTTCATCCATCCAACATACTAATTAGAGGCAATATGTTCTGATAGAAATATAGAAATGTTGAAAATGCCTGCCTAATTAAGCTTGTGGGAGAAAGATTCGTGTGCGGGTATATACAGTAAACAACTTATAGATCTAAGACGCATATGAGCAGCATATGTGAAAGAATTAAAACAAGAGAAGTAGAGATTAGTGGAGGACATCATTCAATTCTTCCATAATACATAGTATTATCTATGATCTTGAACATTTGTCAATGCCCTGGATTGAACAAGTGTAGTGTTATTGTTGTTATATATATTGTGTTATTGATGTTAATTAGTGTTGTGTGACTGTGAGGAAGCAGATGTGGCAAAGATGGGGGAGAGAGAGTGGTATCTGTTCAGCTTGAGGGACAGAAAATACCCAACTGGGCTAAGAACAAACAGAGCAACCGGAGCTGGGTACTGGAAAGCCACTGGGAAGGACAAGGAAGTGTACAGTGCATCCAGTGGAACCCTACTTGGAATGAAGAAGACCCTTGTTTTCTACAAAGGCAGGGCCCCTCGTGGTGAAAAGACCAAATGGGTCATGCACGAGTACCGTTTGGTTTTGGATGCTCACTTTTCCCTTCCCCACACTCACCCCTCTAAGGTACTACAGAATATTTGCCACGTGCGCCGTGCCAATCAAGTTATCTCCCATTCCTCTTTAATTTTGTCTGCTTCTGGGTTGACCCACCTTCATGTCATTTCCTTTTCACAGCTTCCTTCACAAAATTTATTAATCTTTTTGGATGTCATTTTTACTAGCAATTAACATGTGAAATTGTTAATGATCCTTATCCTTTTAAATTGTAATTCAATGCGTCTTAATTATATGTACATCTCATCCCTTGTGATTAATTAATCTCTAGTGCAAAATAGTAAGATCTCTAACTTACATATTATTAATTACAGTTCCAATGTTTTTCACAGGAGGAGTGGGTTATATGCAGGATATTTCATAAATCTGGGGAAAAGAGAAGTCCAGTGCTCCAAGTCCATGGACATTCAGATGCTTCTTCGTCCCCAAGAGAAAGTGCTTTACCTCCATTACTTGCAAGCCCAAGTTGCTTTACATTTGATCCAGAATCTCAATCCCAAAGTTCATCTCATTCTCAGCGAGACTTCCAAAGCCCGGTTCTGATTCACCACCAAGACCAAAATGGCCACTCTCATAACCCTCGTCTTTTCCCATTAGAAATCACCAACGCCAGAAACCACCCATCATCATTTTCGGACCTATTCTTCAAGCCCCTTCAGCAGAATTGCACCCTGAAGACCAACGAACAAACTATTCTTCCAAAAGTAACCAAAACGGAGGACGCTACATTCTATGATCAATATCATCAATTACTAGATGACCATAATAACATGCGCTGGGTGAACAAGTTGAATCAAAATCCAAGCAATTTACTCAACACTTTCCCTTTTGAGGTGGATGCTGGCTTGATGGCATTCTCAGGAGCTGCAAATGCTCAAGTTAAGGACATATCCACTTCAACACCCTTTAATAGGGTAGGCTTGCAGCAGACGCTAGATTCTTGGCCTCTGGCCCAGCATGTTTGAAATATGTGTGTGTGTATATATGTAATAATTACTGTTCTATAAACAGACCCCTATTAATGTGTTTGAACTCTACATAGCATATGATTAATTAAATAACTCGCGCGTCTATGCATATGTCAAGCTAG

>*Gr*CUC3 |Gorai.002G113300|*Gossypium raimondi*

TCTCTCTATTACATTTACATATTCCAATCGCTCTGTTTTCTTTTTTGGTAGCTCTTCTCTAGCCTTTCAAGGCCTCGGTTCAACCCTCCCCAAATACCTTTTTATCTTGTTCAAATATTTGCATGTTCCTCCATTCATTCCAGTCACAACAACTAGCCTTTACTCTCAACAAGATGTTAGCAGTGGAGGAAGTTTTAAGTGAACTTGGCGGGGAAGAAGTGAACGAGCAAGGGTTGCCGCCGGGGTTTAGGTTTCACCCCACTGATGAAGAACTCATAACATTTTATTTGGCTTCAAAGGTATTCAATGGAAGCTTCTGTGGAGTGGACATTGCTGAGGTTGACCTTAACCGATGTGAACCTTGGGAGCTTCCAGGTATATAATAAATTAACACTCAATACTTATTAGTGTGTCATTGTTAACATGTTACATGTGTATGGTGGGTCTAACTATTTAGTGTACTGTTGATGATGGTAAATGGATAAGATGTGGCGAAAATGGGGGCAAGGGAGTGGTACTTCTTCAGCCTGAGGGACAGGAAATACCCGACGGGAGTGAGAACAAATAGAGCTACCGGAGCTGGGTACTGGAAAGCCACCGGAAAAGATAGGGAAGTGTACAGTGCCTCCACTGGAGCTTTACTTGGCATGAAAAAAACCCTTGTTTTCTACAATGGCAGAGCACCCCGTGGAGTGAAGACCATGTGGGTCATGCATGAGTACCGTTTAGACGGTGACTTCTCCTGCCGCCACACGTGCAAGGTTGGTACATGTGCACCGGTGGGAATCCCACCAGACACCCCCTCCCCTCTTTTTCATTTTTTCCTTTTTGTCGTTTTCTGGGTTGACATATTCTGCTCAGCTGAATGCTAATAATAACAATGATATGATGAAACTGTAGGGTAATTTCTGATGTGATTGCCTTCATTTCACTTACTAGTTACATGTTTGTTGTTCATGCACAGTTCAAATAAGATTTGGAAAAGGCTAAAAGCATGAGTATGTAACACTGAATTGTCAAATACTACTCATGGTATAATATTGAAATGGTGTTATTTTTTGCAGGAAGAATGGGTGATTTGCAGAATAATACACAAAACAGGTGAGAAGAAAAATGGGGTAGCTGCTGCGCAAGGGCTAGGCTATATCTTGGAACTTTCTTCATTATCTTCAACAACAAAAACAACAAATTGCCTCCGTCCACTGCTTGAAACCCCAACTCCTTTGTTAGAATCTCAAACCCAAATTTCAATGCAGGCTGCCCATAACTCTTTTCTGGAAAATGACCTGAAAAGCTTAATAAACCCGGTTGTGTATCCAGCTAATGGGTTCCAACCCTCCTTTACAGCCACTCCCACCACCTTTAGTAGCACACCCGACAAGAACGCCAGCAGCAACTCATCAGCAGCCGCATCGATGCTCTTCAAGTCCCTCCTCTCACATCAAGAATGCGTTTTGAGGGAACAAGCAGCTGCTACTATTCCCAAACAGTGCAAGACAGAAGCTAACTTTTCCAATTTCCAACTGCCTGATTCCACCTTGAGTTGGACGGAGAAGATGCATCCCAACCCTTGTCAAGATCCCATGTTTTTCGACATGGATTATAATAATAGTGTGTTGGGGTTCGCTGAACTCTGAAGGTCAGGTGTTGCGACTGAGGCCTGGGGTCACTTCAATTGCTTTCTAGATTAAGATTGTTAGTTAAGATATCATATTGTTAGCGTTAGGACCCTATCCCTATGTCCATGACTTTCTTTTTATCTTAAGTGTTTGTGCTGGCTTGTTGGTGTAAAATGTAAATGAGTATCCTACAAAAACTCTGAACCCAATCCATGCACGTAACTTTGTAGGATTGGAGAAATTCCTGCACCTAATTCAAGTGATTGTGTATTATTATTATAATATTTATGGGCCTTATGTATATATTTATTTGTAATTACAAAA

>*Cs*CUC3 |orange1.1g016283m|*Citrus sinensis*

ATGGAAGACGTTTTGAGTGAACTGAATGGAGATGAAGTGAATGAGCAAGGGTTGCCACCAGGGTTTAGATTTCACCCAACTGATGAAGAGCTCATCACATTTTATTTAGCCTCAAAGGTCTTTAATGGCACCTTCTGTGGTGTTGAAATTGCTGAAGTTGACCTCAACAGATGTGAACCTTGGGAGCTTCCTGGTAAATACTTAATGTCTCTCTCTTACTCACAAGTCGTCCGTTATATATGTCCGGGTGAAACACACACATACCCCTTTCTGTAACCTGTTAATCCTGACAAAGCTGCGAAGCTAGTTAATTAATAATGAATTAATGTGTTAATAACATTTGCACGTTTTAATATTTGTGTAGATGTAGCAAAAATGGGGGAGAAAGAGTGGTACTTCTTCAGCTTAAGAGACAGAAAGTATCCTACTGGATTAAGGACAAACAGAGCAACAGGAGCTGGTTACTGGAAAGCTACAGGCAAAGATAGAGAAGTGTGCAGTGGCTCAAGTGGAGCCTTACTTGGCATGaAAAAAACCCTTGTTTTTTACAAAGGCAGAGCTCCTCGTGGCGAAAAGACCAAATGGGTCATGCATGAATACCGCCTCGACGGTGACTTCTCAtACCGCCACACGTGTAAGGTACTTACTTACTATTCACTAGGACCCTCTTACATAAGTCCTAAACTCTCAACTCTTCCCAACACGTCAGACGTGTGCCGTGCCAATCAAGCTAGCCAGCTAGCTTAGTAGCTTTTGGCTAGCTATCTTTTGTCACTTTCTGGGTTGACTTATCATGTATGGTATTTTGTATATTTAATTACTGGTTGTAGCGTTACAACTTACGCGTGTGAAATGCGTGGGGACTGTAGGAGGAATGGGTGATATGCAGGATATTTAACAAAGCAGTAGTGAGTGGGGAGAAGAAAAATGGATTGCTTCTCCAAGGACAACACTATTTGTTTGAGGCAGCTGCAACAGCTGGTGCTTGCTTGCCTGCTTTGCTTGATGCCCCAGGGCCAGCAACGACAACATTACTGTTGGAATGTCAGTCTCAAAACCACAACCCAATCTTGGAAAATCTTCCGAACCATTTCGTGAATCAGCAACAAGACAACCATCATCATCACCTATTCCCAGTAAATGGCTTGTTTGAGACCTCCGCAGTAACAAACAAACACATTTTGATTAACAACATCACTGAAAACATTGGCAACAACACATCACCATCCATGCTTTTCAAGGCACTCCTCTCACATCAAGATTTCAGCTGCTGCAATGAATTAGCCCCTAGTCCCAAACACTGCAAGACAGAAGCAAACTTTTCCCATATCCAGCTGCCTCCTGCTACTGCTGCTGATGACAATAGTAATGATAACTGGAGCAATTGCTACTGGATGGACAGCAAAATTCAACCAAACCCATATTCAAATCCCTTGTTTTCTGAGTTCGATTGTAGCTTTCCTGGACTCACACAACCCTCTGCCTTTGCCGCTACTGCTGTCAATGACATGTCCACTTCAATTGCTTTCAACAGAACCGGCTTTCAAGTCGTCGAAAAATCTTGGCCATTGGGTGCTTAA

>*Pv*CUC3 |Phvul.009G008000|*Phaseolus vulgaris*

TACTCCTCCCCTCGTTTTCAGAACTCCCTTTATAACATTCATTATATATCTCACTCCAATCCCTTTACCTCATCAATATTTTCCTGTTCTTAAAGCTAGCTATCGGTTCAGTCTTTCACTTTTCTTTGCATAAAAAAGAAGAGGTTTGAGTGGATATTTTAGGAGGACATGCTGGCAATGGAAGACCTACTGTGTGAACTTAGTGATCATGAAAAGAGAAACGAGCAAGGTCTGCCACCGGGTTTCAGGTTTCACCCCACTGATGAAGAACTTATAACCTTTTACTTGGCTTCAAAAGTCTTCAATGGGAGCTTCACTAGTGTCAAGTTTGCTGAGGTTGATCTCAATAGATGTGAGCCGTGGGAACTTCCAGGTAACATCAATATATATATAGATGTATTTGTATTCTTCTGCCCAAACCCTCACAAGAATGGTGTGCACCTTGAGTTTCATCAAACAAATTATAAGAGAAAAAATGTTGACAGTAGATAATATTTTATGTTTGTTCAGAGTCAGATGAAGTAATAGAGTTTTAGAAATTTATCTTATTATTAGTTACAGTTCCTGCTGTTTTCGGCCTCATATTTAGGTTAATTCGTGAAAACTAAGACATATATTGTGGGCTATCCACTAGCATGTCACCTCAAATTTTCAATGGCGTCATCAACTTCCCTCCTTTGGATCTTCTTGTTTTTCCTGTTCCTAATTTTTGTGTGCTGAACATCGAGAGTGGACAGCTAGCTATATAACTCCAACAAAGTTTGAACAAAATTTAAGCAACTTTAATTGGTCCCAAGAGTTTTCCATCCAACATATACTAAGTAGAGGCAAAAGAAAATAAACCTATTTCATCACCTATATATGCCCTGATAGAAATGTGAACTTTTTAATGTGCCAGACAAATCAGCTACCTGGGAGAAAGATTCATATGAACCCTATTAACCAACTTGGTTCATGTACATTAATTTACAGATTCAAGTCAAATATGAGATGATGTTTTATAGGTTTCTATAAATTAGTTATACATGTTTTGCTTCCATGCCCTGGTGTACATGCATTGAATATTGATGTCAGAAATGTTGGGTGTGGAAGCAGATGTGGCAAAGATGGGGGAGAGAGAGTGGTATCTGTTCAGCTTGAGAGACAGAAAATATCCAACTGGACTTAGAACAAACAGGGCCACCGGAGCTGGGTACTGGAAAGCCACTGGGAAGGACAAGGAAGTGTTCAGTGCATCAAGTGGAACCCTACTTGGGATGAAGAAGACCCTTGTTTTCTACAAAGGAAGGGCCCCTCGTGGTGAAAAGACCAAATGGGTCATGCATGAGTACCGTTTGGACGGTGACTTTTCCCTTCCCCACCCTCACCCTCATCACATTTCTAAGGTACGGAACACAACCATCAAAATTAGACAGGATTCTGAATTATTATTGTATTTTTCTTCACACTCCTTTCTTTGTATAATAAATTCTCATTAATAGATATTTAAAATTTCAATTTTGTCTGAAGAATGGTGGTGTAAACAATATTATACAATGCAGAATCTACAGCACGTGCGACGTGCGAATCAAGTTATCTCCCTCCCTCTCTCTGCTCCTATTCCTACTCCCTCCTAAACTTTTTGTCTTCTTCTGAGTTGACCCACCTATGTGCACAGTATGGCACCATGTCTTTTCCTTTTCACCAACCAAACATGACCCTTTTCACTTTCCTTCATAATTTATTTATTACTAACCTTCTGGGTGGTTCTTTTGATGTGATCGATTGAATTGATTTTTATCATTACCAACCTGTGAAATTGTGAATCCCCATTAGTTGTTAATCAAAACTTACATTGCATGTTGATAATAGTTCCAATGTTTGTTTTTCACAGGAGGAGTGGGTGATATGCAGGATATTTCATAAATCCGGGGAAAAGAGGACTCCACTGGTCCAAGTCCAAGGACATTCAGATGCTTCTTCGTCTCCAACAAAAAATTCTTTACCTCCTTTACTTGCAAGCCCAACTTGCTTTACATTGGAACTAGAATGTCAATCCCAACAAAGCCCGGTTCTCATTCACCACCACCAAGACCAAAACCACCTCTCTCACTCTCACCCTTATCTTTTCCCATTGCATGCATCACCACAACTCACCAACGCCAGAAACCATCCTTCATTTTCTGACCTGTTCTTTAAGCCCCTGCACAACTCACAACAAAACTGCATCTTCAAGGCCAAGGAAAAAACAGCTCCAAAAGTAGTCAAAACGGAGGAGGCTACAGCATTCTATCAGTACCATTTACTAGGTGACGCCAATAACTTGCGGGTGAACCAAAATCCGAGCAATTTCCCAAACCCTTTCCCTGATGTTGAGGTGGATGCTGGGCTGATGGCATTCTCAGGAGGTCCAAATGCTGAAGTTAGGGACATGTCCACCTCAACTGCCTTTAATAGGGTAGGGTTGCAGCAGGTGATAGATGCTGCTCATATCGGAATAGATTCTTGGCCTCTGCCCCAGCATGTTTAAAATGTGTCTGTTATATATATATTCATATATATTTACTGCTCTACATACACAGACTCCTTGTCTCCTACCTTATGTATTTGCATAACATATAATTAATTAAATGACTGCTTCTATTGTATGTATTCCTGCCTATGTCAAGCTAGATCTTCTACTAGATTTGTTACGTTCCGTGACTACAACCTCAG

>*Sl*CUC3 |Solyc12g036480|*Solanum lycopersicum*

ATGGATGAAAATCTTCCTCCAGGGTTCAGGTTTCATCCAACAGATGAAGAACTCATTACTTGTTATTTAAATAACAAAATTTCTGATTTCAATTTCACTACTAGAGCTATTGCTGATGTTGATCTCAATAAGTCTGAGCCTTGGGACCTCCCTGGTAATTATTTATGTATTTAATTTATATTTCGATCATTCAAGTCATCCTTGTTTGCAACAGATAATAAATTTTGTAGTTAAACATCAAAATTAGTCAGTAGTCTATTTTAGGGATGAATATATTAGTAATAGATTATTAAAAAAAATTGTTAGTTAATTATGAACAATTTATACAAATTGTTGTGTTGTTCTACCTCAATCGATATATGTTTATATAGTTTTTACCTATTTAATATTTTTTGAAAGAAGAATAGATAGGTATAAATTTACTATGTGCATTGATGTATAATTTAGCATGTAGTTTGGGAACTTCAAATTGTATATTTCACTCTTGGGAGAACAAAAAATGAGATATTTAATTGGCATAATATATAAATTGATTTCAAATAATATTTATGTCCTTTGACTTTGGGTATACACAAGTAAATACTTAAATTTGTATAAAATTGAACAAATAGACATGCATGTCCTACGTGACATCATACATGTCGTTTTTTATCGTACGGGGTGTCATACTGTGACATGTAATACTCATGTGTCTATTTGTTCAACTTTATACAAGTTTAAGTGTCTACTTGTGCATACACAAAATTGAAGAACATAAACATAAAATGAGGTCAAGCCAAAGTCACATTTATGTATTGTGCCTTTTAATTTCTTGCTTCTTCCGTTATCTTTATTTTTCATGAACAAATATATATTTTATACAATTTTTTATATAGTATAATTTTTTAATGAAGGGTGTTCAACTGACCACCGCATGAGCATTATAACTCCATGTAAATGATGCTTAATTATGTAGCAAACAAACAGAAATTGACATTCTTATTTTGCAAAAGCCCTACGCATTTATCAGTTTTTTAGTTACTACTTCTCTATGTTGACAAAACATTGCGTGTGTAATTGGACATGAAGGAGGAAAAAATGAACTGTTTATGTGAATAAGCACCAAATAGCAACCACTATCATAATAATTAAAAAAAAATGAAATATTTCGATTGTTGTTGTTAATTTCTTAATGTTGTACTGGTACAGCAAAAGCGTCAATGGGAGAAAAAGAATGGTATTTCTTCAGTCTAAAAGATCGAAAGTACCCAACAGGGCTTCGAACAAATAGAGCTACAGAAGCAGGCTACTGGAAAACAACAGGGAAAGATAAAGAGATATATCGTGGTGGAACGGGAGTTCTTGTTGGGATGAAGAAAACCCTAGTTTTCTATAGAGGAAGAGCTCCTAAGGGTGAAAAAACCAATTGGGTTATGCATGAATATAGAATTGAAACAACATTTGGTTACAAACCTTCTAAGGTATGTATATTCCACAATCAAAAAACAATACATTTGACGCGAAATTTAAAAAAGAAAGACTTTTAAATTTGTTGTGTAAAATGAATCATACATATTTTGTTTGGCTAGAAATTATTTCGTTAAGGGTAAACTAGGTATTTTAAAGTTAAATTATTACTAAATATAGAAATATAATTAATTTTGGAGCCAAAAAGAAAAATAAATCATATAAAATGAGACAAAAAGAAATAGGATTCGCCATGAGATAAGGAGTAATCATATATATTATATCTTTAAATTTATCACTTATTCTAACACTAACAAAAAAAACCCTCAATTCTAGCTTATACAACTCGCTTTTCTTTTTATTCAGTTTTTTTTTAAAAAAAAATTAATTGCTCCATTTCGAAAAGAATGATTTAGTTTGACTTGGAACAATATTTAAGATTTTTTTTAATATGGTTGTTTTAAATTAAAGTTATGTCAAATGTACCAAAATACCCTTTAATTTTGTAGCCTTAAATATGCCACGTAGAAAGTTGAAGGTAAAATTTACCAAAAAAAGAAAAAGAATCATTCTTTTTTAAACACATTAAAAAGAAAACTAAGTCATTTTTTTAAATGGAAGGACTAACACATTTCTATACTATGTAAGTTGTAACAATTAAATTTTAAAACGCCTATTTTACTCTTAATGAAATGATTAGAGCCACAAACATCTATCCTTTATTTGAGGTACCACATGTTTCAAAAATCTTTCTTTCTTAAACTCAATCTCAAGTCAAACTAATTTATATAAAATGAAACACAAAGAAGAGAGTATTATTATTCCGCGAATAATATATTATATTTTTGTGTTCTTACTTTCAGGAGGAGTGGGTAGTGTGCAGGGTGTTCCAAAAGAGTTCAACTGTGAAAAAGCCACAACCAACATCATCTTCTCCATTATCCCTAGAGTCACCTTGTGACACTAATTACACAATAACAAATGAGCTTGGTGATATTGAGCTACCATTTAATTTCAACTACCTTACCACTACTCCATCAACCGCGATCAATAATATTTCCTTGCATAATTACAACAACGATAACATAAACTTGGCTGCTGCAACAAGAGAAGCAAACAGTCATCCATTACTACCTTGGTCTTCCAACTTGTTAAGCTCAAATCTTTCATCAGTAAATTCATTACTTTTTAGGGCATTGCAATTAAAGAGTTATTCGCCAAGAGAACAAGCTACAACGACTCATGACTACGCGTTTATGCTCCCACAGGAGAATATTATTACAACGCAATTTGGAAATGATTTTGCTGTGAATAATATTGGGGCACCGTCTTCATCTACGGTGTTAGATAATTCTGTACAGCAGCAACAACAACAGCAACAAGAACAATCGTACAAATTGGACTCCAATATTTGGTGA

>*Ss*CUC3 |Sspon.06G0001780-1A|*Saccharum spontaneum*

ACCACCACCAGGCCATGAGCGACGCGCTGTGGGACCTGCTCGGGGAGGAGATGGCGGCGGCGGGCGGCGAGCACGGCCTTCCCCCGGGGTTCCGGTTCCACCCCACCGACGAGGAGCTGGTCACCTTCTACCTGGCCGCCAAGGTGTTCAACGGCGCCTGCTGCGGCATCGACATCGCCGAGGTGGACCTCAACCGGTGCGAGCCGTGGGAGCTCCCCGACGCGGCGCGCATGGGGGAGCGCGAGTGGTACTTCTTCAGCCTCCGCGACCGCAAGTACCCCACGGGCCTCCGCACCAACCGCGCCACCGGCGCCGGCTACTGGAAGGCCACCGGCAAGGACCGCGAGGTGCTCAACGCCGCCACCGGCGCGCTCCTCGGCATGAAGAAGACGCTCGTCTTCTACAAGGGCCGTGCGCCGCGCGGCGAGAAGACCAAGTGGGTCCTCCACGAGTACCGCCTCGACGGCGACTTCGCCGCCGCTCGCCGCCCCTGCAAGGTACTAGTATGTGTACTCCTACACGAAACGACGCAACTGACTAGGTAGCTAGATTTACTACATGCATGAATGGATTCCTTAAGGTTCCAGGGTAGTAGATTCTTCTTTGAATGCATGCCGTAAAAACGCAACAGTGTGACAGTAGTAGAGATGTGATATCATTGACAAGAGGTAGGAGTTGCAGATATGCGGCTGCAATGAATGTGGCTGCACGATATGCATGCAGATTGATACTAGATGTGTGCTTTATGTAGGAGTAATCCTTCTGTGCATTCATCAATGGCACTACACTAGCTGGTGTAAGCATGATAATGCACTGGTACAACAACCACAGTGCTTGTGACCAGCTTCAGCGCATGCATGCATGGCCAGGAGTAGTTCCATCTATCTGTGCTCGCAATCTATTTGCACTGACACACAGGATCCATGCCAGCCAGCTACTGAATCCTTCTGTGTTTTGTTTTTGTGTTGTTGTACTGCTATCAGGGTGCATCATGATGAGTACTTGGCATATCTCCAAACACTGACATGTACCTCCCTTCCAAATCTCTTCCGTACAATACAATGAATAGAATGTCCCCCAAGATAAGCTGTGTTTAGTCCACCAGATCGAATGTTTAGACACATGTGCTAATGATAGCTTAATTAGGTTTAATAAATTCATCTCGCGGATTTTAGACGAGTTATGTAATTAGTTTTTTAATTAGTATGCGAAAATCCCTTCCGACATCAATAAAACATCTGATGTGACACCAAAAATGTTTTATTCACCAACTAAACAAGGCCAAACTTGTAACAAGAGCATCGGATGTGCTGACTGTGGGTTCCATTTTTTAATTTGTAAAACAAATGTGCCATCTGCAGAAGTTGTTCATTCAGGAGGACTATGCACTAAAATTTCAGAAAAATATATATACAATATACACTGTTGTACTACCTCTGTGTGCTGCATGCCTAGAGATCACATTCTCGGATACGATTGAATATTGACGAATCTCTATGTCTATACTCCTACTCCTGACTGATGATTTGCTGAATCTTTTCATACGCACATGCAGGAGGAATGGGTGATCTGCAGGATACTGCACAAAGCAGGCGACCAGTACAGCAAGCTGATGATGGTGAAGAGCCCCTACTACCTCCCCATGGCAATGGACCCCTCCAGCTTCTGCTTCCAGCAGGACCCCACCGCGCCTCCCCTCCAAAACCCTAGCGGCTGCATCCCCTTCCAGCACGGCCACCCCAGCATGCAGCCGCCTCCACTGCCGCCGAGCAACCATGGCAAGGTCGTCTTCACCGGAGCAGCGGCGCCCTGCATGCAGCAAGAGCCGGCAAACGGCAGCAACAGCGCCGTGCTGCCGATGCCGCCGTTACCTCACTTCACCCCCATCGTCGCCGGCAAGCCGGCCCCGGCGCCGCCGCCCCAGGTCGGGGTCAACGCCGGTCCACAGGAGCCACCGCCACCGCCACCTACCTGGCTGGAGGCCTACCTGCAGCACGGTGGTGGGTTCCTTTATGAGATGGGCCCAGCTGCAGCGCCCAGGGGCGCATGATCCG

>*Ac*CUC3 |Aco000744|*Ananas comosus*

ATGCATACCAATATGGGGGAGTTGGTGTGGGAGTTGTTTGGAGAGGAAACTTACAATGAGCAGGGATTACCTCCTGGATTTAGGTTCCATCCGACTGATGAGGAGCTTGTGACCTTCTACTTGGCATCCAAGGTGTTCAATGGGGGCTTATGTGGGGTGGATATAGTCGAGGTTGACCTTAATAGATGTGAGCCATGGGAGCTCCCAGGTTTGTATACGTTTTACACCCATGGTTTAATTAAGATACGAAACACTTTTTCATCTTAACATTCATTTCCGTAAAGATGGGCCAAATCTTTCTTTTGGCCTGAAGAAATTATATTTCTATATTTTGTGGCCTTTCACGTACGAACGTAAACAAAAGCTGCATTGATGCCTCTCCACACTGGAAGGGTGGTGATCGTAGTGAGAGTCAGTGTGTTTGTTGCCTTGCATTAATTGACTAACTAATAAACTAGTGCCCCTTTGTTACATGTATAGTCATGATGCATGAGTTAAAAATTGCGGCAATCTCTATATCATCAAGTTTCACATTTCTCAGCTCTTCTTCTTTCTCCTTATCTTCTTGTTTAGACGCGGCAAAGATGGGAGAGAGGGAGTGGTACTTCTACAGTCTCCGCGACCGGAAGTATCCGACGGGGCTGAGAACGAACAGGGCGACGGGGGCCGGCTATTGGAAGGCCACCGGAAAGGACAGGGAGGTACACGGCGCCGCCAATGGGGCCCTTGTCGGCATGAAGAAGACGCTCGTCTTCTACAAGGGGAGGGCGCCGCGCGGCGAGAAGACCAAGTGGGTCCTTCACGAGTACCGTCTCGAAGGTGACTACGCCTGCCGCCACCGCTGCAAGGTGCGTAATCTTTCACTCGATTGCATGCTGCATGTTCTGAAACTTTAAGGTTTTATTTTGAACATTTTGGCAATTTTTGCCCAAGCTAAGATTTTGCGTACTCCGCGTAAATTTTCTTAGTTTACAGTTGCAGCCCATAAAAGTTGCACATTTTTCCAACTTAGCATGAAATAGCATTATTAATTGCCAACGGGCATAAATGTGTTTGATTACTCTTATTTTCAAGTTTATTAATTGCAGTTGCATCATTTTATATGTTCCATATCATCGGCCTTCATTTGCCTGTAATAATGTTGGTATGTTTGTACGTTGCATAGTTGGTTCCATATATATTTGTAATGCGTCATTCGGACCGCATTTATGTTACGTAATATCCATATATATGGTCTCATATTGCGTAACATATATTCTTCTGGATGTTACGCAATATGAGACCATATGCATATCATCCATGTATAGCTCGAAGATATAGCTATATATATGTATTCCATGAAATTTTCATCGTGCTACATACTGTTACGTGATTAGACTCTTTGCAAATTTAAAATAAACTAAGAACCGTAGAATAATATATCCTCTCTTTTGTATTTAAGTTAAGATGGTCCCTAATGCTTGAATAAGCACCAATTGATCGAGTAGGCAGAACTCTCTTCGTGTGTATATGCGACGGGGAATATATATGCGCCATTTGTTCTATTGTTTTCAACATCTCATGGCCATACTGATCTAATTGTTTACAAATAAATGTTTAATATATAAGCCATCATGTTGCATCAATTGATGTGCCTTATATATAACCTAACCATGTTTGGATCTAACTTCCTTCTTTGGCACGAAAAGTCAACTCAACCCCGCCTGCATTGAGTTGACATTTCTCTTCTTTAAAACAATATATATATATAGGTCATGTCACTCAGTCGTATTCTGAATTATGTCAATCCCTACAGAAAACATTGTTCGAAAAAAAATATTGATACGTAATGAATTTCTCTTTTGCTCTGCTATTGTTCATCCAATGCTACCAATTTATAGTAAAATGTATACTAACAACATCTCCTTGGTGTGGGACCTTTTTTTTTTTTTTTTTTTTTTTTAATATTGCAGGAAGAATGGGTGATATGCAGGATATTTCACAAGACAGGAGGAGACAAGAAGAACCAATACTACCCAAACCCCTCATATACAATAAACCCATCCTCTTCCACTCCCAGTACTTGTATCCTCCCATTCCTAGACCCCCAAACCCTAGAAACCCCTCTCCAAACCCTCCACAACCACCACCAACCCTATTTCCATCTAAACCAAGAACCCATTAATCCCCTCTTCCCCCTCCCTCCTCTCCCTTCCTTTGCTTGTTCCTCCACCTTCCTCCCATCCTTCCCTAAAAGCCCTCCAAAGGAAGAGGACACAAATGCACTATTAAACCCTAATGAGGAGGCCATGTTCCCTGCTAATTGGCTCGAAACGTACATACAAAACCCCTTCGTTTATGAGATGGGCTTTTCACTTCCGGGCCCTGGGGCACCAGTTTATGACGTGCCCCTCCTGGGCTACACTGCCACAGGAGAATCTGGACCGTTG

>*Os*CUC3 |LOC_Os08g40030|*Oryza sativa*

ATGGGGGACGCGCTGTGGGAGATGCTGGGGGAGGAGATGGCGGCGGCGGCGGCGGCGGCCGGCGAGCACGGGCTGCCGCCGGGGTTCAGGTTCCACCCCACCGACGAGGAGCTCGTCACCTTCTACCTCGCCGCCAAGGTGTTCAACGGCGCGTGCTGCGGCGGCGTGGACATCGCGGAGGTGGACCTGAACCGGTGCGAGCCGTGGGAGCTGCCGGAGGCGGCGAGGATGGGGGAGAAGGAGTGGTACTTCTTCAGCCTCCGCGACCGCAAGTACCCGACGGGGCTGCGCACCAACCGCGCCACCGGCGCCGGCTACTGGAAGGCCACCGGCAAGGACAGGGAGGTGGTCGCCGCCGCCGCCGCCGGCGGCGCGCTCATCGGCATGAAGAAGACGCTCGTCTTCTACAAGGGCCGCGCCCCGCGCGGCGAGAAGACCAAGTGGGTCCTCCACGAGTACCGCCTCGACGGCGACTTCGCCGCCGCTCGCCGCTCCACCAAGGTAACACCCACCTGCATTGCCATTGTCATTGCCATTGCATCTTTGTCTTTCTGAAAAAAAAAATCAGTTTCATGTCACTGTTCTGAACTTCTGAAGAAGATAGCTGCTGCAACTAGTTCTTCAGAGTTCAGAGTAGATATTGGTGATTTGGTGCATTCAATGTAGCATGCATGCATGCGTGCAAGAACACATAAGATGACATATGCATGCACAGATTGATGCTGTGGTATGCTACTACTATGCTCTATGCACTTTTGTGCATTCAATGGTGTAAAATACTGTAATGTGTGCTGCTTGCTATACATTGGTACATCCTGCAGTGGTGGTAGTACAGTCTCAGCAGTGTGTGTGGAGTACTAGACTACTAGTAGGAGTAGTAGTTTATTTCTGCTCAAAATCTCCTTTGCACTGACACAAGATCCAAGCTAGTGTGATTCCCTCCCTCCTGTGTTTTGTTCCCTTCAGGGTGGCTGTACTTTACATATCTCCAACCATTGACATACCTCGTCTCCAATCTTTCTTGTGCAGATCGTAAAAAATGTGTCAAATAGATAAACTATTCGTTGCAGCATTGCATCAATATACAGCTAGTAATCCATGCCTCATGCTGATGCATGCTATGCACATCTATAGGGCCAAAAGAAAAGGAAGAAGAAACAAAGAAAGAAATCTCTGCAGAAGTTCAGAGCTAAATATATAAATATATATCTTAATATACTCTATGCTATATTTCTGTCCAACAAGATCACAATCCGTAGAAATGAATACTATTAATTTGATCTTATGAACACTTCATCATAAACTGAACTGACATGTAGACATTGGTTGTTTTTGATACTACTATCATGCAGGAGGAATGGGTGATCTGCAGGATCTTTCACAAGGTAGGAGATCAGTACAGCAAGCTGATGATGATGAAGAGCCCAGCCAGCTACTACCTCCCAGTGAGCCACCACCACCCCAGCAGCATCTTCCATGACCTTCCTCCGGTCCCATTCCCAAACCCTAGCCTCGTCCCCTTCCACCATGATCTCCCCACAAGCTTCCATCCTCCATTGCTGCAGCACAGCCATGCGAACAGCAAGAACAGCAGCAGCAACAATGGCGGCTTCGTCTTCCCCAATGAGCCAAACACCACAAACAGCAGCGATAACCACATTTCTTGCAATGGCGCCATGGCTGCTGCTGCTGCTGCTGCTTTTCCTTCCTTCAGCTGTGCTAGTACTGTCACTGGCAAGGGGGGCCCACCGGCGCAGCTCGGAGTCAACGCCGGTCAACAGGAGCCACCGCCACCTACCTGGATGGACGCTTACCTGCAGCACAGTGGATTCATTTATGAGATGGGCCCACCTGCAGTGCCCAGGGGCGCATGATCTGGGCCGTTGGTTTCTTTAGGGCTACGATCTGGATATCCTTTAGTTTGCAAGTGTGGTATTTAGACAAGCACTTGTTTAGCTGCTTTGAAATTACTAGTGGTTGTTAATTAATTAGTCATCATTTGGTTATTTTCATGCACTCAGGTGTGTACCTAGCTTGCATGCATGAGCTGCAAGCATTGGTGCACTGGGTTGAGTATTACTACTAGTAACTACTTGCTTAATTACTACTAGTATTACTTTGTCTGTGAATCCATGCGTGCATGTATTCACATGCAATGTAATGGCTACATATATAATCAGCTAGTCTGTTTTGTTAGATGTTATGATAAGTGAACAATGGATTATATTCA

>*Bd*CUC3 |Bradi3g40085|*Brachypodium distachyon*

CCCCCCCCACATTCACCACACAACACCACCATAGCCAAGAACTCCCCAATAGCTAGCTCTCACTCTCAGTGCACCTCAGCTTGCTTCTTCTTCCAAGCTTGTACATCTAGAGTAGTGAGAGTTTCACAAGATCGCCGTCGTCATGCACCAGCACCAGCACCCTGCGGCGGCGGCCATGGGCGCGGAAGCCCTCTGGGACATGCTCAGCGAAGACATGGCAGCCGCAGCGGCCGCCGCAGCCGAGCACGGCCTCCCCCCGGGCTTCCGCTTCCACCCCACCGACGAGGAGCTCATCACCTTCTACCTCGCCCCCAAAGCCTTCAACAGCAGCAACGACAACGACAGCAACTTCTCGGCCGTGGACTTCATCGCGGAGGTGGACCTGAACCGGTGCGAGCCGTGGGCGCTGCCGGAGTCGGCCAGGATGGGCGGGGAGCGGGAGTGGTACTTCTTCAGCCTCCGCGACCGCAAGTACCCCACGGGGCTCCGGACCAACAGGGCCACGGGCGCCGGCTACTGGAAGGCCACGGGGAAGGACAGGGAGGTCGTGTGCGCCGCCACGGGGGCGCTCATCGGGATGAAGAAGACGCTCGTCTTCTACGAGGGCCGAGCTCCCAGGGGACACAAGTCCAAGTGGGTGCTCCATGAGTACCGCCTGGACGGCGACTTCGCCGCCGACCGCCGCTCCTGCAAGGTACTAGATCCATATGTGTTCCATCACACTGCATTTCTCCATGGATGGCACGCTTGGCTTCCTTGATCTTCTGAATCTGAAACTGAAACAGTTGCTTTGAGAGTTCAAGCCTAGATCAGGCCTCAGGGGGATGTGTTGTGATGTCTAGCTTGCATGGTCAGTAGAGTTGTAGATGTGGTGCATTCAATGCGGTGTGCATGCATACATAACGGTGACATGAGCATGCAGATGACTAGATGAGCATATGTGCAGATTGATCTGGAATATATGCTTATTATATCTGTGGTACACTTCAGTTCATTGCTGTACAATGCTAGCAGTAGTATCATAAAGAATGACGATAATGTTTTACGCGCACAGTGGTCTCGTATCTGCTCAAGATCTGTTTGCAGATTTGCACAACCGACACAGGATCTGTCTGAGTCTGAGCTTCCTATCTAGGGATGCCCTGCATCCATGTTTGTTCGATCCCTCTCAGAGTCGTCTCAGGGAATTTGCATATGCATGCATGTCGCCAAGCATTAAAACATGGAGTACTACCCAACACATGCATGTTGCATGAATGCTGAGTTGCTGACATGCTCTCAGGAGTAAAATATATATACATATACATGATCCTGTACTGCTAGCATCTCTGTACTTCTATCTATATCTAAATAGAATGATATTTACTACTAGTAGTACATTAATTGATCTACGTTCTTCTGAACAATGTACTACTGATCTACTATATCTTTCTGAACAATGTTCATCCATTTTTTGGTTGGTTGGTGATTTTGCTGAACACATATATTAATCGATTGTACATTATATGTGATTTATGCAGGAGGAATGGGTGGTGTGCAGGATCCTCCACAAGACAGTAGACCAGTACAGCAGCAAGATGATGGAGATGAGGATGATGAGCCCCTACCACCACTGCTACCACCCCATGAGCCACCACCACCACCCAAGCTTCGTCTTCCAGGACGCGCCTCCCGTCCCCTTCCCAAACCCTAGCGGCCAGCTCCCCGTCCCCTTCCTCCACCACCACCATGACCTGATCCCAAACCTTCAGCAGCCCTCGCCATTAACAACGCAGCACCACCACCAGCCCCAGGCGGCCGATAAGAACTCAAGCAGCAACAATGGCGGCTTCCCAGTCCCAGCCGCAGCAGCGGCTTGCATCCAAGATCAGCAGCCAGACAACAACACGGCGCCATACTTCCCTTTCCCTTCCTTGGCCTCCGCCGTCACCGTCGCTGCCAAGGCGGGCCCACTGCCCGGAGTCAACGCCGCCGGTCCGCAGGAGCTGCTGCCGCCGACGTGGCCGCTGGACAACTTCCTGCAGCATGGCATTGCCACCTACCTCTACGAGACGGGCCCACCCGCAGGTGCCCCCAGGGACGCGTGACGTGCATGATCGATCCGGACCGTTGGTTTTCTCCATCGGTCGGTTGCTAGTTTAAATATTGCAAGTGCATGTGCTCGATATTTAGATAAGCACTTGTGGCTTCTTAATTTTTCGATTATTAGTCCTTAATTTAGTCTTCATGTACTGATCATGTGGTCCGACTGTAGTACGTTCGCAGTAGCTAGCTAGTAGCGGTGCCTTTGCAACTGATTTGCTTGTATGCATATGCATTGGATCATTGGAGTAGTACGTACGGTAGTGACTAGTACTCCTTTGATCCATGCATGTATATGTGTTCAGACATGCAGTATGCATTGTTCTATTAATCGATATTTGAA

>*Zm*CUC3 |GRMZM2G430522_P01|*Zea mays*

TCTCCATCTTTTAACCTTTCCGTTCCATACATATCTCTCTCTATTTCTCTGGGACAAAGGAACAAAGAGCGAGCGAGAGAGAGAGAGAGAGCAGCTCTCAAGGTCATGGTCTTATGGAGGACAGGAGGAGCATGGTGGTGCTACCTAGCAAGAGCTCCCCACTATAAAGCGCCCCCACACACCATACCACAGCTCAGAGCTTCTTCTCATCATCTGGTAGAAAGAAAGAGTGAGAGTGAGGTTGGCAAGGGTATAGGGTTCTTGGTCGATCAAATACCTTTCCCCTCTTGGATTCTCATCTTCCTGCTTCGTTCTCACCAGATCGATCTCACCACGTGCCTGCGTAGCAAGCCACTCTGTATGCACCATCACCACCAGGACCAGGCCATGGGCGACGCGCTGTGGGACCTGCTCGGGGAGGAGATGGCAGCGGCGGGCGGGGAGCACGGGCTGCCCCCGGGGTTTCGGTTCCACCCCACCGACGAGGAGCTGGTCACCTTCTACCTCGCCGCCAAGGTGTTCAACGGCGCCTGCTGCGGCATCGACATCGCCGAGGTGGACCTCAACCGGTGCGAGCCGTGGGAGCTCCCCGACGCGGCGCGCATGGGGGAGCGCGAGTGGTACTTCTTCAGCCTCCGCGACCGCAAGTACCCCACGGGCCTCCGTACCAACCGCGCCACCGGCGCCGGATACTGGAAGGCCACCGGGAAGGACCGCGAGGTGCTCAACGCCGCCACCGGCGCGCTCCTCGGCATGAAGAAGACGCTCGTCTTCTACAAGGGCCGGGCGCCGCGCGGCGAGAAGACCAAGTGGGTCCTGCACGAGTACCGCCTCGACGGCGACTTCGCCGCCGCTCGCCGCCCCTGCAAGGTATGAACTATAGTACATGTAGGAGTACGTACGCAGAGCTAGCTAGATTTTCTTGAGTTCCTACAACTAAAACTGAAACGGCGCAGAGCTAGCTAGATTATTACTCCATGAATGGGTTACTTAAGGTTTCAGGGCCGATCTTTTCACGCTTTCTGTTCTGTTCTCAATGATTCTTCTTTTGAATGAATGCCCGTGAAAACGCAACACAGTGTGACAGTAGTAGAGATGTGATGCCATTGACAAGAGGGTAGGAGATGCAGATATGTCATATGAGATACTGCTATACTAGTACTGCTGAGTGCTGGCTGCAATGGATGTGGCATGCATGCATATGCACCATAGCCGCAGTACAAGGAAGATGGCTTGGGTGACATTTGCATGCAGATTGATAGATGTGTGCTTTATGTAGGAGTACTCCTTCTGTGTGTGCATTCATCAATGGCACTACAATAGCTGGTGTAAGCATGATGATGCACTGGTACAACAGCTAGCCACAGTGTTTGTGCCCAGGTTCAGCAGCATGCATGGCCAGGAGTAGTTCCATCGTCTATCTCTGCTCGAAATCTATTTGCACTGACACATAGGATCCATGCCAGCCAGCCAGAGCTACTGATGCTTCTGTGTTTTGTTTTTGTGTTGTGTTGTGCCTTGCCATGCATCAGGGGTGCATCATGGATGTACTTGGCATATCTACAAACACTGACATATACCTCCCTTCCAAATCTCTGCCGTACCATACAATGAATAGGATGTCCCCAAGATAAAAACATGAGCATCCAATGTGCTATCTGCAGAAGTTGTTCATCCATCCAGGAGGGCACTCAATTTTTTTCAGAAAAAAATATATACACTGATGTACTACCACACTATGTTCTGCATGCCTAGAGAGTAGAGACCACCACCTTCTCAGATACTATTGAATATTTGATGAATCTTCTGCCCTGGCGATGGATCACAATCGACAGAAAAAAAAAATCTTTTAAAGTATATGTCTACACTCCTACTCTGGCCTGACTGATGATTTGCTGAATCTTTTCATGCACATACATACATGCATGCAGGAGGAATGGGTCATCTGCAGGATACTGCACAAGGCAGGCGACCAGTATAGCAAGCTGATGATGGTGAAGAGCCCCTACTACCTGCCCATGGCGATGGACCCTTCCAGCTTCTGCTTCCAGGAGGACCCAACCGGGCATCCCCTCCCGAACCCTAGCGGCTGCACCCCCTTCCACCACGGCCACCCCCACCATAGCATGCAGCCGCCGCCTCCATTGCCGCCGAGCAACCATGCTGGCAAGGCCGTCTTCACCGGAGCAGCAGCAGCCTGCTGCATGCAACAAGAGCCGGCAGACGGCAGCAACAGCGCCGTGCTGCCCATGCCGCCGTTCCCTCCCTTCACCCCCATCGTCGCCGGCAAGCCGGCGGCCCCGGCGCCGCCGCCCCAGGTTGTCAACGCCGGTCCACAGGAGCCACCGCCACCTACCTGGCTGGAGGCCTACCTGCAGCACACTGGTGGGATCCTTTATGAGATGGGTCCAACTGCAGCGCCCAGGGGCGCGTGATCCGGACCGTCGGTTCCTTAGGGTTACCCCCTACGTACGTACTGAAACACTGATGATCCATCGATTTACCTGGTCTGTAATGTGCGGTGAAGCTTAGTATTTCCAAGTGACATATTTCGACAAGCACTTGTAGTTATTAAAAAAGAGTTATCTTGTATTCGGTGGCATCATTAGTCGTCGTTGTTATGAAATCATGTGTGCTCATAACATGCATGAATGCATGCATTTGGATCATCGATCAGAGCAGTCCTGAGCTTGTACGTGCTACGTACTGTGCTCATGCATCCATCCACATGCAAAGTGGTGCTTGCTAAAATGTACTATCCAAATGTCTGTTTGTTTAGCATGCATGCAATGATATATCTATCGGAACTTGTTTCCTTTCTAGTTTACTGTTTGTTTCGGATTAATGTCAGATGTTCGGACT

>*Hv*CUC3 |MLOC_13932|*Hordeum vulgare*

TCTACACCTAGCTAGCTAGGTAGCGACACTGTGGGATAAAGCAATACCGATACAGGTGGTGAGGGAGAGAGAGAGAAGAGAGAGCAGGGACCATGGGCTAAACACAAACACTGTGAACTCTCTACGTTTCCGTGCTCAACTATATAGACCCTCCATTGGCCTCTTGCATCTACACACACAGACCACACCACCATTGCCAAGAGCAACCCATTTCACACTGACACTAGCTGCTTGGTCGCCGTGCCACAGCTCGCTTCTTCACCTAGCTCGTAGAGAGTGTGAGAGTGATCGTTGAAGGGCCGGCGCGCATGCACCAGCACCAACCAGCGGCGGCCATGGGCGACGCTCTGTGGGAACTGATCGGGGAGGAGATGGCGGCGGCGGAGGCGGCTGCCGGGGAGCACGGCCTGCCCCCAGGCTTCCGCTTCCACCCCACCGACGAGGAGCTTGTGACCTTCTACCTCGCCGCCAAGGTCTTCAACGGCACGTGCTGCGGCGGCGTGGACATCGCAGAGGTGGACCTGAACCGGTGCGAGCCGTGGGACCTCCCGGAGGCGGCGAGGATGGGGGAGCGGGAGTGGTACTTCTTCAGCCTCCGCGACCGCAAGTACCCCACGGGCCTCCGCACCAACCGCGCCACCGGCGCCGGCTACTGGAAGGCCACCGGCAAGGACCGCGAGGTGCTCAATGCCGCCACGGGCTCCCTCCTCGGCATGAAGAAGACGCTGGTCTTCTACAGGGGCCGCGCCCCCCGCGGCGAGAAGACCAAGTGGGTCCTCCACGAGTACCGCCTCGACGGCGACTTCGGCGCCGTCCGCCGCTCATGCAAGGTGCGTACTTATCCAGTTGGTTGCACGCCATTGCTCCATCCATGAACATTTTGTTACTCTTTTTGTTACACACATTCTCTAGCTTCTAGGCACTACCGCACTAGTCACTGTGTGATCTCGGCGTTGAAACATCCACAGTTGAGTGCTCAAGAACAAGTTACATGGTCGTTAGATGCGTGCGACATGATGTGCATTGAATGCGGCATGCGTGTGGTTGCATTGCATGCATACATAAGGTGACATATGCATGCAGATTGATTTGGAATATATGTTTATTATACTGTGGCCTAGTACACTTTTGTTCATTCATTCGATGCTTGTAGCATCATAAGCATGACGATGATGCTGTACAGAGTGCGTGGGTCGTTTCTGCTCAAAAATCTAGTTGCAGATTTGCACTGACACAGGATGTATATGGGCTAGCTAGCGATATATACTACACCATGTACTGGTACGTGTTTGTTCCTTCTCAGGGCTCACGCACTCTGCATGCATGGCTCGAAGATCTCCAACCGTCCTACTTCAGATCAGCAAATCTCCCATTGATATCTCTCTGTGTCATATGCTTTTATATAAAAAAAAAGCAATTTCGCCCATGAAGAATAAATGTACTCTCCGCTTGCAGAAGTTCAGGAGTAAATATGTAGATATAGATGTAGACATATAGATTCTCTACAGCATATCTGTCCTAGCTAGATCACAATCCATCCAAAAGAGAATATTATATTGATCTATACTTCTTTTTGTATGTTGATTTGTAAATATATCTGTGAGCGATATTTTGAACTTACAGTATATATGATATATATTTTTTGCAGGAGGAATGGGTGGTGTGCAGGATCTTCCACAAGGCGGTAGACCAGTACAGTAAGATGATGGAGATGAGAAACCCCTACTGCTACTTCCCCATGACCCCCCACCACCCCAGCTTCTTCCAGGACGCACCTCCTGTCCCCTTCCCAAACCCTAGTCAGCTCATCCCCTTCCACCATGACCTCCCACACCTGCAGTCCTCCCCATTAATACAGGGCCAGGCCAAGAACACAAGCAACAACAGCAATGGCGGCTTCCCAGCAGCAGCTAGCATCCAAGAGCAGCCAAACAGCAGCTGCAACCCAGCATATTTCCCTTTCCCTTCCTTCGCGTCCATCGTCAATGGCAAGGCAGGCCCACCGGCGCAGCCCGGGGTCAACGGCGGTCCACAGGAGCCACCGCCGACCTGGCTGGACGCTTGCCTGCAGCACAGCGCCTTCATGTACGAGATGGGCCCACCTGCAGCCACGAGGGGCGCATGATCCGGACCACTCGTTTCCTAGGGCTACCTTCCCCATCTCGATCGCTATGTTGCGGCTTTTGATTAGTCAAGTTGCGAGTGTGATCGATATTTAGACAAGCACTTGTAATTGTTTCCCCTTGTTCGTTCCCGACTTATCCTTTAGTTTCCGTAGGTTATGATCATATGTAGTGCTTTGCTTGCTTGTGCATAGGATCATTGGAGTAGTAGGAGACTAGCACTGGACTACTAATCTCGTGATCCATGCATGCACGTACTCGCATGCAATATGGTCCTATGTATTAATCTATATGTAATGAAGAACAACTCCTCGT

**B) CDS sequences**

>*At*CUC1 |AT3G15170|*Arabidopsis thaliana*

ATGGATGTTGATGTGTTTAACGGTTGGGGGAGGCCAAGATTTGAAGATGAATCCCTTATGCCACCTGGGTTTAGGTTTCATCCAACTGATGAAGAGCTGATCACTTACTATCTCCTCAAGAAGGTTCTTGACTCTAATTTCTCTTGTGCCGCCATTTCTCAAGTTGATCTCAACAAGTCTGAGCCTTGGGAGCTTCCTGAGAAAGCGAAAATGGGGGAGAAGGAGTGGTACTTCTTCACACTAAGAGACCGTAAATACCCAACGGGACTGAGAACGAACAGAGCAACAGAAGCTGGTTACTGGAAAGCCACTGGTAAAGACAGAGAGATCAAAAGCTCAAAGACAAAATCACTTCTCGGGATGAAGAAAACTCTTGTCTTTTACAAAGGCAGAGCTCCTAAAGGAGAGAAGAGTTGTTGGGTCATGCATGAGTATCGCCTTGACGGCAAATTCTCTTACCATTACATTTCCTCCTCCGCTAAGGATGAATGGGTTCTCTGTAAAGTTTGTCTGAAAAGCGGCGTAGTTAGTAGAGAGACGAACTTGATCTCTTCTTCTTCTTCTTCTGCCGTCACCGGAGAGTTCTCCTCTGCCGGTTCTGCAATTGCTCCGATCATCAATACCTTTGCGACGGAGCACGTGTCCTGTTTCTCCAATAACTCTGCTGCTCATACCGATGCGAGCTTTCATACATTCCTTCCCGCTCCACCGCCGTCACTGCCCCCACGTCAGCCACGTCACGTCGGTGATGGCGTGGCGTTTGGTCAGTTTCTGGATTTGGGATCATCGGGACAGATTGATTTCGATGCAGCAGCAGCAGCGTTCTTTCCGAATCTACCTTCTCTGCCTCCCACGGTTCTTCCTCCTCCTCCGTCATTTGCAATGTACGGTGGAGGCTCCCCCGCCGTGAGTGTGTGGCCGTTTACTCTCTGA

>*Br*CUC1 |Brara.C03560|*Brassica rapa*

ATGGATATGGATGTGTTTAATGGTTGGGAGAGATCGAGATATGAAGATGAAACCGTAATGCCACCTGGTTTTAGGTTTCATCCTACCGATGAAGAGCTCATCACTTACTACCTCCTCAAGAAAGTCCTTGACTCCAGTTTCTCATGTGCCGCCATTTCTCAAGTTAATCTCAACAAGTCTGAGCCTTGGGAGCTTCCTGAGAAAGCGAAGATGGGGGAGAAAGAGTGGTACTTTTTCACACTGAGAGACCGTAAGTACCCAACGGGCCTAAGAACTAACAGAGCAACAGAAGCTGGCTACTGGAAAGCAACTGGTAAAGACAGAGAGATCAAAAGCTCAAAGACAAACTCGCTTCTCGGGATGAAGAAGACTCTTGTCTTTTACAAAGGCAGAGCTCCTAAAGGTGAGAAAAGCTGTTGGGTCATGCATGAGTATCGCCTCGACGGAAAGTTCTCTTACCATTACATCACTTCCTCCGCTAAGGATGAATGGGTTCTCTCTAAAGTCTGTCTGAAAAGCAGTGTTGTCAGTAGAGAGACCAAACTGATCTCTTCTTCCGGCGGTGTCAACTGCTCCTCCTCCTCCTCCGCCGCTGGTTCGTTAATTGCTCCGATGATCGACGCCTATGCGACGGAGCACGTGTCCTGTTTCTCCAATACCTCTGCAGCTCATGCTGACGCGAGCTTTCCTCCTGCTTACCTTCCCGCTCCTCCTCCACCGTCTCTGCCACGTCAGCCTCGCTGCTTCGGTGATGACGTGGCGTTTGGTCAGTTTATGGATGTGGGAGCATCTGGACAGTTCAGCATCGACGCAGCGTTTTTACCGAATCTACCTTCTCTGCCTCCGACGGTGTTTACAACTCCTTCTCAGCCGTTCGGAATGTACGGTGGAGGCTCCGCCGTGAGTTCGTGGCCGTTTGCTCTCTGA

>*Dc*CUC1 |DCAR_020996|*Daucus carota*

ATGGACATCTTTTACCACAATCTGCAGAGCAATGCAGATGCGCAACTGCCTCCTGGCTTCAGGTTTCACCCAACAGATGAAGAGCTCATCACTTATTACCTTCTCAACAAAGTTCTTGATCACAACTTCACTTGCAGAGCCATTGCTCAAGTTGACCTCAACAAATGCGAACCATGGCACCTCCCTGAGAGAGCGAAGATGGGGGAGAAAGACTGGTACTTTTATAGCTTGAGGGACCGGAAGTACCCGACAGGGTTGAGGACGAATCGAGCAACCGAAGCTGGATACTGGAAGGCCACGGGGAAAGACAGGGAGATTTACAACTCAAAGACATCGTCTCTGGTGGGCATGAAGAAGACTCTTGTCTTCTACAGAGGCCGTGCTCCCAAAGGAGAGAAGACCAATTGGGTGATGCATGAATATCGCCTTGATGGCAAGCTGGCTTACCACTATCTTTCCACTAATTCTAAGGACGAGTGGGTGATATCCAGGCTGATGAAGAAAAGTGGTACTGCTGGCCCTGGTGACAAAACACCAACTTATGGCATGTACTCAGAAATGAGCTCTTCATCCTCCGCTTCTCTGCCACCTCTTCTAGACTCCACTCCCTTCACAGCAGCAACAACTGATCATCATCGCGTGATCGACTACTCCTACCTCGAGAAGGAGCACGTGTCCTGTTTCTCCAGATCCACAGCTGCACCTGGCGGCTTCAATTACCATACGACTCTATTTGACTGTGGCCTTCCTCCCCCGCTGATGGTCGACCACACCTCCTCCTCAACGTCATCCTCGTCTCAATTCCACGAAAACAATACAAATGGAGGGGAGAAGCTTCACTTGCCTAGCTTCTTTTTCCCCTCCATCACCCCGTCTCCGATTCATGGAGGCGTAGGAAGCATTTATGCATCTGACACGGGGAATTATTCAGTGCTGGAGGCTCAAAAGCCTGGCCTCACTGAGCTTGATTGCATATGGAGGGGCTCTTTTAATTGA

>*Sl*CUC1 |Solyc06g069710|*Solanum lycopersicum*

ATGGAGAATTATTCAGGAGTTGTTAAGGATGATGATCAGATGGAGTTACCACCTGGATTTCGATTTCATCCAACTGATGAAGAATTGATCACTCATTATTTGTCTAACAAAGTTGTGGATACTAATTTCGTTGCTATTGCTATTGGTGATGTTGATTTGAACAAAGTTGAACCTTGGGACCTTCCATGGAAGGCGAAAATGGGGGAAAAAGAATGGTATTTTTTCTGTGTGAGAGACAAGAAGTATCCAACAGGGCTGAGAACAAACAGGGCAACTGCTGCAGGGTATTGGAAAGCTACTGGAAAAGACAGAGAGATTTTCAGGGGAAAATCATTGGTTGGTATGAAGAAAACTCTGGTTTTCTACAAAGGGAGAGCTCCAAAAGGTGAAAAGACAAATTGGGTTATTCATGAATTTAGATTAGAAGGAAAATTGTCTCTTCAAAATCTGCCAAAGACAGCAAAGAATGAATGGGTGATTTGCAGAGTGTTTCAAAAGAGCAGTGGTGGAAAGAAAATCCACATTTCAGGGCTTTTGAAACTGAATTCTAATGAAAATGAAATGGGGAATTCATTTCTGCCACCATTGACAGATTCTGCTACTGCTACTGCTTCGAAATCCAGCCACGTGCACTGCTTCTCCAATTTTCTCACTGCTCAAAACAACTGTTTCCCTCTTCTGTCAAATCCAATGGATAGTTACCCTACAACTTCTCTTGTTCCAAATACATTTTCTTGTAACCAAATAGCTCCATTCACTACTACTAATAATCCAGCTTCATTTGGGGTTCAAGATCCTTCAATTCTTCTAAGGACTTCACTTGACAGCTATGGTCTGAATTTCAAGAAAGAGGACATTTTTAATGTACCCCAAGAAACAGGGGTAATTAGCACTGACATGAATACTGATATCACCTCAGTCGTATCAAATCTTGAAATGAAAAGAAGGTTTCTTGAAGATCAGGTGCCATCAGCAGGTATGGTTGGATTACAGGGTCTTGATTGTCTCTGGAGTTGCTGA

>*Eg*CUC1 |Eucgr.F01170|*Eucalyptus grandis*

ATGGAGAACTACCACCAGTACAGCAACCACCACCTCGTCAATGGCGACGGGCATTTGCCTCCGGGGTTCCGGTTCCACCCCACCGACGAGGAGCTCATCACGTATTACCTGCTTAAGAAGGTCCTGGACAGCAGCTTCACCGGGCGAGCCATCGCCGAGGTCGACCTCAACAAGTGCGAGCCCTGGGAGCTTCCCGAGAAGGCGAAGATGGGGGAGAAGGAGTGGTACTTCTTCAGCCTGCGGGACCGTAAGTACCCGACGGGGCTCCGCACGAACCGGGCGACGGAGGCGGGGTACTGGAAGGCGACGGGGAAGGACCGTGAGATCTACAGCGGCAAGACGGGGTCGCTGGTGGGTATGAAGAAGACCCTCGTCTTCTACCGGGGACGAGCCCCGAAGGGCGAGAAGAGCAACTGGGTCATGCACGAGTTCCGCCTCGACGGCAAGTTCGCCTACCCCTTCCTCTCCCGCTCCTCCAAGGATGAGTGGGTGATCTCCCGCGTTTTCCAGAAGTCCAGCAACAGCTGCGGCGCAGCCGCCCCTTGTGGTGGCAAGAAGACCCGCATGCCCCCCCACATGAACCTGTACCCGGAAATCGGTTCACCCTCGGTGTCTCTCCCACCACTGCTCGACTCCTCTCCTTACACCTCAACCACCGCGGGGTTCATCGACCGCGTCCCCATCTCTTACGACAGCTCAATCCCTAAGGAGCACGTGTCCTGTTTCTCCACGGCCGCTGCCGCCTCGTTGGCAACCCACAATTTCGGCAGCAACCCTAGTTTCCAACTGGCTCCTCCCACGGCCCCCCTGATCAATGCAACTGACCCGATGACCCGATTCTCCAGGAGCATCGGCGTCTCGGCTTTTCCGAGCCTGAGGTCCTTGCAGGAGAATCTCCAGCTGCCTTACTTCTTCTCCGGTAACCAGCATCTGAGTGGTGGAATTAATGATTTGGTCAGCTCGACTTCGAGCTCTCTGGGGAACTGGACGGCTCCGGATGATCAGAAGGCGGTGGACCTCGGTGGTCGAATGGGAATGGGCTCGTCGGAGCTTGACTGCATGTGGAACTTCTGA

>*Cs*CUC1 |orange1.1g017827m|*Citrus sinensis*

ATGGAAAACGTTTCTGCAGTTGGAAAGGAAGATGACCAGATGGATTTGCCGCCTGGTTTCAGATTCCATCCAACTGACGAAGAGCTTATCACTCACTATTTGTACAAGAAAGTTCTTGATGTTTGCTTCTCTTGTAGAGCTATTGGAGATGTTGATCTGAACAAAAATGAACCTTGGGAATTGCCTTGGAAAGCAAAGATGGGAGAGAAAGAATGGTACTTTTTCTGCATGAGGGATAGGAAATATCCAACTGGTTTGAGGACTAACAGGGCGACTGTATCTGGTTATTGGAAAGCCACGGGGAAAGACAAGGAGATTTACAGAGGAAAATCTCTAGTTGGAATGAAAAAGACTCTTGTTTTTTACAGGGGAAGAGCCCCAAAAGGGGAGAAATCAAGCTGGGTCATGCACGAATACAGATTGGACGGCAAATTCTCTGTTCATAGTCTCCCCAAAACTGCCAAAAATGAGTGGGTGCTTTGCCGGGTGTTTCAGAAGGGTTCTGGTGGGAAAAGGACTCATATTTCAGGGCTAGCGGGATTAGGGTCTTTTGGAAATGAATTGGGTCCTCCTGGCTTGCCACCATTAATGGATTCATCTCCTGATAATGGCAGCAAGACCATCAAATCTGTTGCCGATTCGGCTTACGTGTCCTGCTTCTCCAATTCTATTGATCTTCAAAGAAATCAAAAAACCACCACCACCATTGAAAATTTTTTCAACAATCCTCCTCCCATCTCTGTGTCTTCAAACTGTCCTGATGTTTTTCCAAGAATCCCACTCTCTTCAAACTCATTCTATTCTCCTTTATCGGTCCCGGTTCCATCACACGCGCAATTCCCAGGCTCTGTTTTCATGCAAGACCACTCAATCTTAAGGGCCTTAATTGAAAACCAAGGATCAAACATGAGCCAGAGTTTCAAAACAGAAAGGGAAATGATCAGTGTTTCACAAGACACAGGCCTCACTGCTGACATGAACCCTGAAATCTCCTCAGTTGTGTCCAACCTTGAAATGGTGAAGAGGCCATTTAATGATCATGATGCTCCTTCAACTTCAGCTGGACCAGTGGATTTTGACTGTTTTTGGAATTACTGA

>*Gr*CUC1 |Gorai.007G323900|*Gossypium Raimondi*

ATGGATAGTTACTATCATTTTGACAACGGTGATACTCATTTACCACCTGGCTTTCGTTTCCATCCAACTGATGAAGAGCTCATTACTTACTATCTCTTAAAGAAAGTTCTTGATAGTGGTTTTGCTGGTAGAGCTATAGCTGAAGTTGATCTTAACAAGTCTGAACCTTGGGAACTTCCTGGGAAGGCAAAGATGGGAGAGAAAGAGTGGTATTTCTTCAGTTTGAGAGATAGGAAGTACCCAACTGGGTTAAGAACTAACCGAGCTACTGAAGCTGGTTATTGGAAAGCTACTGGTAAAGATAGAGAGATTTACAGTTCAAAGACTTGTGCACTTGTTGGCATGAAGAAAACCCTTGTTTTTTATAGAGGAAGAGCCCCTAAAGGTGTAAAAAGCAATTGGGTCATGCATGAATATCGTCTTGAAGGCAAATTTGCTTACCATTATCTCTCCAGAAGCTCGAAGGATGAATGGGTGATATCCAGGGTGTTTCAGAAGAGTGGCTCCGCCAATGGTGCCACCAGCAGCACAGGCCGAGGAGCCAAGAAGACCCGGATGAACGCCTCCATTGCCGTCTATCAAGAGCCAAGCTCTCCTTCATCGATTTCCCTTCCACCTCTCTTAGATCCCACCGCAACTGCCTTTGCCACCACTGATCACGACAGCTTCTCTTACGACAATTATGTTCAATCCGAGCACGTGTCCTGTTTCTCCACCGTTACTGCTGCTACAGCCGCCTCTGCCACTGCCACCACCACAACCGCCCCATCGGCGTTCCACCCTGGTTTCGACAAAGCATTTCCACCTCCACCCCAAATGATCAACACCACTTTTGATCCCCTCGCGAAGTACTCAAGAAATGTGGGTGCTTCCGTTTTTCCAACCCTGAGGTCACTTGAGGAGAATTTGCAGCTCCCTCTCTTTTTCTCCCAGCCAACAATTGAAGCACCAACGCTTCACGGTGGCTCATCTGTCAACTGGGGAGCTTTTTCCGAAGAAATTAACGATGGGTCTGTTGGTGGTAACAAGATATCAATTGGTCCAACTGAGCTTGATTGCATGTGGACTTACTAA

>*Gm*CUC1 |Glyma.12G226500|*Glycine max*

ATGGACCACACCGAAGCTCACTTGCCACCTGGTTTTCGGTTCCACCCAACTGATGAGGAGCTCATAACTTACTACCTTCTCAAGAAGGTTCTTGACAGCACCTTCACTGGTAGAGCCATAGCTGAAGTTGATCTCAACAAGAGTGAGCCATGGGAGCTCCCTGAGAAAGCTAAGATGGGAGAGAAAGAGTGGTACTTCTTCAGCTTACGTGACAGAAAGTACCCAACTGGGTTAAGAACCAATAGGGCTACTGAAGCTGGGTACTGGAAAGCCACAGGGAAAGATAGAGAGATTTACAGCTCCAAAACTTGTTCTTTGGTGGGGATGAAGAAAACCTTGGTTTTCTACCGTGGTAGAGCTCCAAAGGGAGAGAAAAGCAACTGGGTCATGCATGAGTATCGCCTTGAAGGCAAATTTGCTTACCACTACCTTTCTCGCAACTCCGAGGATGAGTGGGTCATATCACGTGTGTTTCGGAAGAGTAACACCACACCGATCACCAATGGAGGCTCCACCATGTCCGCTTCTACTAACTCCAAGAAGACAAGAATTAACAACACCACCTCTCTTATCCATGAACCAGGTTCACCCTCTTCAGTTTTCCTTCCACCTCTTCTAGACTCTTCTCCCTACACCAACACCACCACCAACACCTTCACCGACCATCACAATAGTTCTTATGATAGTGCCACCAAAAAGGAGCACGTGTCCTGTTTCTCCACAATAGCTGCAGCAACAGCTGTTGTCTCCCCCAACAACAACTTCAACAATGCAAGCTTCGACCTTCCACCTTCTCAGCCTCTTGCAACCGACCCTTTTGCAAGGTTTCAGAGAAACGTTGGTCTTTCTGCTTTTCCAAGTTTGAGGTCTCTACAAGACAACCTTCAGCTACCTTTCTTCTTCTCCACGGCGGCGGCGCCTCCCTTCTCCGGCGGAGGCTCCGGCGACTTCCTCAGCTGGCCAGTGCCGGAGGATGGTGTTTCCAACATGCCACTGGGCGTGTCGGAGCTTGATTGCATGTGGGGCTACTAA

>*Pv*CUC1 |Phvul.011G160400|*Phaseolus vulgaris*

ATGGATAGTGGCTACTACAATCAGCGCCACCACCCTCACCTCGACAACAACAATGAACAACATTTACCTCCTGGCTTCCGGTTCCACCCCACCGATGAGGAACTCATCACATACTACCTCCTCAAGAAGGTTCTAGACAGCTCCTTCACTGGCCGAGCCATAGTTGAAGTCGACCTCAACAAGTGCGAGCCATGGGAGCTTCCTGAGAAAGCAAAGATGGGGGAGAAGGAATGGTACTTCTATAGCCTTCGTGACCGTAAGTACCCAACAGGCTTACGCACCAACAGGGCCACAGAAGCTGGTTATTGGAAAGCCACTGGAAAAGACCGAGAGATCTATAGCTCCAAAACCTGTTCCCTCGTTGGAATGAAGAAAACCCTTGTTTTCTATAGAGGAAGAGCTCCAAAGGGTGAAAAGAGTAACTGGGTCATGCATGAGTATCGTTTAGAAGGCAAATTCGCTTACCACTACCTCTCTAGAAGCTCCAAGGATGAGTGGGTGATATCGCGTGTGTTTCAGAAGAACAACACCGGCGGTGCCTCCACTGTGTCAGCCGCCGTAGCCACCGGCGGTTCCAAGAAAACGAGAATAAGCACATCAAACACAAGCAGCAACATGAGTCTCTGCCCCGAACCGGGTTCACCCTCTTCCATTTACCTCCCACCGCTTCTAGAATCTTCTCCATACGCCGCCAGCAGCAGCGCCGTCGCAACATTCAACGACCGCGAAAACTACTCCTTCGAAAGCGCCGCCGCCGCAGCCGCCGCCAACCAAAGGGAGCACGTGTCCTGTTTCTCCACTTTATCCACCGACGCTTCCGCCTTCAACCAGCTCGCTCCGCAGCCGGAGCCGCCACTCGACCCTTTCTCCCGCTTTCACAGGAACAACGTTGGACTGTCCGCCTTCCCATGTCTGAGGTCCTTGCACGACAACCTTAACCTCCCCTTCTTTTTCCCTCCCATGGTCCATGGCGGCACTGATGTAGCAAATTTTAGCGCCGTCGCAAACTTTCCGGCGCCGGAGGATCCGAGGGTGGTTGACGGCAGCTCTGGCATGTCGATTGTACCGTCCGAATTGGATTGCATGTGGGGCTATTGA

>*At*CUC2 |AT5G53950|*Arabidopsis thaliana*

ATGGACATTCCGTATTACCACTACGACCATGGCGGAGACAGCCAATATCTTCCACCGGGTTTCAGGTTTCATCCCACGGACGAAGAGCTCATCACTCATTACCTTCTCCGCAAAGTCCTCGACGGTTGCTTCTCAAGCCGTGCCATCGCAGAAGTTGATCTCAACAAGTGTGAGCCTTGGCAACTTCCCGGGAGAGCTAAGATGGGAGAGAAAGAATGGTACTTCTTTAGCCTCCGTGACCGGAAGTATCCGACGGGACTGAGAACTAACAGAGCAACTGAGGCTGGTTACTGGAAAGCTACCGGAAAAGACAGAGAGATCTTTAGTTCAAAGACTTGTGCACTTGTTGGGATGAAGAAGACTCTTGTCTTTTACAAAGGAAGAGCTCCGAAAGGAGAGAAGAGTAATTGGGTTATGCATGAATATCGTCTTGAAGGCAAATTCTCTTACCATTTCATCTCAAGAAGCTCCAAGGATGAATGGGTGATCTCTAGGGTTTTCCAGAAAACCACTTTAGCTAGCACCGGAGCCGTCTCCGAAGGAGGAGGAGGAGGAGGAGCAACTGTGAGCGTAAGCAGCGGTACTGGTCCATCTAAAAAGACGAAAGTACCCTCAACAATCTCAAGAAACTATCAAGAACAACCAAGCTCTCCTTCCTCCGTCTCACTCCCACCTCTCCTGGATCCGACCACTACCCTCGGCTACACCGACAGCAGTTGCTCCTACGACAGCCGTAGCACCAACACAACCGTCACAGCCAGCGCAATAACCGAGCACGTGTCCTGTTTCTCCACTGTCCCTACTACTACTACGGCCTTGGGCTTAGACGTTAACTCATTCAGCCGTCTTCCACCGCCGCTAGGGTTTGACTTTGACCCTTTTCCTCGTTTCGTTTCTAGAAACGTCTCGACTCAATCTAACTTCAGATCGTTCCAAGAAAACTTCAATCAATTTCCTTACTTTGGATCGTCTTCTGCATCGACTATGACCTCCGCCGTTAATCTGCCTTCTTTCCAAGGCGGCGGAGGCGTCTCCGGGATGAATTACTGGCTACCGGCGACTGCCGAAGAGAATGAGTCAAAGGTCGGTGTGCTTCATGC

TGGACTTGACTGTATTTGGAACTACTGA

>*Br*CUC2 |Brara.J00883|*Brassica rapa*

ATGGACATTCCACTTTACCACTATGACCACGGCGGAGACAGCCAATATCTTCCGCCAGGTTTCAGGTTTCATCCCACAGATGAAGAACTCATCACCCATTACCTCCTCCGCAAGGTTCTTGACGGTTGCTTCTCAAGCCGCGCCATTGCAGACGTTGATCTCAACAAGTGCGAGCCTTGGCAACTTCCCGGGAAAGCTAAGATGGGAGAGAAAGAATGGTACTTTTTCAGCCTCCGTGACCGGAAGTATCCGACGGGATTGAGAACGAACAGAGCAACGGAGGCTGGTTACTGGAAAGCTACCGGAAAAGACCGAGAGATCTATAGTTCAAAGACTTGTGCACTTGTTGGGATGAAGAAGACTCTTGTCTTTTATAAAGGAAGAGCTCCTAAAGGAGAGAAAACTAATTGGGTTATGCATGAATATCGTCTTGAAGGCAAATTCTCTTATCATTTCATCTCTAGAAGCTCAAAGGACGAATGGGTGATCTCTAGGGTTTTCAAGAAAACCGGTTTAGCCAATACCGGGGCCTCCGGGGGAGAAGCAAGTGCTAGCGTAAGCAGCTGTACCGGTGGGTCTAAAAAGACGAAAGTACCCTCAACCATCTCCACAAACTACCGTGAGCAACCAAGCTCTCCTTCCTCCGTCTCACTCCCTCCTCTCTTTGACCCCACCACAACACTCGGCTATACCGACAGCTGCTACTCCTACAACAGCCGTAGCAGCAATACAACCCTCACAGCCACTGCGATAACCGAGCACGTGTCCTGTTTCTCCACTGCCACGACTACTACTGCCTCAGGCTTAGATGTTAACGTTGACTCATTCAACCATCTTCTACCGCCTGCTCCGCCTGGGTTTGACCATTTTTCTCGTTTTGGCTCTAGAAACGTTTCAACTCTATCTAACATAAGGTCGTTCCAAGAGAACTTCAATCATTTCCCTTACTTTGGTTCGTCTTCTGCATCGACCATGACCCCCTCCGTTAATTTGCCTTCTTCCCACGGTGGCACCGGAATGAACTACTGGCTACAGACAACCGCGGAAGAGAACGAGACAAAGGCTGGTCTACTTAATGGTGGACTAGATTGCGTATGGAATTACTAA

>*Dc*CUC2 |DCAR_019571|*Daucus carota*

ATGGACCATTTCTACCAAAGTATGGAGAACAATGGGGATGCTCAGCTGCCTCCAGGCTTCAGATTTCACCCAACAGATGAGGAACTCATCACTTACTACCTCCTCAAGAAAGTCCTTGATCACAACTTCAGTAGCAGAGCCATTGCCCAAGTTGACCTCAACAAATGTGAACCATGGCACCTTCCTGAGAAAGCAAAGATGGGGGAGAAAGAGTGGTACTTTTACAGCTTGAGGGACAGGAAGTACCCAACAGGGTTGAGGACAAACAGGGCGACAGAAGCGGGGTACTGGAAGGCCACAGGGAAAGACAGGGAGATTTACAGCTCGAAGACTTCGTCTCTGGTGGGGATGAAGAAAACCCTAGTGTTCTACAGAGGCCGTGCTCCTAAAGGAGAGAAGACCAACTGGGTCATGCATGAGTTTCGCCTTGATGGCAAGCTTGCTTACCACTACCTCTCTACTACCTCTAAGGACGAGTGGGTCATCTCCCGGCTCTTCAAAAAAACCGGCGGCGCCACCGCCGGAGAAAAAAGACCAAGCTCCAGCATGAGCAGCCACTTTCACTCAGAAATCAGCTCATCTTCCTCCATCCCTTTCACACCACCTCCACCACCAGCAACAACAACTGATCACGTGATCACCTACGAGCACGTGCCCTGTTTCTCCAGTTCCGCCGCCCCCGGCGGCTTCAGCACTTACCACACCCTCTTCGACGGCGGCCTCCCTCCGCCGCTAATGGACCCCACCCCAATGCCGCCCTCCTCCACCTTCCCCAGCCTCAGATCACTAGAAGAGAATCTCCACCAGCCCAGCTTCTTCTTCCCGCCGGTCAACTACGACAACTTCCCGGCGATGGAGACACCGAAGCCGGGACTCACTGAACTCGACTGCATTTGGAGACCCTCGTTTAATTAA

>*Eg*CUC2 |Eucgr.B00529|*Eucalyptus grandis*

ATGGAGAACATGGCTAGGCTCGGGAAGGAAGACGATCAGATAGAGTTGCCGCCGGGGTTCAGGTTCCACCCGACGGACGAAGAGCTCATCACCCATTATCTGCAGAAGAAGGTGGGGGACACTGGCTTCTCCGCCAAAGCCATCGGAGAAGTGGATTTGAACAAGTCCGAGCCCTGGGATTTGCCTTGGAAGGCGAAAATGGGGGAGAAGGAATGGTATTTCTTCTGCCTGAGGGACAGAAAATACCCGACTGGTTTGAGGACCAACAGAGCCACCGAATCTGGTTACTGGAAGGCCACGGGGAAAGACAAGGAGATCTACAGGGGAAAATCTCTGGTTGGTATGAAGAAAACCTTGGTTTTCTACAGAGGGAGGGCTCCAAAGGGGGAGAAGACGAATTGGGTCATGCATGAATACAGATTGGAAGGAAAACTCTCTCTGAATTATCTCCCCAGGGCTTCGAAGAACGAGTGGGTCATTTGCAGGGTCTTCCAGAAGAGCTCTGGTGGGAAGAAAATCCACATCTCGAGCCTCGTGGCGGCGGGGTCTCTCGAGAACGAAATGAGCTCCGGCTTGCCGCCGTTAACGGATTCCTCTCCTCACGATTCGAAGACGGAATCCAACCCCGGATCGGCTTACGTGCCCTGCTTCTCCAGCCCAACAGAGTTCGAAAGGAACAAGGAAAACACGAACAATTACTTCAACAATCCCATGTTCCCCATCTCCTCGAACCCCACGAACACCACCCCCAAAATCTCGCTCTTGAGCCCAGTGTACCCTCACCAGGCCATCCCCGTCCCAGCCAATTGGCAACACCCGGGGGGCTCCGTCTTCATGCCCGAGCACTCGGTCCTCAGGGCTCTGCTCGAGGGCACCGGGCTGAACGCGAGGCAGAGCGCGAGGGCGGAGCGGGAGGCGATCAGCATCTCCCAAGAGACAGCGCTGACCAACGACTTGAACACCGAGATCTCCTCCGTCATGCAGGATTTCGAAATGGGGAGGAGGCAGTTCGAGGATCAGCAGCAAGTTCCATCGACCTTAGCTGGACCAATGGACGTGGACCTCCTCTGGAACTATTCAAGTTAG

>*Gm*CUC2 |Glyma.13G274300|*Glycine max*

ATGGACAACTCTTCCTACCACCACTTGGACCACACTGAAGCTCACTTGCCACCTGGTTTTCGGTTCCACCCTACAGATGAGGAGCTCATAACTTACTACCTTCTCAAGAAGGTTCTTGACAGCACCTTCACTGGTAGAGCCATAGCTGAAGTTGACCTCAACAAGAGTGAGCCATGGGAGCTTCCTGAGAAAGCTAAGATGGGAGAGAAAGAGTGGTACTTCTTCAGCTTACGTGACAGGAAGTACCCAACTGGGTTAAGAACCAATAGGGCTACTGAAGCTGGTTACTGGAAAGCGACTGGGAAAGATAGAGAGATTTATAGCTCAAAAACTTGTTCTTTGGTGGGGATGAAGAAAACCTTAGTTTTCTACCGTGGTAGAGCTCCAAAGGGAGAGAAAAGCAACTGGGTCATGCATGAGTATCGCCTTGAAGGCAAATTTGCTTACCACTACCTTTCTCGCAACTCCAAAGATGAGTGGGTCATATCACGTGTGTTTCAGAAGAGTAACACCGCCACCAACAATGGAGGCTCCGTCATGTCTGCTTCTAGTAACTCCAAGAAGACAAGAATGAACAGCACCACCTCTCTTATCCATGAACCAAGTTCACCCTCCTCAGTTTTCCTTCCACCTCTTCTAGACACTTCACCCTACACCAACACAGCTAACTTCACCGACCGTCACAATGGTTCCTATGACAGCATCACCAAAAAGGAGCACGTGTCCTGTTTCTCCACAATAGCTGCAGCAACAACTGCTGTTGTCTCCCCCAACAACTTCAACAATGCAGGCTTTGACCTTTCACCTTCTCAGCCTCTTGCAACCGACCCTTTTGCCAGGTTTCAGAGGAACGTTGATTTTTCTGCTTTTCCAAGTTTGAGGTCACTACAAGACAACCTTCAGTTCCCTTTCGTCTTTTCCACGGCTGCACCGCCCTTCTCCGGCGGCGGTTCCGGCGACTTTCTCAGTTGGCCGGTGCCGGAGGAGCAGAGGCTGATAGATGGTGTTTCCAACATGCCACTGGGAGTGTCGGAGCTTGATTGCATGTGGAGCTACTAA

>*Gr*CUC2 |Gorai.013G171300|*Gossypium raimondi*

ATGGATAGTTACCATCATTTTGACAATGGTGAAACGCATTTACCTCCAGGTTTTCGTTTCCATCCAACTGATGAAGAGCTCATTACATACTATCTGGTGAAGAAAGTTCTTGATAGGAGCTTTACTGGTAGAGCCATAGCTGAAGTTGACCTCAACAAGTGTGAGCCTTGGGAACTTCCTGACAGGGCAAAGATGGGAGAGAAAGAGTGGTACTTTTTTAGCCTAAGAGATAGGAAGTACCCAACTGGGTTGAGAACTAACCGAGCTACTGAAGCTGGTTACTGGAAAGCTACTGGGAAAGATAGGGAGATTTATAGTTCAAAGACTTGTGCACTTGTTGGAATGAAGAAAACTCTGGTTTTCTATAGAGGAAGAGCTCCTAAAGGAGAGAAAAGCAACTGGGTCATGCATGAATATCGCCTTGAAGGCAAATTTGCTTACCATTATCTCTCTAGAAGCTCCAAGGATGAGTGGGTAATATCCAGGGTCTTTCAGAAGAGCAGCGGAGGAGCCAAGAAGGCCCCCATGAGCGCCGCTTCCATGGTGCTCTACCAAGAACCAAGCTCACCTTCCTCGGTCTCTCTTCCACCGCTCCTGGATACCACCAATGCTACTGGCAGTGGTACCGCTACCGGTGCTTCCCTCACTGACCGTGACAGCTGCTCTTACGACAGCCATAACCAATCCGAGCACGTGTCCTGTTTCTCCACCATTGCTGCCACCTCGTCAGCCACCCTTCCTGGCTACCACAGCGGATTCGACCTTGCATTGCCAACCCCACCCCAGATGAATAACAGTTTTGATTCAATTGCAAGGTACACAAGAAATGTGGGTGTTCCAGTGTTTCCAAGCTTGAGGTCTCTCGAGGAGAATTTGCAGCTCCCTTTCTATTTCTCGGAGCCAACATTGGCGGGGGCGGCACCACCACTTGACGGTGGTTCATCAGCGAACTGGGGAGCTGTTTCTGAGGAAGGAAACAGTGGTTCTGTTGCTGATGGCAAGATGTCCAATATAGGTCCTACTGAGCTTGATTGCATGTGGACTTACTAA

>*Cs*CUC2 |orange1.1g047710m|*Citrus sinensis*

ATGGAGATCACCTACAATTACTTTGACAACAGTGATGCACATTTGCCTCCTGGCTTTAGGTTTCACCCAACTGATGAAGAACTCATCACTTACTACCTTCTCAAGAAAGTTCTTGACTGCAACTTCACTGGCAGAGCCATTGCCGAAGTTGACCTCAACAAGTGTGAGCCCTGGGAGCTTCCTGCTAAGGCAAAGATGGGCGAGAAAGAGTGGTACTTCTTTAGCCTGAGAGACAGGAAGTACCCAACTGGGCTGAGAACTAACAGAGCTACGGAGGCTGGTTACTGGAAGGCCACTGGGAAGGACAGAGAGATTTACAGCTCCAAGACTTGTGCTCTTGTGGGCATGAAGAAGACTTTGGTTTTCTACAGAGGCCGAGCTCCTAAAGGAGAGAAAAGCAACTGGGTTATGCATGAGTATCGCCTGGAAGGCAAATTTGCTTATCAATATCTCTCCAGAAGCTCCAAGGATGAATGGGTGATTTCAAGAGTATTTCAAAAGAGCAGTGGAGCCATCGCCACCGCGGCTGCCGTCGCAAACGCCGTCAAGAAAAGTCGTTTGAGCTGCACCATTTCGTCATCTTCAACCTTCAATCACTCGTATCCGGAACCCAGCTCCCCTTCATCAGTTTCTCTTCCTCCTCTCCTTGATCACCCCACCATTGCTGCCGCTGCTAACGCCACCACTGCCCCCAATGACAGCTGCTCGTATGATGAAAGCCACGCTCCTTCTGATCAGCACGTGTCCTGTTTCTCCACCATTGCAGCCGCCGCAGCCGCAGCCGCAGCTTCGGCAGCCACTGCCACCACATTCAACACCAGCTCCTCAGCTTTTGACTTCACTACAGTACCAGCGCCTGTTATCAATGCTGATGCTGGTGCCGGCGCTGCTTGTGACCCGTTTGCTCGTTTTGGAAGAAACAATGTTGGCTTGAATGCTTTCCCTAACTTGAGGTCTCTGCAGGAGAATCTTCAGCTTCCTTTCTTCTTCGCACCACCTGCTTCTTCAGTTGCGCCTCCTCCCTTTCAGGGTGGCGGTGGTGGGTCAAACTGGTCAACGGTGATGCAGGACATCGGCGGTGGCGGTGGTGTTGTTGGTGGTGGCGGCAGGTTGAATGTGGGTCCCACTGAGCTTGATTGCATGTGGACTTACTGA

>*Pv*CUC2 |Phvul.005G074500| *Phaseolus vulgaris*

ATGGACTCCTCCTACCACCACTTGGACCACACTGAAGCTCACCTGCCACCTGGCTTTAGGTTCCACCCCACTGACGAGGAGCTCATAACGTACTACCTTCTCAAAAAGGTTTTAGACAGCACCTTCACTGGTAGAGCCATAGCTGAAGTAGACCTGAACAAGAGTGAACCATGGGAGCTCCCAGAGAAAGCTAAAATGGGTGAGAAAGAGTGGTACTTCTTCAGCTTACGTGACAGGAAGTACCCAACTGGGTTACGAACCAATAGGGCTACTGAAGCTGGTTACTGGAAAGCCACTGGGAAAGATAGAGAGATTTACAGCTCCAAGACCTCTTCTTTGGTCGGGATGAAGAAAACCTTGGTTTTCTACCGTGGTCGAGCTCCCAAGGGGGAGAAAAGTAACTGGGTCATGCATGAGTATCGCCTTGAAGGCAAATTTGCTTACCACTACCTTTCTCGCAACTCCAAGGATGAGTGGGTCATATCGCGCGTGTTCCAAAAGAGCAACACATCCAACGGCGGCTCCGCCATGTCTGCTTCAAGTGGCTCCAAGAAAACAAGAATGAATACCACCAACAGCTCTCTCTGCCCAGAACCAAGTTCACCCTCTTCAGTTTACCTTCCGCCTCTTCTAGACTCTTCACCGTACGCCAACACAACCACCGCGGTCAACTTCACTGGCCGTAACAACTGTTCCTATGACAGCACCACCAAAAAGGAGCACGTGTCCTGTTTCTCCACAATCGCTGCAGCCACCGCTGCTGTTGTCTCCCCAAACAACTTCACCAATGCAAGCTTCGACCTTCCACCATCTCAGTCTCTTGGAACGGATCCCTTCGCTAGGTTTCAGAGAAACGTTGGCGTATCTGCCTTCCCAAGTTTGAGGTCACTGCAAGACAACCTCCAGTTACCGTTCTTTTTTCCTCCAGCAGCACAGCCCTTCTCCGTCAGTGGCACCGGTGATCTCCTCTGGCCGATGCCGGAGGAGCAAAGGCTGGTTGATGCGGCATCCAACGTTCCACTGGGGGTGTCGGAGCTTGATTGTATGTGGGGATACTAG

>*Sl*CUC2 |Solyc07g062840|*Solanum lycopersicum*

ATGGAGATTTATCATCAGATGCAGTTTGATTGCGGTGATCCGCATTTACCACCGGGGTTTCGGTTTCATCCAACTGACGAAGAACTTATTACTTACTACTTGTTGAAGAAGGTTCTGGACTGCAACTTCACTGCTAGAGCTATTGCTGAAGTTGATCTCAACAAATGTGAACCTTGGGAACTTCCTGGGAAAGCGAAAATGGGAGAAAAAGAATGGTATTTCTTCAGTCTACGTGATCGGAAGTATCCAACAGGGCTGAGGACTAACAGAGCTACTGAAGCGGGTTACTGGAAAGCTACTGGAAAAGATAGAGAAATTTTCAGTTCAAAAACATGTGCACTTGTTGGTATGAAGAAAACCCTAGTTTTTTATCGAGGAAGAGCACCAAAAGGAGAAAAAAGTAACTGGGTTATGCATGAATATCGCCTTGATGGCAAATTTGCCTATCATTATATCTCCAGGAGTTCGAAGGACGAGTGGGTTATCTCGCGGGTCTTTCAAAAAAGCACCGGTTCTAATGGTGCCGCTACTTCAACTGGTGGCGGCAAAAAAAGGCTAAGTTCAAGTATAAACATGTACCAGGAAGTGAGTTCACCGTCTTCCGTCTCTCACCTTCCGCCGCTCCTCGATTCCTCTCCGTATAGCACTACCGCCACTTCCGCCGCAGCTATCGTAATCGGCGACCGCGATCGTGATCATAGCTTCAAGAAGGAGCACGTGCCCTGTTTCTCCACAACTGCTACTGCTACAATAACTGCACAGAGTCTAACTTTCGATCCAACTTCTGTCTTCGACATTTCATCAAACACCTTGCATGCACTACAGCCAACTCCAAGTTTCGCTTCTATTTTGGACTCTTCTCCATCTAATTTCACTAATTACACAAGGAATTCAACTTTTCCAAGCTTAAGATCACTCCATGAGAATCTCCAGCTTCCGTTATTCTCCGGCGGAACCTCCGCCATGCACGGCGGATTTTCTAATCCGATGGTTAATTGGACCGTGCCGGAGACTCAGAAAGTTGAACAGTCTGAACTTGACTGTATGTGGAGCTACTGA

>*Ss*CUC2 |Sspon.07G0020380-1A|*Saccharum spontaneum*

ATGGAGCGGTTCGGCGTGCTGGGCACGCGGCTGGGCCTGGACGGCGTCGTCGGCGGCGGCGGAGGCGAGCTGCCGCCGGGGTTCCGGTTCCACCCGACGGACGAGGAGCTCATCACCTACTACCTCCTCCGCAAGGCCGTGGACGGCAGCTTCTGCGGCCGCGCCATCGCCGAGATCGACCTCAACAAGTGCGAGCCATGGGAGCTCCCGGACAAGGCGAAGATGGGGGAGAGGGAGTGGTACTTCTACAGCCTCCGCGACCGCAAGTACCCGACGGGCCTGCGCACCAACCGCGCCACGCTGGCCGGCTACTGGAAGGCCACCGGCAAGGACCGCGAGATCCGCAGCGCCCGCTCCGGCGCGCTGGTGGGCATGAAGAAGACGCTCGTCTTCTACCGCGGCCGCGCCCCGAAGGGACAGAAGACGCACTGGGTCATGCACGAGTACCGCCTCGAGGGCACCTACGCCTACCATTTTCTCCACAGCTCCACAAGGGATGAGTGGGTGATCGCCAGGGTGTTCCAGAAGCCCGGCGAGGTCCCACCGGCCCGCAAGCACCACCGCCTCGGCGGCCTCAGCAGCGCCGGCGGCGGCGAGTCCTGCTTCTCGGACTCCACCTCGGCCTCCATCGGCGGAGGCGGCGGCGGCGCATCGGCGTCGTCCGCGCCTCGCCCGCTGCCGCTCACGGTCACGGACGCCTCCTCGCTGTCGCTGTTCGCGTCGGCCGCCGCGGCCAATGCCGCCGACGGCGACAGCAGCTCCTACTGCGGCGGAGCCGCGAACAACGCCAACAATGGCAACAACCTGGTCACCGGCCGTGAGCTCGTGCCCTGCTTCTCCACTAGCACCACCACCGGCGCCGGCGGCCTGGATGCCGCCGCGCTCGGCATCGGGCAGCCGTACAACGCAGCAGTCCCGCTGCCGCTGGCCTTCGAGCCGCCGCCGCCGACTCCGGCCTTCTTCCCGAACCTGCGTTCGTCCCTGCAGCTGCAGGTGCAGCAGGACAACAACCTCGAGCTGCCACTGTTCCTCTCGGCAGCCGGCGGCCTGTCCGCTGCGACGCTGGGAATGGGGTCGATGGGCGGCGGGGCTCTCCACCACTGGCCCCTCGCCGGCATGGAGGTCAAGGTCGAGGGCCGCTCCGCGCCGCCGCAGATGGCTGTCGGCCCCGGCCAGCTCGATGGCGCCTTCGGCTGGGGCTACTAG

>*Ac*CUC2 |Aco020094|*Ananas comosus*

ATGGAAAGCTACGCGGCGCAGCACCACCGTTTCGACAGCGGCGATGCGCAGCTCCCGCCGGGGTTCCGCTTCCACCCGACGGACGAAGAGCTGATCACTTACTACCTCCTGAAGAAGGTCCTCGACGGCGGCTTCACCGGTCGGGCCATCGCCGAGATCGACCTCAACAAATGCGAGCCCTGGGAGCTCCCTGAAAAGGCCAAGATGGGGGAGAAAGAGTGGTACTTCTTCAGCCTCCGCGACCGCAAATACCCGACGGGGCTGCGGACGAACCGGGCGACGGAGGCCGGGTACTGGAAGGCGACGGGGAAGGACAGGGAGATATTCAGCTCCCGCACGGGCTCGCTCGTCGGGATGAAGAAGACACTGGTGTTCTACCGAGGGAGGGCCCCCAAGGGGGAGAAGAGCAACTGGGTCATGCACGAGTATCGCCTCGATGGGAAGTTCGCCTACCACTTTCTCTCCAGATCCTCCAAGGACGAATGGGTGGTTTCTCGGGTGTTCCAGAAGATTGGCGGCGGCAAGAAGACGCGCCTCGGCCTGGCTGGCCCTTCCAATTCTGATGCCGCAGGCGGTGGCGTCGGCTCGCAGTCCTCCAGCTCGCTCCCGCTGCTGCTCGATTCCTCGCCCTTTGCCGGCGCCGCCTCCTCATTCGCCTCTGCAGATCGCGAGAGCTGCTCCTACGAGAGCACTGATAGGGAGCCCGTGCCCTGCTTCTCCACCACCGCATCCCACCTCCTCGGCAACGAGGCCACACCGCCACCGCTCTTCGGCCGGGTGGGCACCACTGCCGCTGCTACCGCTGCTACTAATGTTAACGTTGGGTTGGCGTTTCCGTGCCTCCGCTCGCTCCAGGAGAACCTCCAGCTCCCGTTCTTCCTCTCCGGCCTCGCGCCGCCGCTGCCGCCGCTGCCCGGTTCTGGCCGACCTCGAGCGGAAGGCGGAGCTGGGCAGCAGGGTCCCCCACCAGATGATGATGCCGGTGGGGTCCACCGAGCTGGATTGCCTCTGGACGTTCTAGCTTGTTTAACAACCGATCTCGTCCTTCCAACGCATGCTATATGTTTGACTTATGATTACGACTAA

>*Os*CUC2 |LOC_Os06g23650|*Oryza sativa*

ATGGAGCGGTGCAGCGTGCTGGGGCTGGGCGGTGGCGGGGGCGGGGGCGGGCGGCTGGACGGCGAGCTGCCGCCGGGGTTCCGGTTCCACCCGACGGACGAGGAGCTGATCACCTACTACCTGCTGCGGAAGGTGGTGGACGGGAGCTTCAACGGGCGCGCCATCGCGGAGATCGACCTGAACAAGTGCGAGCCGTGGGAGCTGCCGGAGAAGGCCAAGATGGGGGAGAAGGAGTGGTACTTCTACAGCCTCCGCGACCGCAAGTACCCCACGGGACTCCGCACCAACCGCGCCACGGGCGCCGGCTACTGGAAGGCCACCGGCAAGGACCGCGAGATCCGCAGCGCCCGCACCGGCGCCCTCGTCGGCATGAAGAAGACCCTCGTCTTCTACCGCGGCCGCGCCCCCAAGGGCCAGAAGACCCAGTGGGTCATGCACGAGTACCGCCTCGACGGCACCTACGCCTACCACTTCCTCTCCTCCTCCACCCGGGATGAGTGGGTGATAGCCAGGATCTTCACCAAGCCCGGCGTGTTCCCCGTCGTCCGCAAGGGCCGCCTCGGCATAAGTGGCGGCGGCGGCGACACCTCGTGCTTCTCGGACTCCACCTCCGCCTCCGTCGGCGGCGGGGGCGGCACCTCCGCCTCGTCGGCGCTGCGCGCGCCGCTGGCTGAGGCCTCGCTGTTCGCCGCCGCCGCGGCGCCAGCCGTTGACGGCGCCGACAGCAGCAACTACGGCGGGGGCGGCGGCGCCGGCAGCGCCACCGCCACCGCCAACTTGGTCACCGGCCTTGAGCTCGTGCCCTGCTTCTCCACCACAGCCCACATGGATGCCTCGTTCGGCACCGGGCAGTACAACCCGGCCCCGCTGGCCGTCGAGCCGCCGCCGCCGCCGCCGGCCTTCTTCCCGAGCCTCCGCTCGCTGCAGGAGAACCTGCAGCTGCCGCTGTTCCTCTCCGGCGGCATGCAGGCGGGCGTGTCGTCGCAGCCGCTCAGCGGCGGCGGGGCCTTCCACTGGCAGTCCGGCATGGACGTCAAGGTCGAGGGCGCCGTCGGCCGCGCGCCGCCGCAGATGGCCGTTGGCCCCGGCCAGCTCGACGGCGCCTTTGCATGGGGCTTCTAG

>*Bd*CUC2 |Bradi1g41712 |*Brachypodium distachyon*

ATGGAGCGGTACGGGCTGCTGGGCACGCGTGAGGAGGAGCTGCCGCCGGGGTTCCGTTTCCACCCGACGGACGAGGAGCTCATCAGCTACTACCTCGCGCGCAAGGTGGCGGACGTGAACTTCTCGGGCGCCCGCGCCATCGCGGAGATCGACCTCAACAAGTGCGAGCCGTGGGAGCTCCCGGACAAGGCCAAGATGGGGGAGAAGGAGTGGTACTTCTACAGCCTCCGCGACCGCAAGTACCCGACGGGGCTGCGCACCAACCGCGCCACGGGGGCCGGATACTGGAAGGCCACGGGGAAAGACCGCGAGATCCGCAGCGCGCGCACCGGCGCACTCGTCGGCATGAAGAAGACCTTGGTGTTTTACCGTGGACGCGCCCCCAAGGGGGCCAAGACCCAGTGGGTCATGCACGAGTTCAGGCTCGACGGCAACTGCGCCTACCACTTCTTCTCCAACAACAACGCCACAAGGGACGAGTGGGTGATAGCCAAGATCTTCGTGAAGCCCGGCGCGCTCCCCGCCGCCCGCAACAAGCTCGCCCGCTTCGGCCTGCAGGGCAGCACCGGCGGCGCCGACACGTCCTGCTTCTCCGACTCCACCACCTCCGTCTCCATCGGCTGCGGCGGCGGGGGCGGGGATACCACCACCAACACCAGCTCGTTATTCGCGGCCGCGGCCGACGGCGAGAGCAGCTCCTGCGGCGGCGGCAACAACAACAACTGCGGCCGTGAGCTCGTGCCCTGCTTCTCCACTGGCGCCCACATGGACGCCACCCTCCTCGGCATCGGCCAGTACGACCCGGCCCCGCTGGCCATGGAGCAGCCGCCGGCCTTGTACCAGCTGAGCGCGGCCCGCTCTGTGCAGGACAACCTCCTGTTCCTCTCCGGCGGCGGCCTGCAGTCCGGCCTAGTGTCCCCGCTTGGCGTCGGCGGAGGGGCTTTTCAGTACTGGCCGACGTCGTCCGGCTACGACATGAAGCCCCCGCAGATGGCCGTCGGCCCCGGCCAGCTTGACGGCTCCTTCGGCTGGGGCTTCTAG

>*Zm*CUC2 |GRMZM2G139700_P01|*Zea mays*

ATGGAGAGGTTGGGCGTCGGCGTCGGCGTCGGCGAGCTGCCGCCGGGGTTCCGCTTCCACCCGACGGACGAGGAGCTGATCACCTACTACCTCCTCTGCAAGGCCGTGGACGGCGGCTTCTGCGGCGGCCGCGCCATCGCGGAGATCGACCTGAACAAGTGCGAGCCATGGGAGCTCCCGGACAAGGCGAAGATGGGGGAGAAGGAGTGGTACTTCTACTGCCTCCGCGACCGCAAGTACCCGACGGGCCTGCGCACCAACCGCGCCACGGCGGCCGGCTACTGGAAGGCCACCGGCAAGGACCGCGAGGTCCGCAGCGGCCGCAGCGGCGCGCTGGTGGGCATGAAGAAGACGCTCGTCTTCTACCGGGGCCGCGCCCCCAGGGGCCAGAAGACGCGCTGGGTCATGCACGAGTACCGCCTCGACGGCACCTACGCCTACCATTTCCTTCCCGGCTCTACGAGGGACGAGTGGGTGATCGCGAGGGTGTTCCAGAAGCCAGGCGAGGTCCCATGCGGCCGCAAGCACCGCCTGGGCGGCCCCAGCGCCGCCGCCGGCGAGTCCTGCTTCTCGGACTCCACCACCTCGGCCTCCATCGGCGGCGGCGGCGGAGGAGGAGCGTCCGCGTCGTCTCGCCCGCTGCTCACCGTCACGGACACTTCCTCGCCGTCGCTGTTCGTGGCCAACGCGAACGCCGCCGCCAGCAACAACAACGGCAACCCGGTCACCGGGCGAGAGCTCGTGCCCTGCTTCTCCACTACCGCCAGTCCCCTGGAAGCCGCGGCGCTCGGCGTCGTCGGGCACCCGTACAACGCGGCCCCGCTGCGTCTGGGCTTGGACTTCGAGGCGCCGTCCCCGGGCTTCGTCGTCCCGAACCTGCGTTCCCTGCAAGTGCAGGACGACGGCGGCCTGCCGCTGTTCCTCTCGGCAGCAGCAGGCGGCGGCATGTCGTCCGCGACGCTGGGAATAATGGGGTCGCTGGGCGGGTCTCTCCACTGCCCGCCCCACGCCGGCATGGATGTCGTCAAGGTCGAGGGCCGCGCCGCGCCGCCGCAGATGGCTGTCGGCCCCGGCCTCCTCGATGGCGCCTTCGCCTGGGGCTTCTAG

>*Hv*CUC2 |MLOC_65286|*Hordeum vulgare*

CCTCCCCCAACCACGCACGCGGCCGGCCAGCGGCACTATAAAACAAGGGCGCGCCTCCACTACCAGCGAGACAGCCGCTCCCCACCACCACTCGCTCTTCGATCACTTCTCTCGCCACAGGTCAGTGCGAGGGCGCGGCCGGGACCGAGATGGAGCGGTACGGTTCTCTGGGCATGCGGCTGGACGGCATCGGCGGCGGGGGCGGCGAGCTGCCGCCCGGGTTCCGCTTCCACCCGACGGACGAGGAGCTCATCACCTACTACCTCCTCCGCAAGGTGGTTGACTGCGGCTTCTCCGGCGCCCGCGCCATCGCCGAGATCGACCTCAACAAGTGCGAGCCGTGGGAGCTGCAGGACAAGGCCTGCAAGGCCACGGCGGAGAAGGAGTGGTACTTCTACAGCCTCCGCGACCGCAAGTACCCCACGGGCCTGCGCACCAACCGCGCCACCGGCGCCGGCTACTGGAAGGCCACCGGCAAGGACCGCGAGATCCGCAGCGCCCGCAACGGCGCGCTCGTCGGCATGAAGAAGACGCTCGTCTTCTACCGGGGCCGCGCCCCCAAGGGCCAGAAGACCCAGTGGGTCATGCACGAGTTCCGCCTCGAGGGCGTCTACGCCTACCACTTCCTGCCCAACAACACCACAAGGGATGAGTGGGTGATCGCGAAGATCTTCGTGAAGCCCGGCGCGGCGCCCCCCTCCCGCAAGGCTCGCTACGGACTCAGCAGCGCCGGCGACACGTCGTGCTTCTCCGACTCCACGTCCGTCTCCATCGGCGGCGGGGGCGGCGCCTCCGCCTCGTCCGCGCCGCGCCAGCAGCTCCCGGATACCAGCTCGCTGTTGGCCGCGGCTCACGCTGCCGCTGACGGCGAGAGCAGCTCCTACGGTGCCACCGGCAACAACAACAACGCGGCCGGCAACTGCCGTGAGCTCGTGCCCTGCTTCTCCACCGCCCAGATGGATGCCACCCTCCTCGGCATCGGGCAGTACGAACCGGCTTCGCTCGCCGTCGAGCAGCCGTTGGCCTTCTTCCAGGGCCCCCGCCTGCACCAGGCGGCGGACAACCTCAGCCTGCCGATGTTCCTCCCCGGCGGGCTGCAGTCCGGCGTCTCCCCGCTCGGCATGGGCGGAGGGGCCTTCCAGCACTGGCCGTCCTCCGGATACGAGGTGAAGCTGGAGGGCAGCCGCGCGCCGCCGCAGATGGCCGTGGGACCTGGCCAGCTTGACGGCGCCTACGGCTGGGGCTTCTAGACCAGTATGCATGCATGCCCTTAATCGTGTGCTGATTATGCTCTCTCATTCCAGCTTGCTTATCAGTATGCTTAGACGCATGTGTCCTTTGCTAAGTAGCTAGCTATATCTCGATCATTATGCTGCAGTAAGTTTCGATTATTAATTCATGCTCTCTGATCGTCGCGTGTTACTACTGCAACAGCAGGGCACACGACTTCACGAGCAAAGTACTGGATCACTAGCTAAATTACCTAGCTTAGATTATTAGCTAGTCGTATGTTGTTCTATTAGTATGTATTGTTAGTCGTATGTTGTATCAGACTATCAGTAACTCGATCGAGATGTGTACCGATTCTACTGTATCAGACTACTAATTAGTTGAAATCAATGTATGTGTATCTAAATTGTC

>*At*CUC3 |AT1G76420|*Arabidopsis thaliana*

ATGATGCTTGCGGTGGAAGATGTGTTAAGCGAACTCGCCGGAGAAGAAAGGAACGAGAGAGGATTGCCACCTGGCTTCCGGTTTCACCCGACGGACGAAGAGCTCATTACCTTCTACTTAGCTTCCAAAATCTTCCATGGTGGTCTCTCCGGCATTCACATTTCCGAAGTTGATCTCAACCGCTGTGAACCTTGGGAGCTACCAGAAATGGCGAAGATGGGAGAGAGAGAGTGGTACTTTTATAGTCTAAGGGACAGGAAATATCCGACAGGTTTGAGGACTAACAGAGCAACTACTGCTGGATACTGGAAAGCTACCGGCAAAGATAAGGAAGTCTTCTCCGGCGGAGGAGGACAGCTTGTTGGGATGAAGAAGACGTTGGTGTTCTACAAAGGTAGGGCTCCACGTGGCCTCAAGACTAAGTGGGTCATGCATGAGTATCGCCTCGAAAACGACCATTCACACCGCCACACGTGTAAGGAGGAATGGGTGATTTGCAGAGTGTTCAATAAAACAGGAGACAGAAAAAATGTTGGATTAATCCATAACCAAATCAGCTACCTTCATAACCATTCACTCTCAACAACACATCATCATCATCATGAAGCCTTACCTTTGCTTATAGAACCTTCCAACAAAACCCTAACCAACTTCCCATCACTACTCTACGATGATCCACACCAAAACTACAATAATAACAACTTCCTTCATGGATCATCAGGCCACAACATCGACGAGCTCAAAGCCTTAATCAACCCTGTCGTCTCTCAGCTCAACGGTATCATCTTTCCTTCAGGGAACAACAACAACGACGAAGACGACTTCGACTTTAACCTCGGCGTGAAAACAGAGCAGTCTTCGAACGGTAACGAAATTGACGTACGAGATTACTTGGAGAACCCTCTGTTTCAGGAAGCGAGTTATGGTCTGTTGGGTTTTTCGTCTTCTCCTGGACCTCTTCACATGCTACTAGATTCTCCATGTCCTTTAGGATTCCAGCTGTAG

>*Br*CUC3 |Brara.G03438|*Brassica rapa*

ATGATGCTTGCGGTGGAAGATGTGTTGAGTGAGCTCGCCGGAGAAGAAAGAAACGACAGAGGTTTACCACCTGGCTTCCGGTTTCACCCGACGGACGAAGAGCTCATAACTTTCTACTTAGCCTCTAAAGTCTTCCATGGAGGTCTATGTGGCATTCACATTGCCGAAGTTGACCTCAATCGCTGCGAACCCTGGGAACTCCCTGAAATGGCCAAGATGGGAGAGAGAGAGTGGTACTTTTACAGTCTAAGGGATAGAAAATATCCCACAGGGCTAAGGACTAACAGAGCCACTACCGCTGGATACTGGAAGGCTACCGGAAAAGATAAGGAGGTCTTTGCCGGCGGTGGCGGCGGTGGAGGAGCACTTGTCGGAATGAAGAAGACCCTTGTGTTCTACAAGGGTAGGGCTCCACGAGGCCTCAAGACTAAGTGGGTCATGCATGAGTATCGCCTCGAAACTGACCTTTCTCACCGCCACACGTGTAAGGAGGAATGGGTGATTTGCAGAGTGTTTAACAAAACAGGAGACAGAAAGAATGTTGGAATCCATAACCAAATCAGCTACCTCCATAACACTTCACTATCAACAACACATCAACAACATAACCATTATCATCATCTTGAAATCTTGCCTCCTCTTCTTGAACCATCTAAAACCCTAACCAACTTTCCATCGCTACTCTACGATGATACCCACCAAAATTACAATAATAACCTACTCCATGGATCATCAGCCCACAACGTTGACGAGTTCAAAACCCTAATCAACCCAGCCGTGTCTCAGCTCAACGGAGTCATTTTCTCTCCAGAAAACAGCAACTACAACAACGAGGACGACAACAACTTTGGCGTTAAGACAGAGCAATATTCGAACGGTGGCAATAACGATCTTGATGTGCGAGACTACTTAGACAACCCTTTCTGCCAGGAAGCGGGTTACGGTCTGTTGGGTCTTTCATCTTCTCCTGGACCTCTTATGCTATTAGATTCTCCATATGTCCTTTAG

>*Dc*CUC3 |DCAR_013939|*Daucus carota*

ATGTTGGCAATAGAAGAGATATTGTGTGAGCTGAATGGGAGTGAGATGAATGAGCAAGGCATGCCTCCAGGGTTCAGGTTTCACCCAACTGATGAAGAGCTCATCACATTCTACCTTGCTTCCAAGGTCTACAATGGCTCTTTCTGTGGTGTGGACATTGCTGAGGTTGATCTCAACAGATGCGAGCCCTGGGAACTCCCTGACATAGCGAAAATGGGGGAGAGAGAATGGTACTTTTTCAGCTTGAGAGACAGGAAATACCCAACAGGGCTGAGAACAAACCGAGCAACAGGAGCTGGGTACTGGAAAGCTACTGGGAAAGACAGAGAAGTTTACAGCAGCAGCGCCAATGGTGGTGCTGCTACACTTCTTGGGATGAAGAAGACTCTTGTTTTCTACAAAGGCAGAGCTCCCAGAGGTGAAAAGACCAAATGGGTCATGCATGAATATCGCCTTGACGGTGACTTCTCCTGCCGTCACACGTGTAAGGAGGAATGGGTGATTTGCAGAATATTTCAGAAAATCGGAGAGAAAAAGAACGGAGGGCTTCTCCAGGGACAAAGCAGCAGCAGTAGCTACATGCAAGAGGCTTCAAACTCATCCTTGTCCCGTTTGTTTGATCAAAGTCTTAAATCAGGATCAGCTCTATCTTTACAACCTTACCATACACTCCAGTCCCTCCAAAACCAAAACCAAAACCGAAGCCAAGTACTTATAAACAACATCCCGCATGAAGCAGACCTTAAATCCCTCATAACCAACTCATCATCCTCATCTCTGGCAGTGTCACAAGCCAGCCCCTTCCCCGTAAACAATGAACTCAATGGCCTGCAAACATCATGTTCACCGCCAAAAACCAAAATGAAACAAGACCACAATCTTCTCAAAACTCTCCTCCCACACCAGGATTACTATTGTCCCAAGGAGCAAGAAGAAGCTCCTTTTCCTAAAATCTGCAAGACCGAGTCCAACTTTTCGCATTTCCAGTCCCCTCATTTCTCTACTCATCCCCACATTCCTAATTTCCGATTCCCAATTTCTACAACCGCAGAATATGACATGAACCTGGCTCATCAAATCACAAACACCCCCAACTACAAACAAAGCCCATTGCTTTTCAGGAGTTTAGATAGTGATACCAAGAATGTAATGGGGAATAATTGTGGAGTTGGCTTGGGTAGCTGTGGGTTCCCAGCTTATGGTACTGGAGACACTGAAATGTCAACTTCATCATCATCGTGTTCACGAGGACTACCTTTCAGCAGGGCCGGCTTTAAGCAGATGCTGCTGCTGGATCCTCCCACCAAGATGAGCGCAGGAGAATCTTGGCCTTTTCACTTTTAA

>*Eg*CUC3 |Eucgr.F03588|*Eucalyptus grandis*)

ATGCTGGGAGTGGAGGATTTGCTCTGCGAGCTGCGCAGAGAAGAAGGGAACGAGCAAGGGCTACCGCCAGGTTTCAGGTTCCACCCCACTGACGAAGAGCTCATCACCTTCTACTTGGCTTCCAAGGTCTTCAATGGCGCTTTCTCTGGCCTCGACATCGCCGAGGTTGACCTCAACCGATGCGAGCCCTGGGATCTCCCTGAGGTCGCAAAGATGGGGGAGAGAGAGTGGTACTTCTTCAGCCTTCGAGACCGCAAGTACCCGACGGGGCTCCGGACGAACCGGGCCACTGGCGCTGGCTACTGGAAAGCCACGGGAAAGGACAGGGAGGTCCACAGCGCCGCCACCGGGGCGCTCCTCGGCATGAAGAAGACGCTCGTCTTCTACAGAGGCCGAGCTCCCCGCGGCGAGAAGACCAAGTGGGTCATGCACGAGTACCGCCTTGACGGCCACTTCTCCTGCCGTCACACGTGCAAGGATGAATGGGTCATATGCAGGATATTCCACAAGACAATGGAGAAGAAGAGCCTGCTCTTCCAAGGCCAAAATTACATGCTTGAAGTTTGCTCACCCCCTAGCACTGGTTCGTTGCCTCAGCTTCTTGAAACCCCAATTACATCAACGCCACCACCAAATCATCTACTAGAGCACCAATCTCAGGCCAATATCACCACCACCACGCACAAAGACTCGTACAACAACACAGACACCAAAAACATCATCGTCACCCCATCACTAGTCTCGCCATCGTCGATGCTCTTCAAGTCCCTTCTCTCACATCAAGACCTTTTCACAGCTACTAATTTGAAGGAACACCAGCACCACACTGTGCTCAACCAGTGCAAGACAGAGACCAACTTTTCCCATTTCCAGCTAAACGTCGACGATGATAATGATGCCGATGGCTTGCACAGTGTTGTGGACAAGATTCACATTGTCCCATTTCATCAAAATCACCAAAGCCCCGTGCTTTTCGAGATGGACACTCACCCTACAACTACATCTTCTGTTCCAGATAATAATAATGCACCTCATGGCCACACTGTTCATCATGAGATGTCCACGTCAACAATGGCTTTCAACAGGGTCGGGCCGTCCTTTCAGATGCTGGTGGATCCTGCCTTCAGAGAATCCTGGCCGTTGGATCCTTGA

>*Gm*CUC3 |Glyma.06G014900|*Glycine max*

ATGTTGGCAATGGAAGAACTACTGTATGAACTTAGTGATCATGAAAGGAGAAACGAGCAAGGTCTGCCACCGGGTTTCAGGTTTCACCCCACTGATGAAGAACTTGTAACCTTTTACTTGGCTTCAAAGGTCTTTAATGGCACCTTCTCTAACGTCAAGTTTGCTGAGGTTGACCTCAATAGATGTGAGCCTTGGGAACTTCCAGATGTGGCAAAGATGGGGGAGAGAGAGTGGTATCTGTTCAGCTTGAGGGACAGAAAATACCCAACTGGGCTAAGAACAAACAGAGCAACCGGAGCTGGGTACTGGAAAGCCACTGGGAAGGACAAGGAAGTGTACAGTGCATCCAGTGGAACCCTACTTGGAATGAAGAAGACCCTTGTTTTCTACAAAGGCAGGGCCCCTCGTGGTGAAAAGACCAAATGGGTCATGCACGAGTACCGTTTGGTTTTGGATGCTCACTTTTCCCTTCCCCACACTCACCCCTCTAAGGAGGAGTGGGTTATATGCAGGATATTTCATAAATCTGGGGAAAAGAGAAGTCCAGTGCTCCAAGTCCATGGACATTCAGATGCTTCTTCGTCCCCAAGAGAAAGTGCTTTACCTCCATTACTTGCAAGCCCAAGTTGCTTTACATTTGATCCAGAATCTCAATCCCAAAGTTCATCTCATTCTCAGCGAGACTTCCAAAGCCCGGTTCTGATTCACCACCAAGACCAAAATGGCCACTCTCATAACCCTCGTCTTTTCCCATTAGAAATCACCAACGCCAGAAACCACCCATCATCATTTTCGGACCTATTCTTCAAGCCCCTTCAGCAGAATTGCACCCTGAAGACCAACGAACAAACTATTCTTCCAAAAGTAACCAAAACGGAGGACGCTACATTCTATGATCAATATCATCAATTACTAGATGACCATAATAACATGCGCTGGGTGAACAAGTTGAATCAAAATCCAAGCAATTTACTCAACACTTTCCCTTTTGAGGTGGATGCTGGCTTGATGGCATTCTCAGGAGCTGCAAATGCTCAAGTTAAGGACATATCCACTTCAACACCCTTTAATAGGGTAGGCTTGCAGCAGACGCTAGATTCTTGGCCTCTGGCCCAGCATGTTTGA

>*Gr*CUC3 |Gorai.002G113300|*Gossypium raimondi*

ATGTTCCTCCATTCATTCCAGTCACAACAACTAGCCTTTACTCTCAACAAGATGTTAGCAGTGGAGGAAGTTTTAAGTGAACTTGGCGGGGAAGAAGTGAACGAGCAAGGGTTGCCGCCGGGGTTTAGGTTTCACCCCACTGATGAAGAACTCATAACATTTTATTTGGCTTCAAAGGTATTCAATGGAAGCTTCTGTGGAGTGGACATTGCTGAGGTTGACCTTAACCGATGTGAACCTTGGGAGCTTCCAGATGTGGCGAAAATGGGGGCAAGGGAGTGGTACTTCTTCAGCCTGAGGGACAGGAAATACCCGACGGGAGTGAGAACAAATAGAGCTACCGGAGCTGGGTACTGGAAAGCCACCGGAAAAGATAGGGAAGTGTACAGTGCCTCCACTGGAGCTTTACTTGGCATGAAAAAAACCCTTGTTTTCTACAATGGCAGAGCACCCCGTGGAGTGAAGACCATGTGGGTCATGCATGAGTACCGTTTAGACGGTGACTTCTCCTGCCGCCACACGTGCAAGGAAGAATGGGTGATTTGCAGAATAATACACAAAACAGGTGAGAAGAAAAATGGGGTAGCTGCTGCGCAAGGGCTAGGCTATATCTTGGAACTTTCTTCATTATCTTCAACAACAAAAACAACAAATTGCCTCCGTCCACTGCTTGAAACCCCAACTCCTTTGTTAGAATCTCAAACCCAAATTTCAATGCAGGCTGCCCATAACTCTTTTCTGGAAAATGACCTGAAAAGCTTAATAAACCCGGTTGTGTATCCAGCTAATGGGTTCCAACCCTCCTTTACAGCCACTCCCACCACCTTTAGTAGCACACCCGACAAGAACGCCAGCAGCAACTCATCAGCAGCCGCATCGATGCTCTTCAAGTCCCTCCTCTCACATCAAGAATGCGTTTTGAGGGAACAAGCAGCTGCTACTATTCCCAAACAGTGCAAGACAGAAGCTAACTTTTCCAATTTCCAACTGCCTGATTCCACCTTGAGTTGGACGGAGAAGATGCATCCCAACCCTTGTCAAGATCCCATGTTTTTCGACATGGATTATAATAATAGTGTGTTGGGGTTCGCTGAACTCTGA

>*Cs*CUC3 |orange1.1g016283m|*Citrus sinensis*

ATGGAAGACGTTTTGAGTGAACTGAATGGAGATGAAGTGAATGAGCAAGGGTTGCCACCAGGGTTTAGATTTCACCCAACTGATGAAGAGCTCATCACATTTTATTTAGCCTCAAAGGTCTTTAATGGCACCTTCTGTGGTGTTGAAATTGCTGAAGTTGACCTCAACAGATGTGAACCTTGGGAGCTTCCTGATGTAGCAAAAATGGGGGAGAAAGAGTGGTACTTCTTCAGCTTAAGAGACAGAAAGTATCCTACTGGATTAAGGACAAACAGAGCAACAGGAGCTGGTTACTGGAAAGCTACAGGCAAAGATAGAGAAGTGTGCAGTGGCTCAAGTGGAGCCTTACTTGGCATGAAAAAAACCCTTGTTTTTTACAAAGGCAGAGCTCCTCGTGGCGAAAAGACCAAATGGGTCATGCATGAATACCGCCTCGACGGTGACTTCTCATACCGCCACACGTGTAAGGAGGAATGGGTGATATGCAGGATATTTAACAAAGCAGTAGTGAGTGGGGAGAAGAAAAATGGATTGCTTCTCCAAGGACAACACTATTTGTTTGAGGCAGCTGCAACAGCTGGTGCTTGCTTGCCTGCTTTGCTTGATGCCCCAGGGCCAGCAACGACAACATTACTGTTGGAATGTCAGTCTCAAAACCACAACCCAATCTTGGAAAATCTTCCGAACCATTTCGTGAATCAGCAACAAGACAACCATCATCATCACCTATTCCCAGTAAATGGCTTGTTTGAGACCTCCGCAGTAACAAACAAACACATTTTGATTAACAACATCACTGAAAACATTGGCAACAACACATCACCATCCATGCTTTTCAAGGCACTCCTCTCACATCAAGATTTCAGCTGCTGCAATGAATTAGCCCCTAGTCCCAAACACTGCAAGACAGAAGCAAACTTTTCCCATATCCAGCTGCCTCCTGCTACTGCTGCTGATGACAATAGTAATGATAACTGGAGCAATTGCTACTGGATGGACAGCAAAATTCAACCAAACCCATATTCAAATCCCTTGTTTTCTGAGTTCGATTGTAGCTTTCCTGGACTCACACAACCCTCTGCCTTTGCCGCTACTGCTGTCAATGACATGTCCACTTCAATTGCTTTCAACAGAACCGGCTTTCAAGTCGTCGAAAAATCTTGGCCATTGGGTGCTTAA

>*Pv*CUC3 |Phvul.009G008000|*Phaseolus vulgaris*

ATGCTGGCAATGGAAGACCTACTGTGTGAACTTAGTGATCATGAAAAGAGAAACGAGCAAGGTCTGCCACCGGGTTTCAGGTTTCACCCCACTGATGAAGAACTTATAACCTTTTACTTGGCTTCAAAAGTCTTCAATGGGAGCTTCACTAGTGTCAAGTTTGCTGAGGTTGATCTCAATAGATGTGAGCCGTGGGAACTTCCAGATGTGGCAAAGATGGGGGAGAGAGAGTGGTATCTGTTCAGCTTGAGAGACAGAAAATATCCAACTGGACTTAGAACAAACAGGGCCACCGGAGCTGGGTACTGGAAAGCCACTGGGAAGGACAAGGAAGTGTTCAGTGCATCAAGTGGAACCCTACTTGGGATGAAGAAGACCCTTGTTTTCTACAAAGGAAGGGCCCCTCGTGGTGAAAAGACCAAATGGGTCATGCATGAGTACCGTTTGGACGGTGACTTTTCCCTTCCCCACCCTCACCCTCATCACATTTCTAAGGAGGAGTGGGTGATATGCAGGATATTTCATAAATCCGGGGAAAAGAGGACTCCACTGGTCCAAGTCCAAGGACATTCAGATGCTTCTTCGTCTCCAACAAAAAATTCTTTACCTCCTTTACTTGCAAGCCCAACTTGCTTTACATTGGAACTAGAATGTCAATCCCAACAAAGCCCGGTTCTCATTCACCACCACCAAGACCAAAACCACCTCTCTCACTCTCACCCTTATCTTTTCCCATTGCATGCATCACCACAACTCACCAACGCCAGAAACCATCCTTCATTTTCTGACCTGTTCTTTAAGCCCCTGCACAACTCACAACAAAACTGCATCTTCAAGGCCAAGGAAAAAACAGCTCCAAAAGTAGTCAAAACGGAGGAGGCTACAGCATTCTATCAGTACCATTTACTAGGTGACGCCAATAACTTGCGGGTGAACCAAAATCCGAGCAATTTCCCAAACCCTTTCCCTGATGTTGAGGTGGATGCTGGGCTGATGGCATTCTCAGGAGGTCCAAATGCTGAAGTTAGGGACATGTCCACCTCAACTGCCTTTAATAGGGTAGGGTTGCAGCAGGTGATAGATGCTGCTCATATCGGAATAGATTCTTGGCCTCTGCCCCAGCATGTTTAA

>*Sl*CUC3 |Solyc12g036480|*Solanum lycopersicum*

ATGGATGAAAATCTTCCTCCAGGGTTCAGGTTTCATCCAACAGATGAAGAACTCATTACTTGTTATTTAAATAACAAAATTTCTGATTTCAATTTCACTACTAGAGCTATTGCTGATGTTGATCTCAATAAGTCTGAGCCTTGGGACCTCCCTGCAAAAGCGTCAATGGGAGAAAAAGAATGGTATTTCTTCAGTCTAAAAGATCGAAAGTACCCAACAGGGCTTCGAACAAATAGAGCTACAGAAGCAGGCTACTGGAAAACAACAGGGAAAGATAAAGAGATATATCGTGGTGGAACGGGAGTTCTTGTTGGGATGAAGAAAACCCTAGTTTTCTATAGAGGAAGAGCTCCTAAGGGTGAAAAAACCAATTGGGTTATGCATGAATATAGAATTGAAACAACATTTGGTTACAAACCTTCTAAGGAGGAGTGGGTAGTGTGCAGGGTGTTCCAAAAGAGTTCAACTGTGAAAAAGCCACAACCAACATCATCTTCTCCATTATCCCTAGAGTCACCTTGTGACACTAATTACACAATAACAAATGAGCTTGGTGATATTGAGCTACCATTTAATTTCAACTACCTTACCACTACTCCATCAACCGCGATCAATAATATTTCCTTGCATAATTACAACAACGATAACATAAACTTGGCTGCTGCAACAAGAGAAGCAAACAGTCATCCATTACTACCTTGGTCTTCCAACTTGTTAAGCTCAAATCTTTCATCAGTAAATTCATTACTTTTTAGGGCATTGCAATTAAAGAGTTATTCGCCAAGAGAACAAGCTACAACGACTCATGACTACGCGTTTATGCTCCCACAGGAGAATATTATTACAACGCAATTTGGAAATGATTTTGCTGTGAATAATATTGGGGCACCGTCTTCATCTACGGTGTTAGATAATTCTGTACAGCAGCAACAACAACAGCAACAAGAACAATCGTACAAATTGGACTCCAATATTTGGTGA

>*Ss*CUC3 |Sspon.06G0001780-1A|*Saccharum spontaneum*

ATGCACCACCACCAGGCCATGAGCGACGCGCTGTGGGACCTGCTCGGGGAGGAGATGGCGGCGGCGGGCGGCGAGCACGGCCTTCCCCCGGGGTTCCGGTTCCACCCCACCGACGAGGAGCTGGTCACCTTCTACCTGGCCGCCAAGGTGTTCAACGGCGCCTGCTGCGGCATCGACATCGCCGAGGTGGACCTCAACCGGTGCGAGCCGTGGGAGCTCCCCGACGCGGCGCGCATGGGGGAGCGCGAGTGGTACTTCTTCAGCCTCCGCGACCGCAAGTACCCCACGGGCCTCCGCACCAACCGCGCCACCGGCGCCGGCTACTGGAAGGCCACCGGCAAGGACCGCGAGGTGCTCAACGCCGCCACCGGCGCGCTCCTCGGCATGAAGAAGACGCTCGTCTTCTACAAGGGCCGTGCGCCGCGCGGCGAGAAGACCAAGTGGGTCCTCCACGAGTACCGCCTCGACGGCGACTTCGCCGCCGCTCGCCGCCCCTGCAAGGAGGAATGGGTGATCTGCAGGATACTGCACAAAGCAGGCGACCAGTACAGCAAGCTGATGATGGTGAAGAGCCCCTACTACCTCCCCATGGCAATGGACCCCTCCAGCTTCTGCTTCCAGCAGGACCCCACCGCGCCTCCCCTCCAAAACCCTAGCGGCTGCATCCCCTTCCAGCACGGCCACCCCAGCATGCAGCCGCCTCCACTGCCGCCGAGCAACCATGGCAAGGTCGTCTTCACCGGAGCAGCGGCGCCCTGCATGCAGCAAGAGCCGGCAAACGGCAGCAACAGCGCCGTGCTGCCGATGCCGCCGTTACCTCACTTCACCCCCATCGTCGCCGGCAAGCCGGCCCCGGCGCCGCCGCCCCAGGTCGGGGTCAACGCCGGTCCACAGGAGCCACCGCCACCGCCACCTACCTGGCTGGAGGCCTACCTGCAGCACGGTGGTGGGTTCCTTTATGAGATGGGCCCAGCTGCAGCGCCCAGGGGCGCATGA

>*Ac*CUC3 |Aco000744|*Ananas comosus*

ATGCATACCAATATGGGGGAGTTGGTGTGGGAGTTGTTTGGAGAGGAAACTTACAATGAGCAGGGATTACCTCCTGGATTTAGGTTCCATCCGACTGATGAGGAGCTTGTGACCTTCTACTTGGCATCCAAGGTGTTCAATGGGGGCTTATGTGGGGTGGATATAGTCGAGGTTGACCTTAATAGATGTGAGCCATGGGAGCTCCCAGACGCGGCAAAGATGGGAGAGAGGGAGTGGTACTTCTACAGTCTCCGCGACCGGAAGTATCCGACGGGGCTGAGAACGAACAGGGCGACGGGGGCCGGCTATTGGAAGGCCACCGGAAAGGACAGGGAGGTACACGGCGCCGCCAATGGGGCCCTTGTCGGCATGAAGAAGACGCTCGTCTTCTACAAGGGGAGGGCGCCGCGCGGCGAGAAGACCAAGTGGGTCCTTCACGAGTACCGTCTCGAAGGTGACTACGCCTGCCGCCACCGCTGCAAGGAAGAATGGGTGATATGCAGGATATTTCACAAGACAGGAGGAGACAAGAAGAACCAATACTACCCAAACCCCTCATATACAATAAACCCATCCTCTTCCACTCCCAGTACTTGTATCCTCCCATTCCTAGACCCCCAAACCCTAGAAACCCCTCTCCAAACCCTCCACAACCACCACCAACCCTATTTCCATCTAAACCAAGAACCCATTAATCCCCTCTTCCCCCTCCCTCCTCTCCCTTCCTTTGCTTGTTCCTCCACCTTCCTCCCATCCTTCCCTAAAAGCCCTCCAAAGGAAGAGGACACAAATGCACTATTAAACCCTAATGAGGAGGCCATGTTCCCTGCTAATTGGCTCGAAACGTACATACAAAACCCCTTCGTTTATGAGATGGGCTTTTCACTTCCGGGCCCTGGGGCACCAGTTTATGACGTGCCCCTCCTGGGCTACACTGCCACAGGAGAATCTGGACCGTTG

>*Os*CUC3 |LOC_Os08g40030|*Oryza sativa*

ATGGGGGACGCGCTGTGGGAGATGCTGGGGGAGGAGATGGCGGCGGCGGCGGCGGCGGCCGGCGAGCACGGGCTGCCGCCGGGGTTCAGGTTCCACCCCACCGACGAGGAGCTCGTCACCTTCTACCTCGCCGCCAAGGTGTTCAACGGCGCGTGCTGCGGCGGCGTGGACATCGCGGAGGTGGACCTGAACCGGTGCGAGCCGTGGGAGCTGCCGGAGGCGGCGAGGATGGGGGAGAAGGAGTGGTACTTCTTCAGCCTCCGCGACCGCAAGTACCCGACGGGGCTGCGCACCAACCGCGCCACCGGCGCCGGCTACTGGAAGGCCACCGGCAAGGACAGGGAGGTGGTCGCCGCCGCCGCCGCCGGCGGCGCGCTCATCGGCATGAAGAAGACGCTCGTCTTCTACAAGGGCCGCGCCCCGCGCGGCGAGAAGACCAAGTGGGTCCTCCACGAGTACCGCCTCGACGGCGACTTCGCCGCCGCTCGCCGCTCCACCAAGGAGGAATGGGTGATCTGCAGGATCTTTCACAAGGTAGGAGATCAGTACAGCAAGCTGATGATGATGAAGAGCCCAGCCAGCTACTACCTCCCAGTGAGCCACCACCACCCCAGCAGCATCTTCCATGACCTTCCTCCGGTCCCATTCCCAAACCCTAGCCTCGTCCCCTTCCACCATGATCTCCCCACAAGCTTCCATCCTCCATTGCTGCAGCACAGCCATGCGAACAGCAAGAACAGCAGCAGCAACAATGGCGGCTTCGTCTTCCCCAATGAGCCAAACACCACAAACAGCAGCGATAACCACATTTCTTGCAATGGCGCCATGGCTGCTGCTGCTGCTGCTGCTTTTCCTTCCTTCAGCTGTGCTAGTACTGTCACTGGCAAGGGGGGCCCACCGGCGCAGCTCGGAGTCAACGCCGGTCAACAGGAGCCACCGCCACCTACCTGGATGGACGCTTACCTGCAGCACAGTGGATTCATTTATGAGATGGGCCCACCTGCAGTGCCCAGGGGCGCATGA

>*Bd*CUC3 |Bradi3g40085|*Brachypodium distachyon*

ATGCACCAGCACCAGCACCCTGCGGCGGCGGCCATGGGCGCGGAAGCCCTCTGGGACATGCTCAGCGAAGACATGGCAGCCGCAGCGGCCGCCGCAGCCGAGCACGGCCTCCCCCCGGGCTTCCGCTTCCACCCCACCGACGAGGAGCTCATCACCTTCTACCTCGCCCCCAAAGCCTTCAACAGCAGCAACGACAACGACAGCAACTTCTCGGCCGTGGACTTCATCGCGGAGGTGGACCTGAACCGGTGCGAGCCGTGGGCGCTGCCGGAGTCGGCCAGGATGGGCGGGGAGCGGGAGTGGTACTTCTTCAGCCTCCGCGACCGCAAGTACCCCACGGGGCTCCGGACCAACAGGGCCACGGGCGCCGGCTACTGGAAGGCCACGGGGAAGGACAGGGAGGTCGTGTGCGCCGCCACGGGGGCGCTCATCGGGATGAAGAAGACGCTCGTCTTCTACGAGGGCCGAGCTCCCAGGGGACACAAGTCCAAGTGGGTGCTCCATGAGTACCGCCTGGACGGCGACTTCGCCGCCGACCGCCGCTCCTGCAAGGAGGAATGGGTGGTGTGCAGGATCCTCCACAAGACAGTAGACCAGTACAGCAGCAAGATGATGGAGATGAGGATGATGAGCCCCTACCACCACTGCTACCACCCCATGAGCCACCACCACCACCCAAGCTTCGTCTTCCAGGACGCGCCTCCCGTCCCCTTCCCAAACCCTAGCGGCCAGCTCCCCGTCCCCTTCCTCCACCACCACCATGACCTGATCCCAAACCTTCAGCAGCCCTCGCCATTAACAACGCAGCACCACCACCAGCCCCAGGCGGCCGATAAGAACTCAAGCAGCAACAATGGCGGCTTCCCAGTCCCAGCCGCAGCAGCGGCTTGCATCCAAGATCAGCAGCCAGACAACAACACGGCGCCATACTTCCCTTTCCCTTCCTTGGCCTCCGCCGTCACCGTCGCTGCCAAGGCGGGCCCACTGCCCGGAGTCAACGCCGCCGGTCCGCAGGAGCTGCTGCCGCCGACGTGGCCGCTGGACAACTTCCTGCAGCATGGCATTGCCACCTACCTCTACGAGACGGGCCCACCCGCAGGTGCCCCCAGGGACGCGTGA

>*Zm*CUC3 |GRMZM2G430522_P01|*Zea mays*

ATGGTCTTATGGAGGACAGGAGGAGCATGGTGGTGCTACCTAGCAAGAGCTCCCCACTATAAAGCGCCCCCACACACCATACCACAGCTCAGAGCTTCTTCTCATCATCTGGTAGAAAGAAAGAGTGAGAGTGAGGTTGGCAAGGGTATAGGGTTCTTGGTCGATCAAATACCTTTCCCCTCTTGGATTCTCATCTTCCTGCTTCGTTCTCACCAGATCGATCTCACCACGTGCCTGCGTAGCAAGCCACTCTGTATGCACCATCACCACCAGGACCAGGCCATGGGCGACGCGCTGTGGGACCTGCTCGGGGAGGAGATGGCAGCGGCGGGCGGGGAGCACGGGCTGCCCCCGGGGTTTCGGTTCCACCCCACCGACGAGGAGCTGGTCACCTTCTACCTCGCCGCCAAGGTGTTCAACGGCGCCTGCTGCGGCATCGACATCGCCGAGGTGGACCTCAACCGGTGCGAGCCGTGGGAGCTCCCCGACGCGGCGCGCATGGGGGAGCGCGAGTGGTACTTCTTCAGCCTCCGCGACCGCAAGTACCCCACGGGCCTCCGTACCAACCGCGCCACCGGCGCCGGATACTGGAAGGCCACCGGGAAGGACCGCGAGGTGCTCAACGCCGCCACCGGCGCGCTCCTCGGCATGAAGAAGACGCTCGTCTTCTACAAGGGCCGGGCGCCGCGCGGCGAGAAGACCAAGTGGGTCCTGCACGAGTACCGCCTCGACGGCGACTTCGCCGCCGCTCGCCGCCCCTGCAAGGAGGAATGGGTCATCTGCAGGATACTGCACAAGGCAGGCGACCAGTATAGCAAGCTGATGATGGTGAAGAGCCCCTACTACCTGCCCATGGCGATGGACCCTTCCAGCTTCTGCTTCCAGGAGGACCCAACCGGGCATCCCCTCCCGAACCCTAGCGGCTGCACCCCCTTCCACCACGGCCACCCCCACCATAGCATGCAGCCGCCGCCTCCATTGCCGCCGAGCAACCATGCTGGCAAGGCCGTCTTCACCGGAGCAGCAGCAGCCTGCTGCATGCAACAAGAGCCGGCAGACGGCAGCAACAGCGCCGTGCTGCCCATGCCGCCGTTCCCTCCCTTCACCCCCATCGTCGCCGGCAAGCCGGCGGCCCCGGCGCCGCCGCCCCAGGTTGTCAACGCCGGTCCACAGGAGCCACCGCCACCTACCTGGCTGGAGGCCTACCTGCAGCACACTGGTGGGATCCTTTATGAGATGGGTCCAACTGCAGCGCCCAGGGGCGCGTGA

>*Hv*CUC3 |MLOC_13932|*Hordeum vulgare*

ATGCACCAGCACCAACCAGCGGCGGCCATGGGCGACGCTCTGTGGGAACTGATCGGGGAGGAGATGGCGGCGGCGGAGGCGGCTGCCGGGGAGCACGGCCTGCCCCCAGGCTTCCGCTTCCACCCCACCGACGAGGAGCTTGTGACCTTCTACCTCGCCGCCAAGGTCTTCAACGGCACGTGCTGCGGCGGCGTGGACATCGCAGAGGTGGACCTGAACCGGTGCGAGCCGTGGGACCTCCCGGAGGCGGCGAGGATGGGGGAGCGGGAGTGGTACTTCTTCAGCCTCCGCGACCGCAAGTACCCCACGGGCCTCCGCACCAACCGCGCCACCGGCGCCGGCTACTGGAAGGCCACCGGCAAGGACCGCGAGGTGCTCAATGCCGCCACGGGCTCCCTCCTCGGCATGAAGAAGACGCTGGTCTTCTACAGGGGCCGCGCCCCCCGCGGCGAGAAGACCAAGTGGGTCCTCCACGAGTACCGCCTCGACGGCGACTTCGGCGCCGTCCGCCGCTCATGCAAGGAGGAATGGGTGGTGTGCAGGATCTTCCACAAGGCGGTAGACCAGTACAGTAAGATGATGGAGATGAGAAACCCCTACTGCTACTTCCCCATGACCCCCCACCACCCCAGCTTCTTCCAGGACGCACCTCCTGTCCCCTTCCCAAACCCTAGTCAGCTCATCCCCTTCCACCATGACCTCCCACACCTGCAGTCCTCCCCATTAATACAGGGCCAGGCCAAGAACACAAGCAACAACAGCAATGGCGGCTTCCCAGCAGCAGCTAGCATCCAAGAGCAGCCAAACAGCAGCTGCAACCCAGCATATTTCCCTTTCCCTTCCTTCGCGTCCATCGTCAATGGCAAGGCAGGCCCACCGGCGCAGCCCGGGGTCAACGGCGGTCCACAGGAGCCACCGCCGACCTGGCTGGACGCTTGCCTGCAGCACAGCGCCTTCATGTACGAGATGGGCCCACCTGCAGCCACGAGGGGCGCATGA

**C) Protein sequences**

>*At*CUC1|AT3G15170|*Arabidopsis thaliana*

MDVDVFNGWGRPRFEDESLMPPGFRFHPTDEELITYYLLKKVLDSNFSCAAISQVDLNKSEPWELPEKAKMGEKEWYFFTLRDRKYPTGLRTNRATEAGYWKATGKDREIKSSKTKSLLGMKKTLVFYKGRAPKGEKSCWVMHEYRLDGKFSYHYISSSAKDEWVLCKVCLKSGVVSRETNLISSSSSSAVTGEFSSAGSAIAPIINTFATEHVSCFSNNSAAHTDASFHTFLPAPPPSLPPRQPRHVGDGVAFGQFLDLGSSGQIDFDAAAAAFFPNLPSLPPTVLPPPPSFAMYGGGSPAVSVWPFTL

>*Br*CUC1|Brara.C03560|*Brassica rapa*

MDMDVFNGWERSRYEDETVMPPGFRFHPTDEELITYYLLKKVLDSSFSCAAISQVNLNKSEPWELPEKAKMGEKEWYFFTLRDRKYPTGLRTNRATEAGYWKATGKDREIKSSKTNSLLGMKKTLVFYKGRAPKGEKSCWVMHEYRLDGKFSYHYITSSAKDEWVLSKVCLKSSVVSRETKLISSSGGVNCSSSSSAAGSLIAPMIDAYATEHVSCFSNTSAAHADASFPPAYLPAPPPPSLPRQPRCFGDDVAFGQFMDVGASGQFSIDAAFLPNLPSLPPTVFTTPSQPFGMYGGGSAVSSWPFAL

>*Dc*CUC1|DCAR_020996|*Daucus carota*

MDIFYHNLQSNADAQLPPGFRFHPTDEELITYYLLNKVLDHNFTCRAIAQVDLNKCEPWHLPERAKMGEKDWYFYSLRDRKYPTGLRTNRATEAGYWKATGKDREIYNSKTSSLVGMKKTLVFYRGRAPKGEKTNWVMHEYRLDGKLAYHYLSTNSKDEWVISRLMKKSGTAGPGDKTPTYGMYSEMSSSSSASLPPLLDSTPFTAATTDHHRVIDYSYLEKEHVSCFSRSTAAPGGFNYHTTLFDCGLPPPLMVDHTSSSTSSSSQFHENNTNGGEKLHLPSFFFPSITPSPIHGGVGSIYASDTGNYSVLEAQKPGLTELDCIWRGSFN

>*Sl*CUC1|Solyc06g069710|*Solanum lycopersicum*

MENYSGVVKDDDQMELPPGFRFHPTDEELITHYLSNKVVDTNFVAIAIGDVDLNKVEPWDLPWKAKMGEKEWYFFCVRDKKY

PTGLRTNRATAAGYWKATGKDREIFRGKSLVGMKKTLVFYKGRAPKGEKTNWVIHEFRLEGKLSLQNLPKTAKNEWVICRVFQKSSGGKKIHISGLLKLNSNENEMGNSFLPPLTDSATATASKSSHVHCFSNFLTAQNNCFPLLSNPMDSYPTTSLVPNTFSCNQIAPFTTTNNPASFGVQDPSILLRTSLDSYGLNFKKEDIFNVPQETGVISTDMNTDITSVVSNLEMKRRFLEDQVPSAGMVGLQGLDCLWSC

>*Eg*CUC1|Eucgr.F01170|*Eucalyptus grandis*

MENYHQYSNHHLVNGDGHLPPGFRFHPTDEELITYYLLKKVLDSSFTGRAIAEVDLNKCEPWELPEKAKMGEKEWYFFSLRDRKYPTGLRTNRATEAGYWKATGKDREIYSGKTGSLVGMKKTLVFYRGRAPKGEKSNWVMHEFRLDGKFAYPFLSRSSKDEWVISRVFQKSSNSCGAAAPCGGKKTRMPPHMNLYPEIGSPSVSLPPLLDSSPYTSTTAGFIDRVPISYDSSIPKEHVSCFSTAAAASLATHNFGSNPSFQLAPPTAPLINATDPMTRFSRSIGVSAFPSLRSLQENLQLPYFFSGNQHLSGGINDLVSSTSSSLGNWTAPDDQKAVDLGGRMGMGSSELDCMWNF

>*Cs*CUC1|orange1.1g017827m|*Citrus sinensis*

MENVSAVGKEDDQMDLPPGFRFHPTDEELITHYLYKKVLDVCFSCRAIGDVDLNKNEPWELPWKAKMGEKEWYFFCMRDRKYPTGLRTNRATVSGYWKATGKDKEIYRGKSLVGMKKTLVFYRGRAPKGEKSSWVMHEYRLDGKFSVHSLPKTAKNEWVLCRVFQKGSGGKRTHISGLAGLGSFGNELGPPGLPPLMDSSPDNGSKTIKSVADSAYVSCFSNSIDLQRNQKTTTTIENFFNNPPPISVSSNCPDVFPRIPLSSNSFYSPLSVPVPSHAQFPGSVFMQDHSILRALIENQGSNMSQSFKTEREMISVSQDTGLTADMNPEISSVVSNLEMVKRPFNDHDAPSTSAGPVDFDCFWNY

>*Gr*CUC1|Gorai.007G323900|*Gossypium Raimondi*

MDSYYHFDNGDTHLPPGFRFHPTDEELITYYLLKKVLDSGFAGRAIAEVDLNKSEPWELPGKAKMGEKEWYFFSLRDRKYPT

GLRTNRATEAGYWKATGKDREIYSSKTCALVGMKKTLVFYRGRAPKGVKSNWVMHEYRLEGKFAYHYLSRSSKDEWVISRVFQKSGSANGATSSTGRGAKKTRMNASIAVYQEPSSPSSISLPPLLDPTATAFATTDHDSFSYDNYVQSEHVSCFSTVTAATAASATATTTTAPSAFHPGFDKAFPPPPQMINTTFDPLAKYSRNVGASVFPTLRSLEENLQLPLFFSQPTIEAPTLHGGSSVNWGAFSEEINDGSVGGNKISIGPTELDCMWTY

>*Gm*CUC1|Glyma.12G226500|*Glycine max*

MDHTEAHLPPGFRFHPTDEELITYYLLKKVLDSTFTGRAIAEVDLNKSEPWELPEKAKMGEKEWYFFSLRDRKYPTGLRTNRATEAGYWKATGKDREIYSSKTCSLVGMKKTLVFYRGRAPKGEKSNWVMHEYRLEGKFAYHYLSRNSEDEWVISRVFRKSNTTPITNGGSTMSASTNSKKTRINNTTSLIHEPGSPSSVFLPPLLDSSPYTNTTTNTFTDHHNSSYDSATKKEHVSCFSTIAAATAVVSPNNNFNNASFDLPPSQPLATDPFARFQRNVGLSAFPSLRSLQDNLQLPFFFSTAAAPPFSGGGSGDFLSWPVPEDGVSNMPLGVSELDCMWGY

>*Pv*CUC1|Phvul.011G160400|*Phaseolus vulgaris*

MDSGYYNQRHHPHLDNNNEQHLPPGFRFHPTDEELITYYLLKKVLDSSFTGRAIVEVDLNKCEPWELPEKAKMGEKEWYFYS

LRDRKYPTGLRTNRATEAGYWKATGKDREIYSSKTCSLVGMKKTLVFYRGRAPKGEKSNWVMHEYRLEGKFAYHYLSRSSKDEWVISRVFQKNNTGGASTVSAAVATGGSKKTRISTSNTSSNMSLCPEPGSPSSIYLPPLLESSPYAASSSAVATFNDRENYSFESAAAAAAANQREHVSCFSTLSTDASAFNQLAPQPEPPLDPFSRFHRNNVGLSAFPCLRSLHDNLNLPFFFPPMVHGGTDVANFSAVANFPAPEDPRVVDGSSGMSIVPSELDCMWGY

>*Ss*CUC2|Sspon.07G0020380-1A|*Saccharum spontaneum*

MERFGVLGTRLGLDGVVGGGGGELPPGFRFHPTDEELITYYLLRKAVDGSFCGRAIAEIDLNKCEPWELPDKAKMGEREWYFYSLRDRKYPTGLRTNRATLAGYWKATGKDREIRSARSGALVGMKKTLVFYRGRAPKGQKTHWVMHEYRLEGTYAYHFLHSSTRDEWVIARVFQKPGEVPPARKHHRLGGLSSAGGGESCFSDSTSASIGGGGGGASASSAPRPLPLTVTDASSLSLFASAAAANAADGDSSSYCGGAANNANNGNNLVTGRELVPCFSTSTTTGAGGLDAAALGIGQPYNAAVPLPLAFEPPPPTPAFFPNLRSSLQLQVQQDNNLELPLFLSAAGGLSAATLGMGSMGGGALHHWPLAGMEVKVEGRSAPPQMAVGPGQLDGAFGWGY

>*Ac*CUC2|Aco020094|*Ananas comosus*

MESYAAQHHRFDSGDAQLPPGFRFHPTDEELITYYLLKKVLDGGFTGRAIAEIDLNKCEPWELPEKAKMGEKEWYFFSLRDRKYPTGLRTNRATEAGYWKATGKDREIFSSRTGSLVGMKKTLVFYRGRAPKGEKSNWVMHEYRLDGKFAYHFLSRSSKDEWVVSRVFQKIGGGKKTRLGLAGPSNSDAAGGGVGSQSSSSLPLLLDSSPFAGAASSFASADRESCSYESTDREPVPCFSTTASHLLGNEATPPPLFGRVGTTAAATAATNVNVGLAFPCLRSLQENLQLPFFLSGLAPPLPPLPGSGRPRAEGGAGQQGPPPDDDAGGVHRAGLPLDVLACLTTDLVLPTHAICLTYDYD

>*Os*CUC2|LOC_Os06g23650|*Oryza sativa*

MERCSVLGLGGGGGGGGRLDGELPPGFRFHPTDEELITYYLLRKVVDGSFNGRAIAEIDLNKCEPWELPEKAKMGEKEWYFYSLRDRKYPTGLRTNRATGAGYWKATGKDREIRSARTGALVGMKKTLVFYRGRAPKGQKTQWVMHEYRLDGTYAYHFLSSSTRDEWVIARIFTKPGVFPVVRKGRLGISGGGGDTSCFSDSTSASVGGGGGTSASSALRAPLAEASLFAAAAAPAVDGADSSNYGGGGGAGSATATANLVTGLELVPCFSTTAHMDASFGTGQYNPAPLAVEPPPPPPAFFPSLRSLQENLQLPLFLSGGMQAGVSSQPLSGGGAFHWQSGMDVKVEGAVGRAPPQMAVGPGQLDGAFAWGF

>*Bd*CUC2|Bradi1g41712 |*Brachypodium distachyon*

MERYGLLGTREEELPPGFRFHPTDEELISYYLARKVADVNFSGARAIAEIDLNKCEPWELPDKAKMGEKEWYFYSLRDRKYPTGLRTNRATGAGYWKATGKDREIRSARTGALVGMKKTLVFYRGRAPKGAKTQWVMHEFRLDGNCAYHFFSNNNATRDEWVIAKIFVKPGALPAARNKLARFGLQGSTGGADTSCFSDSTTSVSIGCGGGGGDTTTNTSSLFAAAADGESSSCGGGNNNNCGRELVPCFSTGAHMDATLLGIGQYDPAPLAMEQPPALYQLSAARSVQDNLLFLSGGGLQSGLVSPLGVGGGAFQYWPTSSGYDMKPPQMAVGPGQLDGSFGWGF

>*Hv*CUC2|MLOC_65286|*Hordeum vulgare*

MERYGSLGMRLDGIGGGGGELPPGFRFHPTDEELITYYLLRKVVDCGFSGARAIAEIDLNKCEPWELQDKACKATAEKEWYFYSLRDRKYPTGLRTNRATGAGYWKATGKDREIRSARNGALVGMKKTLVFYRGRAPKGQKTQWVMHEFRLEGVYAYHFLPNNTTRDEWVIAKIFVKPGAAPPSRKARYGLSSAGDTSCFSDSTSVSIGGGGGASASSAPRQQLPDTSSLLAAAHAAADGESSSYGATGNNNNAAGNCRELVPCFSTAQMDATLLGIGQYEPASLAVEQPLAFFQGPRLHQAADNLSLPMFLPGGLQSGVSPLGMGGGAFQHWPSSGYEVKLEGSRAPPQMAVGPGQLDGAYGWGF

>*Zm*CUC2|GRMZM2G139700_P01|*Zea mays*

MERLGVGVGVGELPPGFRFHPTDEELITYYLLCKAVDGGFCGGRAIAEIDLNKCEPWELPDKAKMGEKEWYFYCLRDRKYPTGLRTNRATAAGYWKATGKDREVRSGRSGALVGMKKTLVFYRGRAPRGQKTRWVMHEYRLDGTYAYHFLPGSTRDEWVIARVFQKPGEVPCGRKHRLGGPSAAAGESCFSDSTTSASIGGGGGGGASASSRPLLTVTDTSSPSLFVANANAAASNNNGNPVTGRELVPCFSTTASPLEAAALGVVGHPYNAAPLRLGLDFEAPSPGFVVPNLRSLQVQDDGGLPLFLSAAAGGGMSSATLGIMGSLGGSLHCPPHAGMDVVKVEGRAAPPQMAVGPGLLDGAFAWGF

>*At*CUC2|AT5G53950|*Arabidopsis thaliana*

MDIPYYHYDHGGDSQYLPPGFRFHPTDEELITHYLLRKVLDGCFSSRAIAEVDLNKCEPWQLPGRAKMGEKEWYFFSLRDRKYPTGLRTNRATEAGYWKATGKDREIFSSKTCALVGMKKTLVFYKGRAPKGEKSNWVMHEYRLEGKFSYHFISRSSKDEWVISRVFQKTTLASTGAVSEGGGGGGATVSVSSGTGPSKKTKVPSTISRNYQEQPSSPSSVSLPPLLDPTTTLGYTDSSCSYDSRSTNTTVTASAITEHVSCFSTVPTTTTALGLDVNSFSRLPPPLGFDFDPFPRFVSRNVSTQSNFRSFQENFNQFPYFGSSSASTMTSAVNLPSFQGGGGVSGMNYWLPATAEENESKVGVLHAGLDCIWNY

>*Br*CUC2|J00883|*Brassica rapa*

MDIPLYHYDHGGDSQYLPPGFRFHPTDEELITHYLLRKVLDGCFSSRAIADVDLNKCEPWQLPGKAKMGEKEWYFFSLRDRKYPTGLRTNRATEAGYWKATGKDREIYSSKTCALVGMKKTLVFYKGRAPKGEKTNWVMHEYRLEGKFSYHFISRSSKDEWVISRVFKKTGLANTGASGGEASASVSSCTGGSKKTKVPSTISTNYREQPSSPSSVSLPPLFDPTTTLGYTDSCYSYNSRSSNTTLTATAITEHVSCFSTATTTTASGLDVNVDSFNHLLPPAPPGFDHFSRFGSRNVSTLSNIRSFQENFNHFPYFGSSSASTMTPSVNLPSSHGGTGMNYWLQTTAEENETKAGLLNGGLDCVWNY

>*Eg*CUC2|Eucgr.B00529|*Eucalyptus grandis*

MENMARLGKEDDQIELPPGFRFHPTDEELITHYLQKKVGDTGFSAKAIGEVDLNKSEPWDLPWKAKMGEKEWYFFCLRDRKYPTGLRTNRATESGYWKATGKDKEIYRGKSLVGMKKTLVFYRGRAPKGEKTNWVMHEYRLEGKLSLNYLPRASKNEWVICRVFQKSSGGKKIHISSLVAAGSLENEMSSGLPPLTDSSPHDSKTESNPGSAYVPCFSSPTEFERNKENTNNYFNNPMFPISSNPTNTTPKISLLSPVYPHQAIPVPANWQHPGGSVFMPEHSVLRALLEGTGLNARQSARAEREAISISQETALTNDLNTEISSVMQDFEMGRRQFEDQQQVPSTLAGPMDVDLLWNYSS

>*Gm*CUC2|Glyma.13G274300|*Glycine max*

MDNSSYHHLDHTEAHLPPGFRFHPTDEELITYYLLKKVLDSTFTGRAIAEVDLNKSEPWELPEKAKMGEKEWYFFSLRDRKYPTGLRTNRATEAGYWKATGKDREIYSSKTCSLVGMKKTLVFYRGRAPKGEKSNWVMHEYRLEGKFAYHYLSRNSKDEWVISRVFQKSNTATNNGGSVMSASSNSKKTRMNSTTSLIHEPSSPSSVFLPPLLDTSPYTNTANFTDRHNGSYDSITKKEHVSCFSTIAAATTAVVSPNNFNNAGFDLSPSQPLATDPFARFQRNVDFSAFPSLRSLQDNLQFPFVFSTAAPPFSGGGSGDFLSWPVPEEQRLIDGVSNMPLGVSELDCMWSY

>*Gr*CUC2|Gorai.013G171300|*Gossypium raimondi*

MDSYHHFDNGETHLPPGFRFHPTDEELITYYLVKKVLDRSFTGRAIAEVDLNKCEPWELPDRAKMGEKEWYFFSLRDRKYPTGLRTNRATEAGYWKATGKDREIYSSKTCALVGMKKTLVFYRGRAPKGEKSNWVMHEYRLEGKFAYHYLSRSSKDEWVISRVFQKSSGGAKKAPMSAASMVLYQEPSSPSSVSLPPLLDTTNATGSGTATGASLTDRDSCSYDSHNQSEHVSCFSTIAATSSATLPGYHSGFDLALPTPPQMNNSFDSIARYTRNVGVPVFPSLRSLEENLQLPFYFSEPTLAGAAPPLDGGSSANWGAVSEEGNSGSVADGKMSNIGPTELDCMWTY

>*Cs*CUC2|orange1.1g047710m|*Citrus sinensis*

MEITYNYFDNSDAHLPPGFRFHPTDEELITYYLLKKVLDCNFTGRAIAEVDLNKCEPWELPAKAKMGEKEWYFFSLRDRKYPTGLRTNRATEAGYWKATGKDREIYSSKTCALVGMKKTLVFYRGRAPKGEKSNWVMHEYRLEGKFAYQYLSRSSKDEWVISRVFQKSSGAIATAAAVANAVKKSRLSCTISSSSTFNHSYPEPSSPSSVSLPPLLDHPTIAAAANATTAPNDSCSYDESHAPSDQHVSCFSTIAAAAAAAAASAATATTFNTSSSAFDFTTVPAPVINADAGAGAACDPFARFGRNNVGLNAFPNLRSLQENLQLPFFFAPPASSVAPPPFQGGGGGSNWSTVMQDIGGGGGVVGGGGRLNVGPTELDCMWTY

>*Pv*CUC2|Phvul.005G074500|*Phaseolus vulgaris*

MDSSYHHLDHTEAHLPPGFRFHPTDEELITYYLLKKVLDSTFTGRAIAEVDLNKSEPWELPEKAKMGEKEWYFFSLRDRKYPTGLRTNRATEAGYWKATGKDREIYSSKTSSLVGMKKTLVFYRGRAPKGEKSNWVMHEYRLEGKFAYHYLSRNSKDEWVISRVFQKSNTSNGGSAMSASSGSKKTRMNTTNSSLCPEPSSPSSVYLPPLLDSSPYANTTTAVNFTGRNNCSYDSTTKKEHVSCFSTIAAATAAVVSPNNFTNASFDLPPSQSLGTDPFARFQRNVGVSAFPSLRSLQDNLQLPFFFPPAAQPFSVSGTGDLLWPMPEEQRLVDAASNVPLGVSELDCMWGY

>*Sl*CUC2|Solyc07g062840|*Solanum lycopersicum*

MEIYHQMQFDCGDPHLPPGFRFHPTDEELITYYLLKKVLDCNFTARAIAEVDLNKCEPWELPGKAKMGEKEWYFFSLRDRKYPTGLRTNRATEAGYWKATGKDREIFSSKTCALVGMKKTLVFYRGRAPKGEKSNWVMHEYRLDGKFAYHYISRSSKDEWVISRVFQKSTGSNGAATSTGGGKKRLSSSINMYQEVSSPSSVSHLPPLLDSSPYSTTATSAAAIVIGDRDRDHSFKKEHVPCFSTTATATITAQSLTFDPTSVFDISSNTLHALQPTPSFASILDSSPSNFTNYTRNSTFPSLRSLHENLQLPLFSGGTSAMHGGFSNPMVNWTVPETQKVEQSELDCMWSY

>*Dc*CUC2|DCAR_019571|*Daucus carota*

MDHFYQSMENNGDAQLPPGFRFHPTDEELITYYLLKKVLDHNFSSRAIAQVDLNKCEPWHLPEKAKMGEKEWYFYSLRDRKYPTGLRTNRATEAGYWKATGKDREIYSSKTSSLVGMKKTLVFYRGRAPKGEKTNWVMHEFRLDGKLAYHYLSTTSKDEWVISRLFKKTGGATAGEKRPSSSMSSHFHSEISSSSSIPFTPPPPPATTTDHVITYEHVPCFSSSAAPGGFSTYHTLFDGGLPPPLMDPTPMPPSSTFPSLRSLEENLHQPSFFFPPVNYDNFPAMETPKPGLTELDCIWRPSFN

>*Ss*CUC3| Sspon.06G0001780-1A|*Saccharum spontaneum*

MHHHQAMSDALWDLLGEEMAAAGGEHGLPPGFRFHPTDEELVTFYLAAKVFNGACCGIDIAEVDLNRCEPWELPDAARMGEREWYFFSLRDRKYPTGLRTNRATGAGYWKATGKDREVLNAATGALLGMKKTLVFYKGRAPRGEKTKWVLHEYRLDGDFAAARRPCKEEWVICRILHKAGDQYSKLMMVKSPYYLPMAMDPSSFCFQQDPTAPPLQNPSGCIPFQHGHPSMQPPPLPPSNHGKVVFTGAAAPCMQQEPANGSNSAVLPMPPLPHFTPIVAGKPAPAPPPQVGVNAGPQEPPPPPPTWLEAYLQHGGGFLYEMGPAAAPRGA

>*Ac*CUC3|Aco000744|*Ananas comosus*

MHTNMGELVWELFGEETYNEQGLPPGFRFHPTDEELVTFYLASKVFNGGLCGVDIVEVDLNRCEPWELPDAAKMGEREWYFYSLRDRKYPTGLRTNRATGAGYWKATGKDREVHGAANGALVGMKKTLVFYKGRAPRGEKTKWVLHEYRLEGDYACRHRCKEEWVICRIFHKTGGDKKNQYYPNPSYTINPSSSTPSTCILPFLDPQTLETPLQTLHNHHQPYFHLNQEPINPLFPLPPLPSFACSSTFLPSFPKSPPKEEDTNALLNPNEEAMFPANWLETYIQNPFVYEMGFSLPGPGAPVYDVPLLGYTATGESGPL

>*Os*CUC3|LOC_Os08g40030|*Oryza sativa*

MGDALWEMLGEEMAAAAAAAGEHGLPPGFRFHPTDEELVTFYLAAKVFNGACCGGVDIAEVDLNRCEPWELPEAARMGEKEWYFFSLRDRKYPTGLRTNRATGAGYWKATGKDREVVAAAAAGGALIGMKKTLVFYKGRAPRGEKTKWVLHEYRLDGDFAAARRSTKEEWVICRIFHKVGDQYSKLMMMKSPASYYLPVSHHHPSSIFHDLPPVPFPNPSLVPFHHDLPTSFHPPLLQHSHANSKNSSSNNGGFVFPNEPNTTNSSDNHISCNGAMAAAAAAAFPSFSCASTVTGKGGPPAQLGVNAGQQEPPPPTWMDAYLQHSGFIYEMGPPAVPRGA

>*Bd*CUC3|Bradi3g40085|*Brachypodium distachyon*

MHQHQHPAAAAMGAEALWDMLSEDMAAAAAAAAEHGLPPGFRFHPTDEELITFYLAPKAFNSSNDNDSNFSAVDFIAEVDLNRCEPWALPESARMGGEREWYFFSLRDRKYPTGLRTNRATGAGYWKATGKDREVVCAATGALIGMKKTLVFYEGRAPRGHKSKWVLHEYRLDGDFAADRRSCKEEWVVCRILHKTVDQYSSKMMEMRMMSPYHHCYHPMSHHHHPSFVFQDAPPVPFPNPSGQLPVPFLHHHHDLIPNLQQPSPLTTQHHHQPQAADKNSSSNNGGFPVPAAAAACIQDQQPDNNTAPYFPFPSLASAVTVAAKAGPLPGVNAAGPQELLPPTWPLDNFLQHGIATYLYETGPPAGAPRDA

>*Hv*CUC3|MLOC_13932|*Hordeum vulgare*

MHQHQPAAAMGDALWELIGEEMAAAEAAAGEHGLPPGFRFHPTDEELVTFYLAAKVFNGTCCGGVDIAEVDLNRCEPWDLPEAARMGEREWYFFSLRDRKYPTGLRTNRATGAGYWKATGKDREVLNAATGSLLGMKKTLVFYRGRAPRGEKTKWVLHEYRLDGDFGAVRRSCKEEWVVCRIFHKAVDQYSKMMEMRNPYCYFPMTPHHPSFFQDAPPVPFPNPSQLIPFHHDLPHLQSSPLIQGQAKNTSNNSNGGFPAAASIQEQPNSSCNPAYFPFPSFASIVNGKAGPPAQPGVNGGPQEPPPTWLDACLQHSAFMYEMGPPAATRGA

>*Zm*CUC3|GRMZM2G430522_P01|*Zea mays*

MVLWRTGGAWWCYLARAPHYKAPPHTIPQLRASSHHLVERKSESEVGKGIGFLVDQIPFPSWILIFLLRSHQIDLTTCLRSKPLCMHHHHQDQAMGDALWDLLGEEMAAAGGEHGLPPGFRFHPTDEELVTFYLAAKVFNGACCGIDIAEVDLNRCEPWELPDAARMGEREWYFFSLRDRKYPTGLRTNRATGAGYWKATGKDREVLNAATGALLGMKKTLVFYKGRAPRGEKTKWVLHEYRLDGDFAAARRPCKEEWVICRILHKAGDQYSKLMMVKSPYYLPMAMDPSSFCFQEDPTGHPLPNPSGCTPFHHGHPHHSMQPPPPLPPSNHAGKAVFTGAAAACCMQQEPADGSNSAVLPMPPFPPFTPIVAGKPAAPAPPPQVVNAGPQEPPPPTWLEAYLQHTGGILYEMGPTAAPRGA

>*At*CUC3|AT1G76420|*Arabidopsis thaliana*

MMLAVEDVLSELAGEERNERGLPPGFRFHPTDEELITFYLASKIFHGGLSGIHISEVDLNRCEPWELPEMAKMGEREWYFYSLRDRKYPTGLRTNRATTAGYWKATGKDKEVFSGGGGQLVGMKKTLVFYKGRAPRGLKTKWVMHEYRLENDHSHRHTCKEEWVICRVFNKTGDRKNVGLIHNQISYLHNHSLSTTHHHHHEALPLLIEPSNKTLTNFPSLLYDDPHQNYNNNNFLHGSSGHNIDELKALINPVVSQLNGIIFPSGNNNNDEDDFDFNLGVKTEQSSNGNEIDVRDYLENPLFQEASYGLLGFSSSPGPLHMLLDSPCPLGFQL

>*Br*CUC3|Brara.G03438|*Brassica rapa*

MMLAVEDVLSELAGEERNDRGLPPGFRFHPTDEELITFYLASKVFHGGLCGIHIAEVDLNRCEPWELPEMAKMGEREWYFYSLRDRKYPTGLRTNRATTAGYWKATGKDKEVFAGGGGGGGALVGMKKTLVFYKGRAPRGLKTKWVMHEYRLETDLSHRHTCKEEWVICRVFNKTGDRKNVGIHNQISYLHNTSLSTTHQQHNHYHHLEILPPLLEPSKTLTNFPSLLYDDTHQNYNNNLLHGSSAHNVDEFKTLINPAVSQLNGVIFSPENSNYNNEDDNNFGVKTEQYSNGGNNDLDVRDYLDNPFCQEAGYGLLGLSSSPGPLMLLDSPYVL

>*Dc*CUC3|DCAR_013939|*Daucus carota*

MLAIEEILCELNGSEMNEQGMPPGFRFHPTDEELITFYLASKVYNGSFCGVDIAEVDLNRCEPWELPDIAKMGEREWYFFSLRDRKYPTGLRTNRATGAGYWKATGKDREVYSSSANGGAATLLGMKKTLVFYKGRAPRGEKTKWVMHEYRLDGDFSCRHTCKEEWVICRIFQKIGEKKNGGLLQGQSSSSSYMQEASNSSLSRLFDQSLKSGSALSLQPYHTLQSLQNQNQNRSQVLINNIPHEADLKSLITNSSSSSLAVSQASPFPVNNELNGLQTSCSPPKTKMKQDHNLLKTLLPHQDYYCPKEQEEAPFPKICKTESNFSHFQSPHFSTHPHIPNFRFPISTTAEYDMNLAHQITNTPNYKQSPLLFRSLDSDTKNVMGNNCGVGLGSCGFPAYGTGDTEMSTSSSSCSRGLPFSRAGFKQMLLLDPPTKMSAGESWPFHF

>*Eg*CUC3|Eucgr.F03588|*Eucalyptus grandis*

MLGVEDLLCELRREEGNEQGLPPGFRFHPTDEELITFYLASKVFNGAFSGLDIAEVDLNRCEPWDLPEVAKMGEREWYFFSLRDRKYPTGLRTNRATGAGYWKATGKDREVHSAATGALLGMKKTLVFYRGRAPRGEKTKWVMHEYRLDGHFSCRHTCKDEWVICRIFHKTMEKKSLLFQGQNYMLEVCSPPSTGSLPQLLETPITSTPPPNHLLEHQSQANITTTTHKDSYNNTDTKNIIVTPSLVSPSSMLFKSLLSHQDLFTATNLKEHQHHTVLNQCKTETNFSHFQLNVDDDNDADGLHSVVDKIHIVPFHQNHQSPVLFEMDTHPTTTSSVPDNNNAPHGHTVHHEMSTSTMAFNRVGPSFQMLVDPAFRESWPLDP

>*Gm*CUC3|Glyma.06G014900|*Glycine max*

MLAMEELLYELSDHERRNEQGLPPGFRFHPTDEELVTFYLASKVFNGTFSNVKFAEVDLNRCEPWELPDVAKMGEREWYLFSLRDRKYPTGLRTNRATGAGYWKATGKDKEVYSASSGTLLGMKKTLVFYKGRAPRGEKTKWVMHEYRLVLDAHFSLPHTHPSKEEWVICRIFHKSGEKRSPVLQVHGHSDASSSPRESALPPLLASPSCFTFDPESQSQSSSHSQRDFQSPVLIHHQDQNGHSHNPRLFPLEITNARNHPSSFSDLFFKPLQQNCTLKTNEQTILPKVTKTEDATFYDQYHQLLDDHNNMRWVNKLNQNPSNLLNTFPFEVDAGLMAFSGAANAQVKDISTSTPFNRVGLQQTLDSWPLAQHV

>*Gr*CUC3|Gorai.002G113300|*Gossypium raimondi*

MFLHSFQSQQLAFTLNKMLAVEEVLSELGGEEVNEQGLPPGFRFHPTDEELITFYLASKVFNGSFCGVDIAEVDLNRCEPWELPDVAKMGAREWYFFSLRDRKYPTGVRTNRATGAGYWKATGKDREVYSASTGALLGMKKTLVFYNGRAPRGVKTMWVMHEYRLDGDFSCRHTCKEEWVICRIIHKTGEKKNGVAAAQGLGYILELSSLSSTTKTTNCLRPLLETPTPLLESQTQISMQAAHNSFLENDLKSLINPVVYPANGFQPSFTATPTTFSSTPDKNASSNSSAAASMLFKSLLSHQECVLREQAAATIPKQCKTEANFSNFQLPDSTLSWTEKMHPNPCQDPMFFDMDYNNSVLGFAEL

>*Cs*CUC3|orange1.1g016283m|*Citrus sinensis*

MEDVLSELNGDEVNEQGLPPGFRFHPTDEELITFYLASKVFNGTFCGVEIAEVDLNRCEPWELPDVAKMGEKEWYFFSLRDRKYPTGLRTNRATGAGYWKATGKDREVCSGSSGALLGMKKTLVFYKGRAPRGEKTKWVMHEYRLDGDFSYRHTCKEEWVICRIFNKAVVSGEKKNGLLLQGQHYLFEAAATAGACLPALLDAPGPATTTLLLECQSQNHNPILENLPNHFVNQQQDNHHHHLFPVNGLFETSAVTNKHILINNITENIGNNTSPSMLFKALLSHQDFSCCNELAPSPKHCKTEANFSHIQLPPATAADDNSNDNWSNCYWMDSKIQPNPYSNPLFSEFDCSFPGLTQPSAFAATAVNDMSTSIAFNRTGFQVVEKSWPLGA

>*Pv*CUC3|Phvul.009G008000|*Phaseolus vulgaris*

MLAMEDLLCELSDHEKRNEQGLPPGFRFHPTDEELITFYLASKVFNGSFTSVKFAEVDLNRCEPWELPDVAKMGEREWYLFSLRDRKYPTGLRTNRATGAGYWKATGKDKEVFSASSGTLLGMKKTLVFYKGRAPRGEKTKWVMHEYRLDGDFSLPHPHPHHISKEEWVICRIFHKSGEKRTPLVQVQGHSDASSSPTKNSLPPLLASPTCFTLELECQSQQSPVLIHHHQDQNHLSHSHPYLFPLHASPQLTNARNHPSFSDLFFKPLHNSQQNCIFKAKEKTAPKVVKTEEATAFYQYHLLGDANNLRVNQNPSNFPNPFPDVEVDAGLMAFSGGPNAEVRDMSTSTAFNRVGLQQVIDAAHIGIDSWPLPQHV

>*Sl*CUC3|Solyc12g036480|*Solanum lycopersicum*

MDENLPPGFRFHPTDEELITCYLNNKISDFNFTTRAIADVDLNKSEPWDLPAKASMGEKEWYFFSLKDRKYPTGLRTNRATEAGYWKTTGKDKEIYRGGTGVLVGMKKTLVFYRGRAPKGEKTNWVMHEYRIETTFGYKPSKEEWVVCRVFQKSSTVKKPQPTSSSPLSLESPCDTNYTITNELGDIELPFNFNYLTTTPSTAINNISLHNYNNDNINLAAATREANSHPLLPWSSNLLSSNLSSVNSLLFRALQLKSYSPREQATTTHDYAFMLPQENIITTQFGNDFAVNNIGAPSSSTVLDNSVQQQQQQQQEQSYKLDSNIW
