# Supplementary material for: Interspecific complementation-restoration of phenotype in Arabidopsis cuc2cuc3 mutant by sugarcane CUC2 gene
Source: BMC Plant Biol. 2022 Jan 22;22:47. doi: 10.1186/s12870-022-03440-z (PMC8783490; doi:10.1186/s12870-022-03440-z)
Supplement: Supplementary file 5 — Additional file 5. List of primers used in the present study. [file 12870_2022_3440_MOESM5_ESM.docx]

Additional file S4: List of primers used in the present study.

| Sl.No. | Name | Primer Seq 5-->3 |
| --- | --- | --- |
| Primers used for cloning SsCUC2 in pENTR vector | | |
| 1 | SsCUC2 F | caccATGGAGCGGTTCGGCGTGCT |
| 2 | SsCUC2 R | GTAGCCCCAGCCGAAGGCGC |
| Primers used for cloning SsCUC2 in pGBKT7 vector | | |
| 3 | SsCUC2 F | catggaggccgaattcATGGAGCGGTTCGGCGTGCT |
| 4 | SsCUC2 R | gcaggtcgacggatccGTAGCCCCAGCCGAAGGCGC |
| Primers used for RT-qPCR | | |
| 5 | SsCUC2 F | GATCGACCTCAACAAGTGCG |
| 6 | SsCUC2 R | CGGAGGCTGTAGAAGTACCA |
| 7 | SsCUC3 F | TGCAAGGAGGAATGGGTGAT |
| 8 | SsCUC3 R | GGTAGTAGGGGCTCTTCACC |
| Primers used for RT-qPCR of miR164a | | |
| 9 | RT primer | GTCGTATCCAGTGCAGGGTCCGAGGTATTCGCACTGGATACGACTGCACG |
| 10 | Forward | GTATACTGGAGAAGCAGGGCA |
| 11 | Reverse | GTGCAGGGTCCGAGGT |
